# Back pain exercise therapy remodels human epigenetic profiles in buccal and human peripheral blood mononuclear cells: an exploratory study in young male participants

**DOI:** 10.3389/fspor.2024.1393067

**Published:** 2024-10-16

**Authors:** Claire Burny, Mia Potočnjak, Annika Hestermann, Sophie Gartemann, Michael Hollmann, Frank Schifferdecker-Hoch, Nina Markanovic, Simone Di Sanzo, Michael Günsel, Victor Solis-Mezarino, Moritz Voelker-Albert

**Affiliations:** ^1^ EpiQMAx GmbH, Planegg, Germany; ^2^ Moleqlar Analytics GmbH, Munich, Germany; ^3^ FPZ GmbH, Cologne, Germany

**Keywords:** histone modifications, plasma proteome, epigenetics, sport therapy, lifestyle exposome, data integration, biomarker, back pain

## Abstract

**Background:**

With its high and increasing lifetime prevalence, back pain represents a contemporary challenge for patients and healthcare providers. Monitored exercise therapy is a commonly prescribed treatment to relieve pain and functional limitations. However, the benefits of exercise are often gradual, subtle, and evaluated by subjective self-reported scores. Back pain pathogenesis is interlinked with epigenetically mediated processes that modify gene expression without altering the DNA sequence. Therefore, we hypothesize that therapy effects can be objectively evaluated by measurable epigenetic histone posttranslational modifications and proteome expression. Because epigenetic modifications are dynamic and responsive to environmental exposure, lifestyle choices—such as physical activity—can alter epigenetic profiles, subsequent gene expression, and health traits. Instead of invasive sampling (e.g., muscle biopsy), we collect easily accessible buccal swabs and plasma. The plasma proteome provides a systemic understanding of a person's current health state and is an ideal snapshot of downstream, epigenetically regulated, changes upon therapy. This study investigates how molecular profiles evolve in response to standardized sport therapy and non-controlled lifestyle choices.

**Results:**

We report that the therapy improves agility, attenuates back pain, and triggers healthier habits. We find that a subset of participants' histone methylation and acetylation profiles cluster samples according to their therapy status, before or after therapy. Integrating epigenetic reprogramming of both buccal cells and peripheral blood mononuclear cells (PBMCs) reveals that these concomitant changes are concordant with higher levels of self-rated back pain improvement and agility gain. Additionally, epigenetic changes correlate with changes in immune response plasma factors, reflecting their comparable ability to rate therapy effects at the molecular level. We also performed an exploratory analysis to confirm the usability of molecular profiles in (1) mapping lifestyle choices and (2) evaluating the distance of a given participant to an optimal health state.

**Conclusion:**

This pre-post cohort study highlights the potential of integrated molecular profiles to score therapy efficiency. Our findings reflect the complex interplay of an individual's background and lifestyle upon therapeutic exposure. Future studies are needed to provide mechanistic insights into back pain pathogenesis and lifestyle-based epigenetic reprogramming upon sport therapy intervention to maintain therapeutic effects in the long run.

## 1 Introduction

On an epidemiological level, physical exercise and balanced nutrition are the most effective preventive and curative choices for many chronic diseases. Physical activity is associated with a range of health benefits; it improves the overall blood profile, leads to lower blood pressure, has a positive influence on the immune system, and reduces inflammation. Along with physical benefits, it leads to a range of mental health benefits, including reduced depressive disorders, improved cognitive function, and overall feeling of well-being (1, 2). Sport therapy also improves flexibility and relieves chronic back pain, a prevalent condition worldwide. In a French population survey, nearly 40% of people reported chronic back pain and severely reduced quality of life (3, 4). Between 1990 and 2015, disability caused by low back pain rose by 54%, accounting for 60.1 million disability-adjusted life years in 2015. Back pain is a leading reason for living with a disability in both sexes (5, 6). In addition to the strong negative influence of back pain on healthy living years, recent studies also show the positive effects of strength training on this factor (7, 8).

On a molecular level, physical exercise is associated with gene expression to adapt to the necessary metabolic changes. In particular, exercise modulates insulin regulation, glucose, lipid metabolism, mitochondrial metabolism, and immune competency (9–11). Regulation of gene expression is driven by epigenetic processes and chromatin remodeling. Epigenetic regulation involves DNA methylation, miRNA expression, and histone modifications (12–15). The most studied histone modifications are methylation and acetylation, which lead to different degrees of chromatin condensation (16, 17). Epigenetic modifications are reversible; some alterations driven by physical activity will regress if exercise is not performed regularly (18–21)*.* Recent work has shown that epigenetic changes may be important in regulating skeletal muscle adaptation to physical exercise. There have been several studies on the influence of sport on muscular health, predominantly using tissue biopsy, an invasive and costly technique that not many people find agreeable (22, 23). Previous studies demonstrated that a single bout of exercise vs. a prolonged training session led to different epigenetic reprogramming (22). Acute rounds of cycling exercise increased H3 acetylation at lysine 36 (K36), a site associated with the activation of transcription (24). Similarly, a study contrasting acute vs. resistance exercise training on skeletal muscles pinpointed enhanced acetylation of histone H3 in both cases with a substantial increase in methylation at lysine 4 (K4) and 27 (K27) of histone H3 during acute rounds of exercise (22). However, the effects of these local muscular changes have not been evaluated in samples outside the muscle tissue. Measuring epigenetic changes in easily accessible samples, such as mouth swabs and peripheral blood, allows for timely measurement of the effects of exercise in human organisms. Here, we use buccal cells, peripheral blood mononuclear cells (PBMCs), and plasma to characterize a range of epigenetic and proteomic histone biomarkers involved in an individual's fitness status using high-throughput mass spectrometry (25, 26). Such measurement methods can facilitate the development of optimized and individual training strategies and promote a healthy lifestyle (25, 26).

## 2 Material and methods

### 2.1 Study participants

Participants were recruited via FPZ's social media channels and by directly approaching the companies in the immediate vicinity of the therapy center conducting the study. The content and procedure of the study were explained to the interested persons in a short telephone call. In the case of interest and matching enrollment criteria, male participants aged 25–35 years with average or low physical condition (not participating in professional sport) were included in the study. Reasons for exclusion from the study were certain spinal and bone disorders and certain diseases that are identified as contraindications from participating in sport therapy. A detailed exclusion list is reported in Supplementary Material 1 (Supplementary Table S1).

### 2.2 FPZ therapy program and agility metrics collection

**Figure 1:**
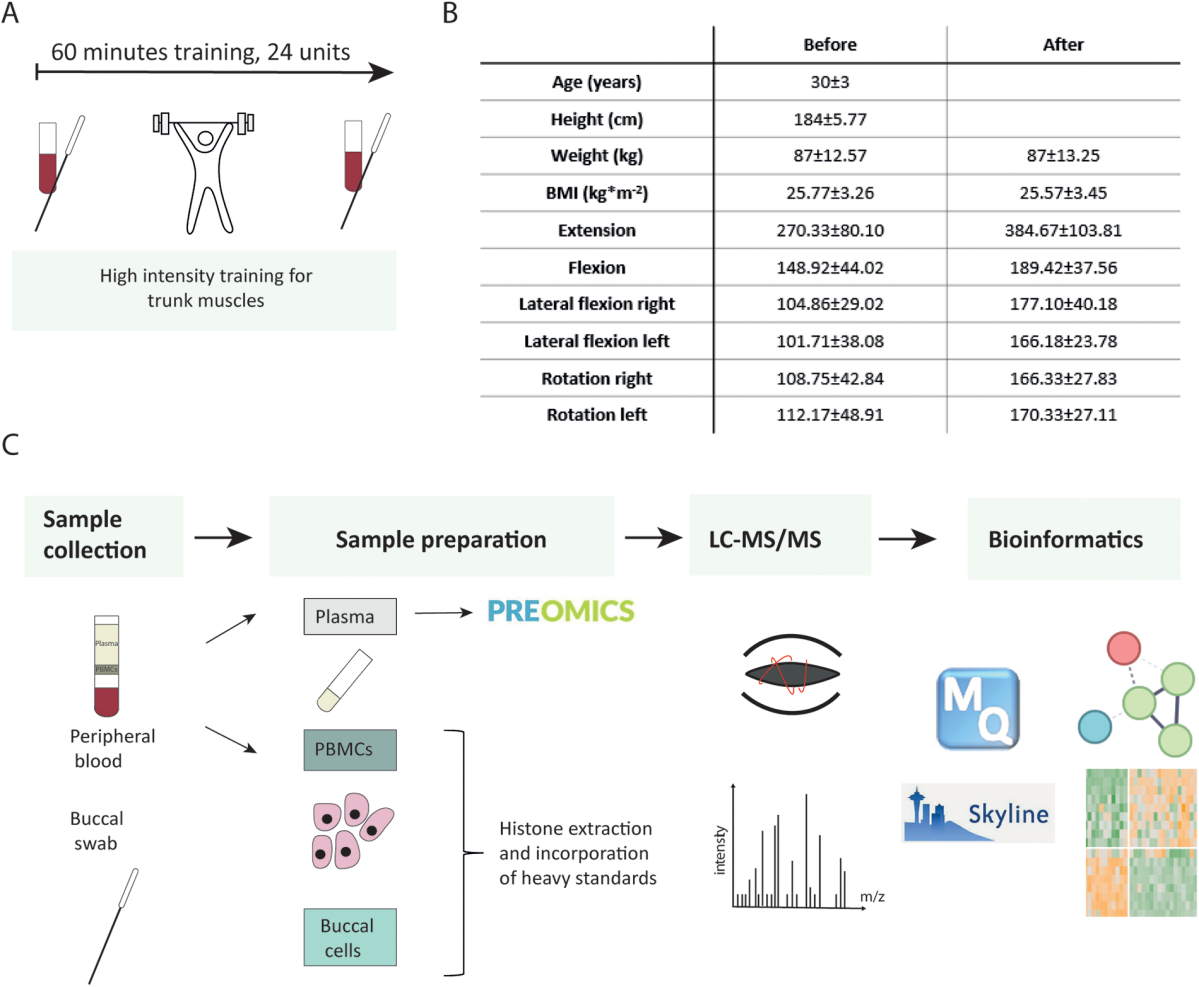
Study flow chart. **(A)** Participants underwent high-intensity training for trunk muscles. Their blood and buccal swabs were collected before and after completion of 24 therapy sessions. **(B)** Subjects’ characteristics. **(C)** Analytical workflow.

The participants took part in a functional back pain therapy program focusing on the strengthening of trunk muscles, which included 24 training units of 60 min each (FPZ Therapie, www.fpz.de) in a certified FPZ center (Figure 1). It was recommended that the training units be carried out twice a week to achieve an optimal training effect (27). After a short warm-up phase (10 min), the training session consisted of high-intensity training of specific trunk muscles. Special training devices with integrated measuring devices were used to allow the isolated control of the muscle groups to be trained in extension, flexion, lateral flexion, and rotation. Short-duration stretching and relaxation exercises were carried out between the training impulses on the individual machines. At the end of each training session, additional cool-down and relaxation exercises were done.

Before entering the study [before therapy (BI)] and after finishing the therapy program [after therapy (AT)], we measured the isometric maximum strength in all directions of movement to be trained. The therapist additionally compiled detailed questionnaires regarding back pain history, lifestyle habits, and quality of life scores. According to the results of this assessment, an individual training plan was developed.

### 2.3 Questionnaire data management

Out of 19 male participants who signed up for the study, we evaluated the data of 12 participants who completed the therapy, corresponding to a dropout rate of 39%. All 12 individuals followed at least 20 out of 24 sessions (7 completed the full number of sessions) from 16 to 25.6 weeks. Three types of variables were collected in the questionnaire: (i) those directly targeted by the therapy (agility metrics and quality of life scores), (ii) lifestyle variables that might change upon therapy [e.g., nutritional choices, level of physical activity, body mass index (BMI), demographic parameters], and (iii) lifestyle history indicators not modifiable throughout the therapy (e.g., back pain history).

**Figure 2:**
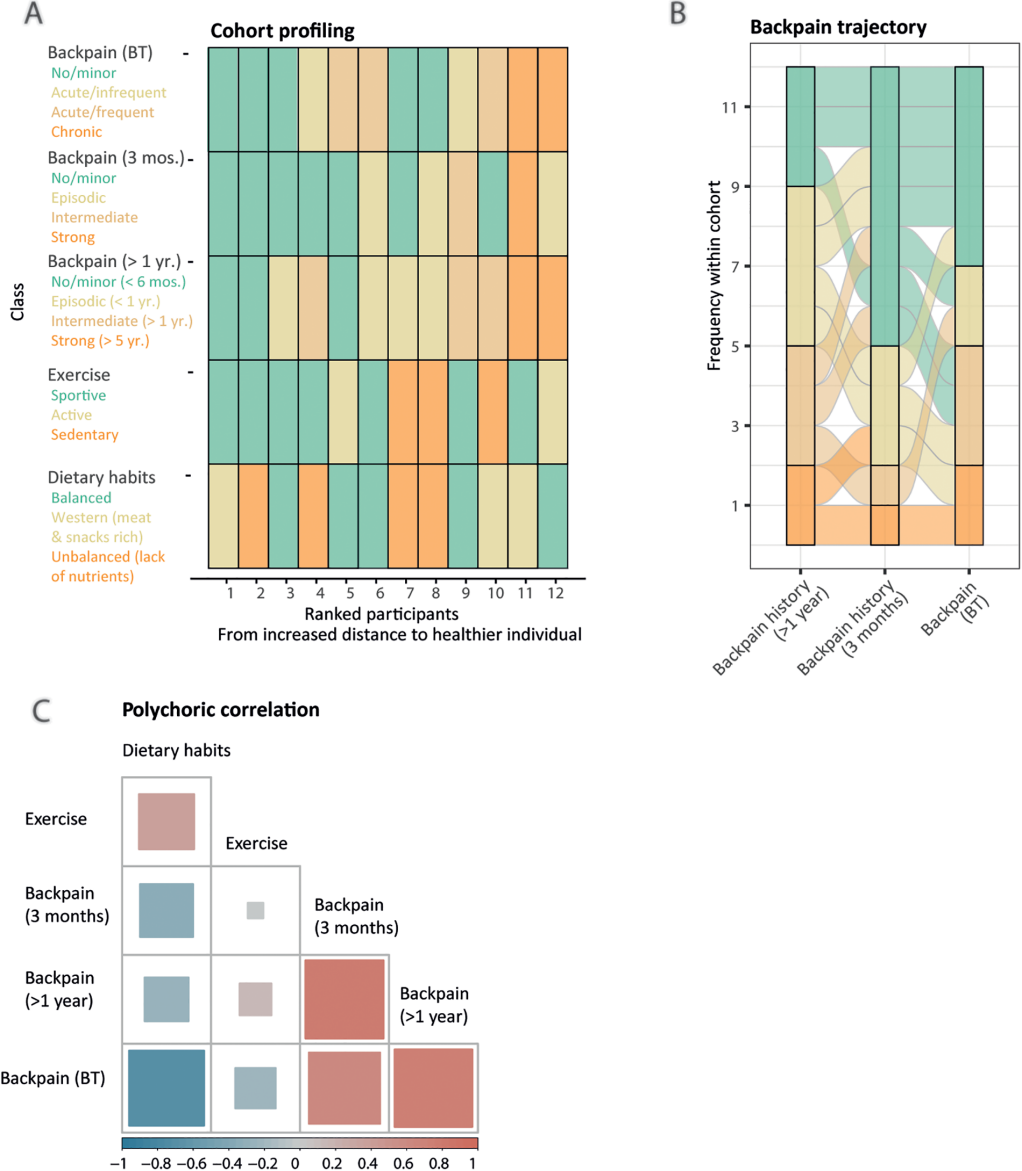
Latent lifestyle categorization reveals similar patterns of answers. **(A)** Lifestyle sequence plot with respective categories (legend, *y*-axis), obtained from partitioning around medoids clustering, colored-coded from less healthy (orange) to healthier (green) for each participant (*x*-axis). Participants are ranked by increased Gower distance to the healthier participant (labeled as 1). **(B)** Alluvial plot displaying the cohort's back pain history over 1 year, 3 months, and at entry of the therapy (*x*-axis). A participant's trajectory is represented as an alluvial. Longitudinal clustering of the cohort's back pain history indicates four categories: mild/recovering (*n* = 5), fluctuating (*n* = 4), severe/chronic (*n* = 2), and worsening (*n* = 1). **(C)** Pairwise polychoric correlation matrix indicating dependence between different lifestyle components. The squares’ areas are proportional to the absolute value of the correlation coefficient for the given pairwise comparison and the color indicates the sign, either negative (blue) or positive (red) correlation.

Following imputation and data wrangling, questions were grouped into six groups of information (Supplementary Material 1: Supplementary Methods, Supplementary Tables S2–S8) to identify homogeneous clusters of participants that share either similar habits (exercise, dietary habits), background (long- and short-term back pain history, back pain status before therapy, summarized individually by an alluvial plot) or comparable therapy response (agility metrics, back pain status after therapy). Except for the agility metrics, we used the partitioning around medoids algorithm, supported by the silhouette width coefficient, to create clusters of participants, and reported summary statistics, such as the mean and medoid of each answer per cluster to interpret and label the categories (Figure 2A, Supplementary Material 1: Supplementary Table S9, Supplementary Material 2: Supplementary Figures S1–S3) (28).

**Figure 3:**
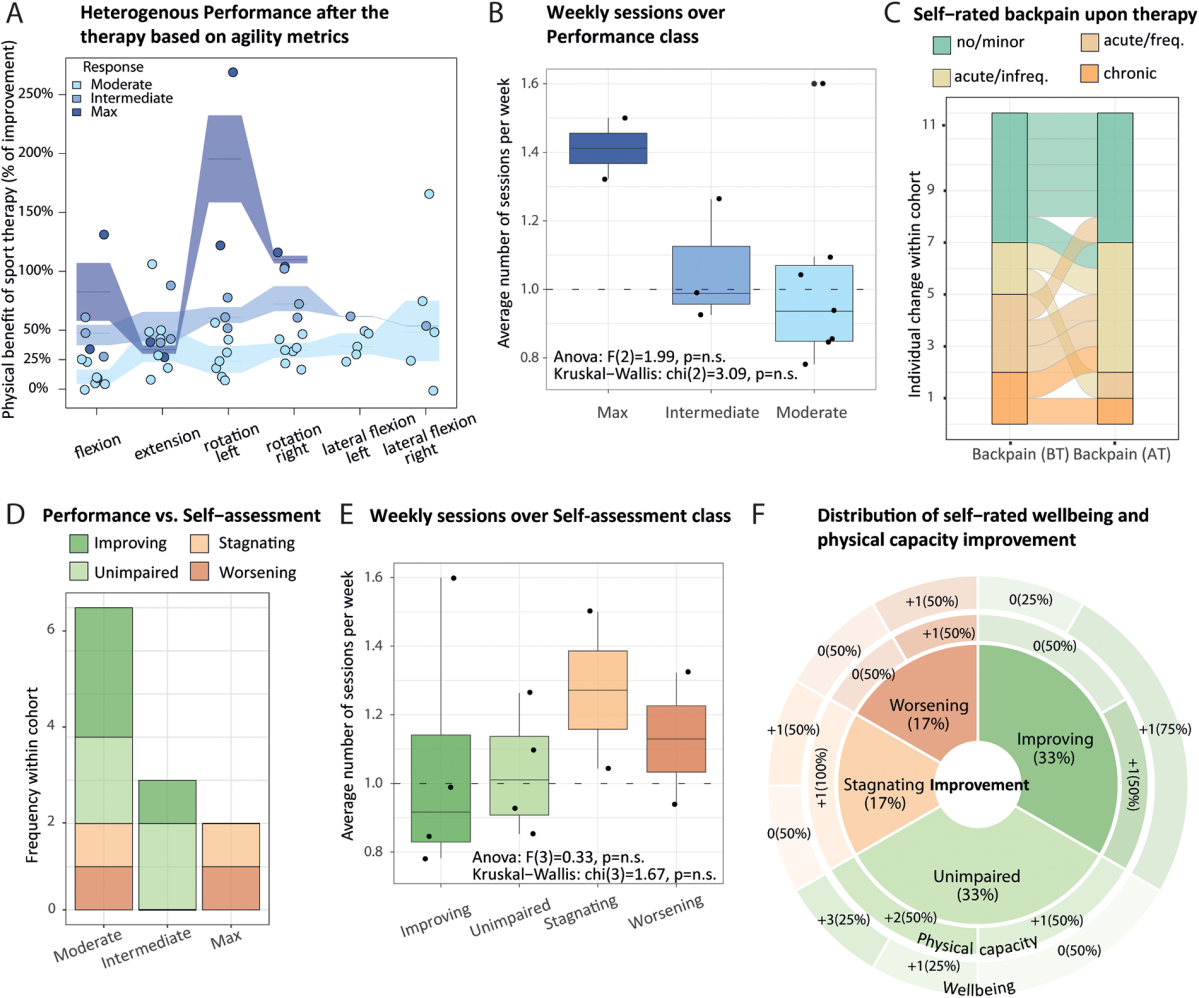
Agility metrics changes and back pain self-assessment reflect adverse therapy effects that depend on lifestyle. **(A)** All participants responded positively to the therapy. Interquartile range (intervals) and median percentage of improvement (segments) for respective agility metrics (*x*-axis) revealed moderate, intermediate, and max (light to dark blue, respectively) performance after the therapy (*y*-axis). One dot represents one participant, whose classification was obtained from baseline-adjusted agility metrics (using paired flexion, extension, and rotation measurements), applying the Gaussian mixture modeling framework. **(B)** Boxplot of the average number of weekly therapy sessions (*y*-axis) over performance categories (*x*-axis) across participants (dots), with reported Kruskall–Wallis test and ANOVA outcome. **(C)** Alluvial plot displays changes in self-rated back pain (*x*-axis) before (BT) and after (AT) therapy [Friedman *χ*^2^ (1) = 0.67, *p* = 0.41]. **(D)** Contingency matrix between performance (*x*-axis) and self-assessment categories (stacked *y*-axis, color-coded) is displayed as a bar plot (no statistically significant association; Fisher's exact test; *p* = 0.67). Self-assessment indicates individual changes in back pain status upon therapy: improving, unimpaired (back pain remains minor), stagnating, worsening. **(E)** Similar to **(B)** but stratified by self-assessment. **(F)** Sunburst chart sliced by categories of back pain, self-assessment describing self-rated physical capacity (inner ring), and well-being (outer ring) gain, each measured on a 0–5 scale. Non-parametric Friedman test reports a significant improvement of both scores upon therapy [*χ*^2^ (1) = 9, *p* = 2.70 × 10^−3^, and *χ*^2^ (1) = 7, *p* = 8.15 × 10^−3^, respectively].

Furthermore, we displayed as an alluvial plot the back pain trajectories of the cohort through time, from yearly to 3 months, and at the study entry time points (Figure 2B). After each participant had been assigned to his corresponding category, we measured the interdependency of back pain history and lifestyle components using pairwise polychoric correlation (Figure 2C). To evaluate the therapy efficiency, we created two additional response classes. First, as back pain status class was defined from self-rated answers, we assessed if participants' category changed upon therapy either toward improvement, stagnation, or worsening (back pain self-assessment class). Second, we used the Gaussian Mixture Model framework on the percentage of change of the agility metrics upon therapy, using baseline-corrected metrics, to partition participants into moderate, intermediate, and max performance categories (Figure 3, Supplementary Material 1: Supplementary Tables S10, S11). To better understand the clustering of the participants into the different response categories, we performed a multiple factor analysis (MFA) using the COVID status and both response classes' categories as illustrative variables and the following sets of active qualitative variables: (1) sociodemographic environment (association of back pain with work, work position, relationship status and study level, four variables), (2) lifestyle (exercise and dietary habits classes, two variables), and (3) back pain history (last 12 months, last 3 months and at entry, three variables) (Supplementary Material 2: Supplementary Figure S4). Intermediate plots useful for the axes' interpretation as well as individual factor maps are represented in Supplementary Material 2 (Supplementary Figure S5).

### 2.4 Blood and buccal swab collection and separation

Venous blood was collected using BD Vacutainer CPT according to the manufacturer's instructions. The resulting PBMC cells and plasma samples were stored at −20°C for up to 8 weeks until further processing. Buccal cells were collected by hDNA-free FLOQSwabs (Copan Italia, Brescia Italy) swabbing each cheek 30× and stored at −20°C for up to 8 weeks.

### 2.5 Histone preparation from buccal swabs or peripheral blood mononuclear cells using in-gel digestion

Swab heads were covered with a hypotonic buffer containing dH_2_O, 1 mM DTT, and protease inhibitors (1:1,000) aprotinin, leupeptin, pepstatin, and PMSF for cell extraction from swabs. Swabs were incubated for 15 min at 4°C, shaking at 700 rpm, followed by centrifugation of 1,500 rcf and 4°C for 5 min and 10,000 rcf for 10 min in subsequent 1.5 ml tubes, respectively. Pellets were resuspended in 75 µl ice-cold 0.2 M H_2_SO_4_ and placed on the ThermoMixer (Eppendorf) at 4℃ and 1,000 rpm overnight.

Peripheral blood mononuclear cells (PBMCs) were resuspended in 500 µl 1× PBS TC to obtain a final cell count of 5.0 × 10^5^. The pellet was resuspended in 150 µl of cooled 0.2 M H_2_SO_4_ and sonicated using a Hielscher device with 60 s ON and 60 s OFF settings for 20 min. Samples were acid extracted overnight at 4℃ and 1,000 rpm in a ThermoMixer.

Both swab and PBMC samples were then centrifuged for 30 min at 16,000 rcf and 4°C to extract the histones in the acid phase and isolate the supernatant. Cooled TCA was added to 21% final concentration and incubated for 2 h on ice. Samples were centrifuged for 5 min 16,000 rcf 4°C, and the pellet was then washed 4× with 100% acetone followed by air-drying of the remaining solution. Proteins were resolved on 4%–20% gradient SDS-PAGE gels (ServaGel TG Prime) and stained with Quick Coomassie Stain (Serva). Histone bands were cut out, and the gel pieces were destained 3× in a 50% ACN/100 mM NH_4_HCO_3_ solution, followed by 2× dH_2_O and 3× 100% ACN. Gel cubes were acylated by addition of 5 µl Propionic anhydride, 10 µl 100 mM NH_4_HCO_3_, and 35 µl 1 M NH_4_HCO_3_, respectively, followed by incubation at 37°C for 45 min and 750 rpm. Gel pieces were then washed 4× with 100 µl 100 mM NH_4_HCO_3_, 3× with 100 µl H_2_O, and 3× with 100 µl 100% ACN while completely drying out the cubes. Proteins were digested with 7.5 ng/µl trypsin solution in 50 mM NH_4_HCO_3_ with addition of H3.1 and H3.3 heavy amino acid standards (500 fmol, JPT Peptide Technologies) overnight at 37°C and 500 rpm. Peptides were collected in 100 µl 50% ACN/0.25% TFA followed by three washes with 40 µl 100% ACN, and the samples were passed through a C8 filtration tip (Affinisep) to ensure there were no remaining gel pieces. Peptides were dried and resuspended in LC loading buffer (PreOmics).

### 2.6 Total proteome from human plasma samples

Samples were prepared using a PreOmics iST kit from 1 µl of plasma based on the manufacturer's instructions.

### 2.7 LC-MS/MS acquisition and quantification

Peptides were injected in an Ultimate 3000 RSLCnano system (Thermo Fisher Scientific, San Jose, CA, USA) and separated on a 15 cm analytical column (75 μm ID with ReproSil-Pur C18-AQ 2.4 μm from Dr. Maisch) using a gradient from 4% B to 90% B (solvent A 0.1% FA in water, solvent B 80% ACN, 0.1% FA in water) over 90 min at a flow rate of 300 nl/min. The effluent from the HPLC was directly electro-sprayed into an Exploris 240 mass spectrometer (Thermo Fisher Scientific, San Jose, CA, USA). The mass spectrometer was operated in data-dependent mode to automatically switch between full scan MS and MS/MS acquisition. Survey full scan MS spectra (from m/z 375 to 1,600) were acquired with resolution *R* = 60,000 at m/z 400 (AGC target of 3 × 106). The 10 most intense peptide ions with charge states between 2 and 5 were sequentially isolated to a target value of 1 × 105 and fragmented at 27% normalized collision energy. Typical mass spectrometric conditions were as follows: spray voltage, 1.5 kV; no sheath and auxiliary gas flow; heated capillary temperature, 250°C; and ion selection threshold, 33,000 counts.

#### 2.7.1 Histone modifications

Raw files were analyzed using the Skyline software (29), as previously described (30)*.* Modifications with 10 or more missing values over paired samples were not considered. The log2-transformed normalized intensities were used for subsequent processing.

#### 2.7.2 Plasma proteome

One sample that displayed low identification rates was removed (Supplementary Material 2: Supplementary Figure S10). Using MaxQuant software (31), we identified 426 raw protein groups. After filtering out potential contaminants and reverse matches, we retained 380 protein groups with at least one MS/MS identification across samples and one iBAQ (intensity-based absolute quantification) intensity value >0 across samples, resulting in 342 protein groups. The number of missing values per sample ranged from 47 to 148, with a median of 55 (8 samples, 4 BT and 4 AT), 104 (16 samples, 11 BT and 5 AT), and 96 (6 samples, 4 BT and 2 AT) for batches 1, 2, and 3 respectively. Then, protein groups identified using a match between runs in nine or more paired participants at both BT and AT (80% of the paired participants) were retained, resulting in a total of 324 protein groups corresponding to 454 proteins.

### 2.8 LC-MS/MS data processing

For each dataset (buccal cells, PBMCs, plasma), after quality control and quantile normalization (Supplementary Material 2: Supplementary Figures S6, S10, S11), we imputed missing intensities differently in case of not missing at random (NMAR) or missing at random (MAR), if the number of missing values per condition, i.e., BT or AT, is higher or lower than 75%, respectively. For NMAR values, missing values were sampled from a Gaussian distribution in which the average is set to the average intensity over all intensities lower than the first percentile and standard deviation is set to the median standard deviation of all intensities across all samples. For MAR values, the missing intensities were sampled from a Gaussian distribution with an average set to the average intensities over the condition for the non-missing values of that protein group with a standard deviation of 0.5, all on the log2-scale. We used an empirical Bayesian framework to adjust intensities for batch effect using the sva::*ComBat* R function (32). The relative abundance (percentage) of each modified peptide was then computed for a given precursor (buccal cells, PBMCs datasets), and we used the log2-intensities as protein group abundance (plasma dataset).

### 2.9 Statistical analyses of PTM and plasma biomarkers of sport therapy

#### 2.9.1 Biomarker discovery

**Figure 4:**
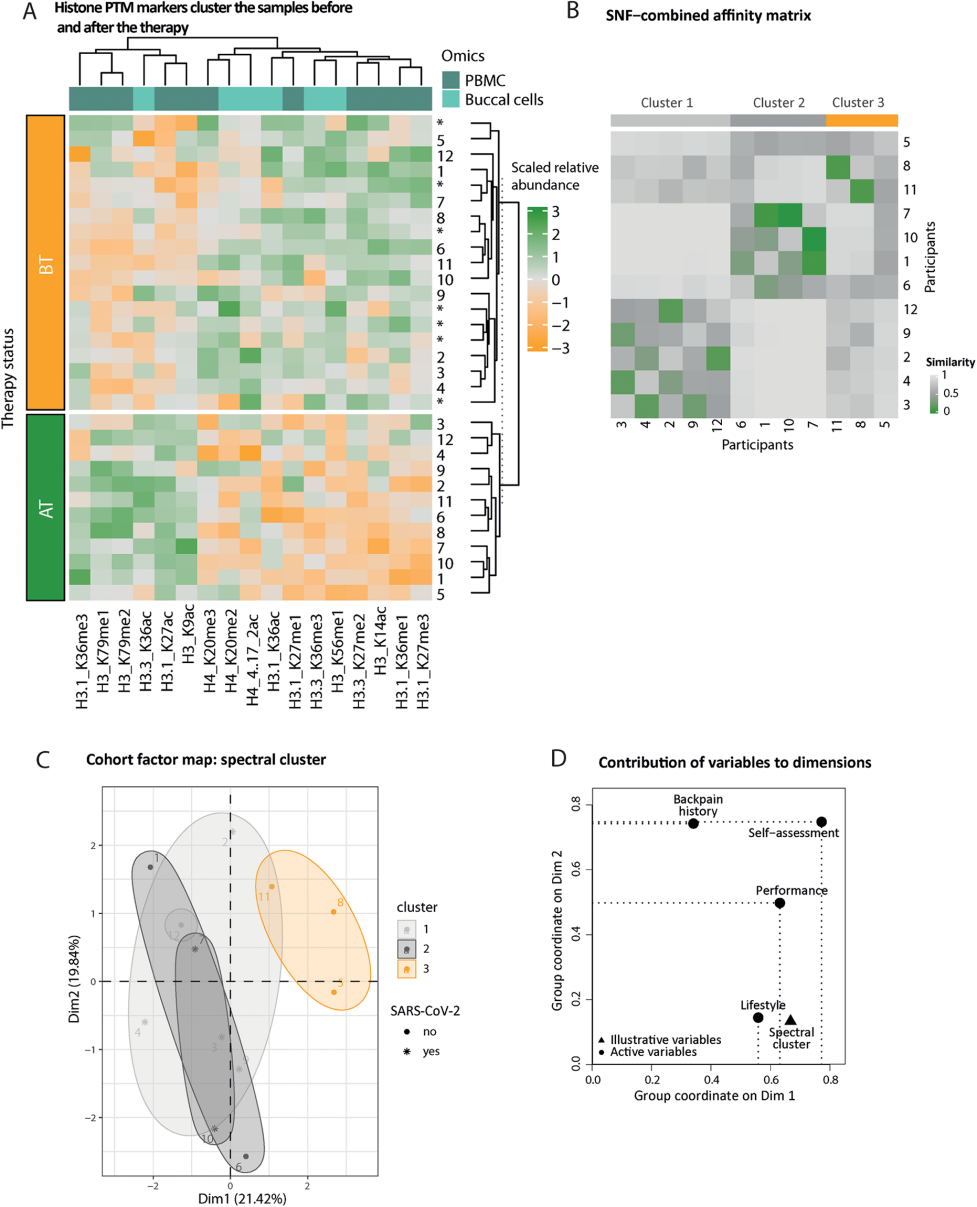
Histone epigenetic markers’ distribution reflects the therapy status of the participants. **(A)** Heatmap of scaled relative abundances of histone posttranslational modifications (PTMs). Hierarchical clustering on rows (samples, unpaired annotated with an asterisk) and columns (histone PTMs) reflects the status of the participant, before (BT, orange) or after (AT, green) therapy. The origin of the markers is color-coded on top of the columns (from PBMCs dark turquoise and from swabs light turquoise). **(B)** Fused pairwise participants affinity matrix elucidated complementary information from both swab and PBMC epigenetic affinity matrices using the similarity network fusion (SNF) algorithm. The similarity matrix diagonal has been set to the median value of the entire matrix. The color of the cell indicates increased similarity, from gray to green. Spectral clusters’ belonging is indicated in gray or orange shade. **(C)** Participant factor maps from MFA performed from (1) performance, (2) self-assessment, (3) lifestyle, and (4) back pain history groups of variables using spectral clusters class as illustrative variables (color-coded). COVID-19 infection during the therapy period is indicated as an asterisk. **(D)** The coordinates of the variable groups illustrate correlation with the first two MFA dimensions; top (i.e., above uniform) active (round-shaped) contributors are represented by the dotted lines per dimension.

**Figure 5:**
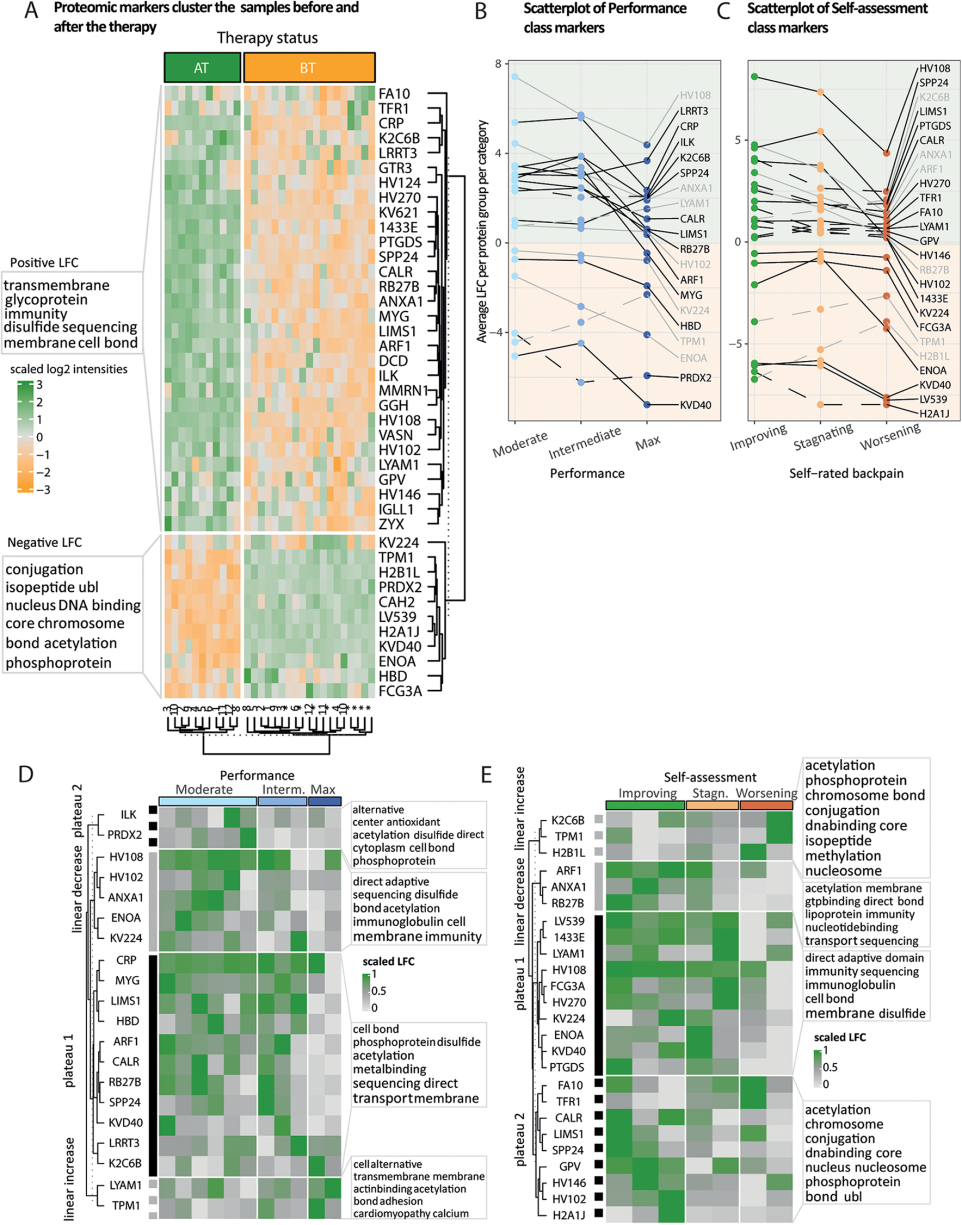
Fold change patterns of protein markers accurately predict therapy status and therapy response. **(A)** Heatmap of scaled proteomic markers’ abundances identified in the plasma. We performed hierarchical clustering on the rows and columns. The rows are labeled by the leading protein of the protein group, and the columns indicate the status of the samples before (BT) and after (AT) the therapy. Participants annotated with an asterisk submitted only BT samples. Unsupervised clustering reflects the status of the samples. The two clusters of proteins with either an average positive or a negative log2-fold change (LFC) are annotated with the 10 most representative UniProt keywords. **(B,C)** Per marker, we computed the average LFC (*y*-axis) over physical performance or self-assessed therapy effect categories (*x*-axis), respectively. After having clustered markers’ LFC based on their shape, a subset of markers displays a linear trend (increasing, dashed gray or decreasing, full gray line) or a plateau (first two categories, full black line or last two categories, dashed black line). The light orange and green backgrounds indicate the sign of the LFC, negative and positive, respectively. **(D,E)** Heatmap of scaled LFC per participant (columns) ordered by physical performance or self-rated back pain categories, using the subset of markers defined in **(B,C)**, respectively. Per marker, LFC values have been centered using either the mean over categories (linear trends) or the plateau mean (plateaus), normalized between 0 (gray) and 1 (green).

For each of the three datasets (buccal cells, PBMCs, and plasma), we searched for candidate markers by intersecting the outcomes of both classic tests (paired Wilcoxon and *t*-tests) and a supervised machine learning algorithm [partial-least square discriminant analysis or PLS-DA framework (33, 34)] applied on the changes of molecular profiles AT relative to BT, at *α* = 0.05 threshold after multiple testing correction, and a variable importance score (VIP) value of 1 (from the lower bound of Jackknife 95% confidence interval). Volcano plots and VIP distributions are represented in Supplementary Material 2 (Supplementary Figures S7, S12). This led to 12 (+1 precursor) swab and PBMC epigenetic markers (Figure 4A), and 41 protein therapy markers and 6 (+2 precursors) (Figure 5A), respectively. We computed the average log2-fold change (LFC, plasma proteins) or average percentage of change (PTMs) after-vs-before therapy over 11 and 12 paired datasets as a final estimate of each marker's effect size. Analytical procedures have been additionally described in Supplementary Material 1 (Supplementary Methods).

#### 2.9.2 Histone PTM analysis

We performed an unsupervised hierarchical clustering of the relative abundances of the PTM markers from paired or unpaired samples (the latter did not contribute to the identification of the markers) to check if the clustering of the samples reflects the therapy status (BT or AT). Since swab and PBMC markers do not overlap or cluster by sample type (Figure 4A), we assessed the amount of complementary information among these datasets by using the similarity network fusion algorithm to detect (latent) groups of individuals (Figure 4B, Supplementary Material 2: Supplementary Figure S8). To interpret the resulting three clusters, we performed an MFA using the spectral clusters as illustrative variables and the following sets of variables as active qualitative variables: (1) lifestyle (exercise and dietary habits classes, two variables), (2) back pain history (last 12 months, last 3 months and at entry, three variables), and (3) therapy evaluation (performance and self-assessment classes) (Figures 4C,D). Intermediate plots useful for the axes' interpretation as well as individual factor map are represented in Supplementary Material 2 (Supplementary Figure S9).

#### 2.9.3 Plasma proteome analysis

Similarly, as for the epigenetic markers, we performed an unsupervised hierarchical clustering on plasma marker intensities (Figure 5A). Both clusters of negative and positive LFCs were then labeled with the 10 most representative Uniprot keywords using world cloud representations. We zoomed on the subsets of markers whose intensities displayed either a linear trend or a plateau over the three performance categories and the three back pain self-assessment categories pertinent for the therapy evaluation (“Improving,” “stagnating,” and “worsening”) (Figures 5B–E).

### 2.10 Data integration of PTM and plasma biomarkers

**Figure 6:**
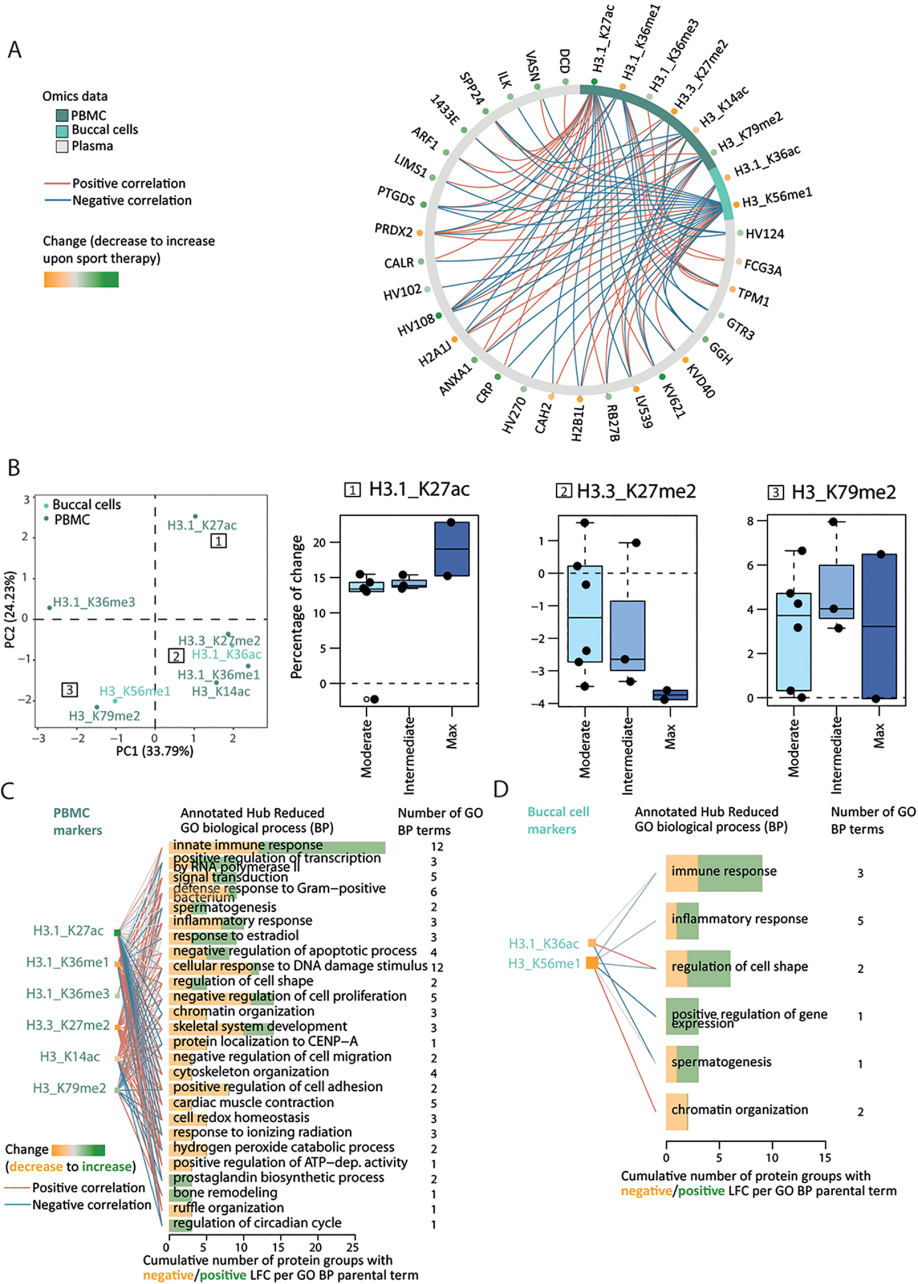
Epigenetic–proteome interactions reflect multilevel changes upon therapy. **(A)** Data Integration Analysis for Biomarker discovery using Latent variable approaches for Omics studies (DIABLO) maximizes the correlation between changes in epigenetic (swab and PBMC) and proteomic markers upon therapy. The correlation networks represent strong (above 0.7 absolute value) positive (red) and negative (blue) correlation between epigenetic (2 swab markers, 6 PBMC markers) and proteomic (27 markers) datasets. The LFC/percentage of change of each proteomic/epigenetic marker is encoded by a green-to-orange dot, from increase to decrease upon therapy. **(B)** Principal component analysis (PCA) 2D map from the percentage of change of the above eight epigenetic markers. The 2D map quadrants represent different PTM effect size trends over back pain self-assessment categories [see example inset boxplots; (1) plateau-like with the highest percentage of change, (2) pseudo-linear, or (3) complex inverted U-shape]. **(C,D)** GO biological process (BP) annotation of the 27 protein markers highlighted by the functional DIABLO analysis separately for PBMC **(C)** and buccal cell **(D)** markers. GO terms whose annotated GO BP terms match 3 or more connexions were considered and only reduced GO BP terms are represented (*y*-axis) and ranked by their corresponding number of annotated protein groups (*x*-axis) and by the GO hierarchy.

We compared the clustering of individuals across the samples from the list of markers displayed in Figure 4A (PTMs) and Figure 5A (proteome) using the dendextend R package [version 1.17.1, (35)]. We represented pairwise distance differences using a sorted tanglegram with its associated cophenetic correlation value (Supplementary Material 2: Supplementary Figure S13). Furthermore, since histone modifications can act as repressive or activating marks, we aimed to identify correlated signatures of therapy response across different sources of biological samples using Data Integration Analysis for Biomarker discovery using Latent variable approaches for Omics studies (DIABLO, Figure 6A) (36). To get a better understanding of the top (using a correlation threshold of 0.7) PTM–plasma protein interactions, we annotated the 27 protein group markers connected at least once with any of the six PBMC- and two swab-originating PTMs with GO biological process (BP) terms using the UniProt database. Annotations were represented at the parental term level in Figures 6C,D (detailed in Supplementary Material 2: Supplementary Figure S15). Because these eight PTM changes correlated with protein changes involved in biological processes, we additionally investigated the distribution of their percentage of change upon therapy over performance and self-assessment categories (Supplementary Material 2: Supplementary Figure S14); different trends located in different quadrants of the principal component analysis (PCA) 2D plane over categories were noticed (Figure 6B). All steps are described in Supplementary Material 1.

### 2.11 Effects of therapy on health awareness indicators across biological samples

**Figure 7:**
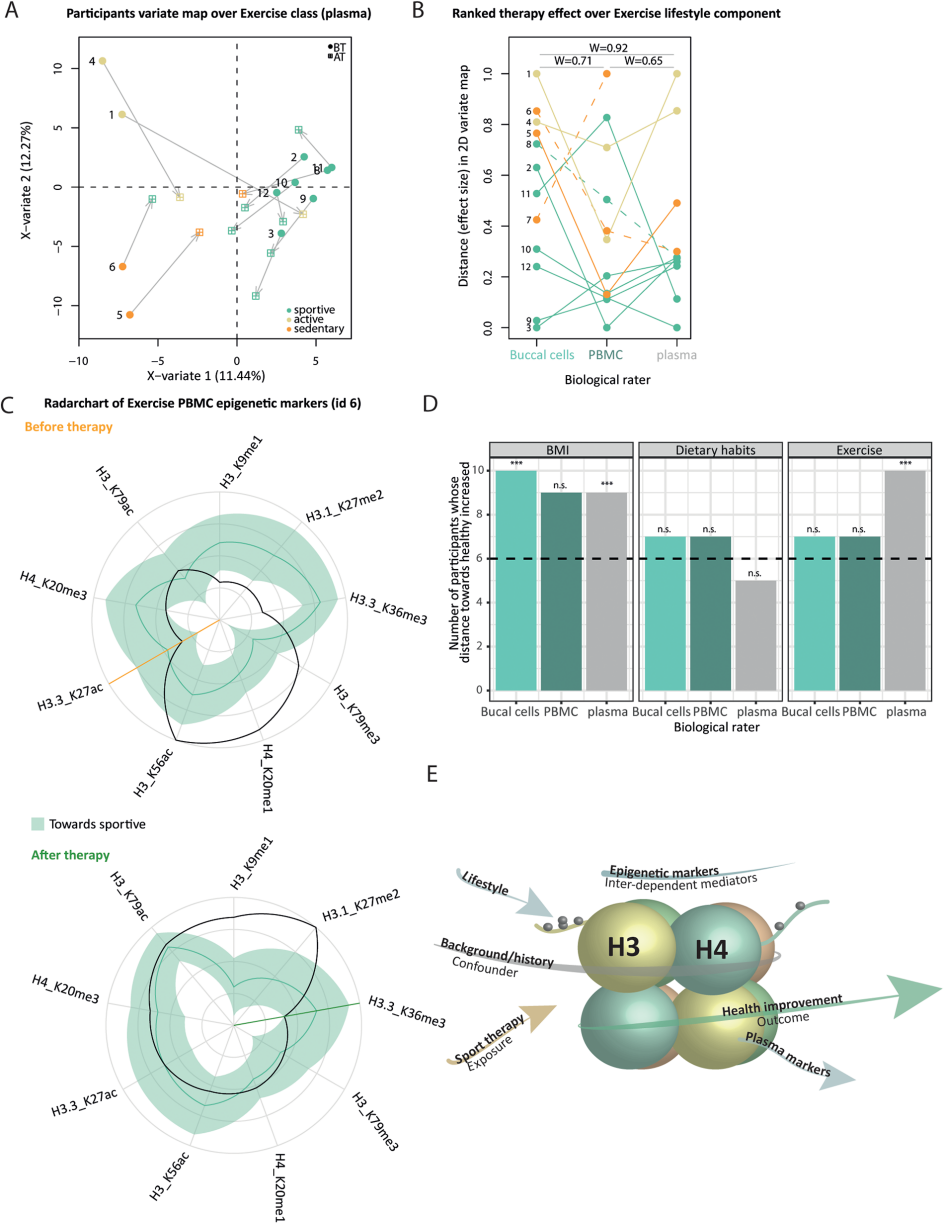
Health awareness indicators change consistently upon therapy across biological samples but reflect cohort variability. **(A)** 2D-variate PLS-DA map from BT plasma profiles using exercise categories as outcome (color-code in the legend). Participant consists of two points: initial BT position (round-shaped) and AT position (square-shaped) after the projection of the AT profile in the map, connected by an arrow. **(B)** Bump chart representing the participants’ ranks (*y*-axis) from increased therapy effect size [i.e., arrow length in **(A)**] across sample types (*x*-axis) from exercise categories. Connecting lines are colored from each participant's BT category. The dashed line indicates that the participant switched categories upon therapy. Pairwise concordance Kendall's values are reported across biological raters. Overall, the test reports a marginally significant difference across raters [*W* = 0.68, *χ*^2^ (10) = 0.5, *p* = 2.50 × 10^−2^]. **(C)** Radar chart from participant ID 12 PBMC datasets BT (left) and AT (right) from exercise class markers (*x*-axis). Clockwise, we ordered health awareness indicators from decreasing distance (normalized markers values represented as a black line, *y*-axis) to the healthy range (green ribbon, median as a plain line). The orange/green line indicates the point when indicators display discrepancy with the healthy range, BT and AT, respectively. The more within the upper right quadrant, the more similar the profile is to the healthier category of participants. **(D)** Per biological sample (*x*-axis) and lifestyle component (facet), the bar chart represents the number of participants (*y*-axis) whose number of markers within the healthy range increased upon therapy. *P*-values of paired *t*-tests from weighted similarity to healthy range BT and AT after Benjamini–Hochberg correction are encoded as follows: “n.s.” and “*,” “**,” “***,” and *α*, respectively, set to >0.1, 0.1, 0.05, and 0.01. **(E)** Conceptual representation of (in)direct association between therapy attendance (orange arrow) and health outcome (green), mediated by lifestyle, and correlated changes of epigenetic and proteomic (light blue). The individual background (gray) may introduce confounding at any level.

Per sample type (buccal cells, PBMC, plasma) and for three lifestyle components [exercise, dietary habits classes, and BMI – obese/overweight (BMI) > 29.9 or normal BMI = 24.9–29.9], we searched for discriminative markers of categories from the full epigenetic/proteomic profiles using the PLS-DA framework. This led to a total of 3 (sample type) × 3 (lifestyle) × 2 (BT/AT) PLS-DA models. From the predicted AT position in the 2D PLS-DA variate map (Figure 7A, Supplementary Material 2: Supplementary Figure S16), we computed the Euclidian distance between BT and AT points for a given participant, denoted by d_BT−AT_, where each PTM/protein has a weighted contribution to the therapy effect on a given lifestyle component. Per lifestyle component, we obtained three d_BT−AT_ vectors (one per sample type, Supplementary Material 2: Supplementary Figure S17) whose pairwise concordance between sample types is quantified by Kendall's W (Figure 7B, Supplementary Material 2: Supplementary Figure S19) and represented against the BT distance to the gravity center of the healthy categories using robust regression (Supplementary Material 2: Supplementary Figure S18). We relied on the clustering obtained from the questionnaire data to define healthy categories of individuals before and after therapy; sportive (exercise class), flexitarian/balanced diet (dietary habits class), and normal BMI categories. Because d_BT−AT_ is not oriented, i.e., does not indicate if the effect size reflects a change toward healthy or unhealthy state upon therapy, we computed a personalized weighted similarity metric to the healthiest categories of participants before and after therapy, using the normalized values of VIP-based health awareness indicators and their distance to the healthy 95% Jackknife-based range (Figure 7C, Supplementary Material 2: Supplementary Figure S20). We assessed the presence of any linear relationship between d_BT−AT_ and similarity to the healthy optimum at BT and AT using robust linear regression (Supplementary Material 2: Supplementary Figure S21). We performed paired *t*-tests, BT and AT, on the healthy similarity metrics and reported the Benjamini–Hochberg adjusted *p*-values (Figure 7D). All procedures are detailed in Supplementary Material 1.

## 3 Results

### 3.1 Study design

In this study, 12 young men completed a curative back pain training program comprising 24 sessions (Supplementary Material 1: Supplementary Methods, Supplementary Table S1). For each participant, a questionnaire detailing back pain history and intensity, nutritional choices, exercise habits, and agility metrics was collected as well as buccal swab and venous blood samples at the beginning and at the end of the therapy. We aimed to investigate epigenetic profiles in easily accessible samples and systemic plasma proteome changes upon therapy using mass spectrometry (Figure 1). Information obtained from the questionnaire elucidated the impact of lifestyle on the therapy outcome.

### 3.2 Lifestyle profiling upon therapy

To learn about the cohort's homogeneity and to evaluate the association between the participants’ background and their response to therapy, we established categories of participants that share similar patterns of answers to the questionnaire. We created six groups of information to get a more objective overview from subjective questionnaire information (Supplementary Material 1: Supplementary Methods, Supplementary Tables S2–S8): dietary habits, exercise, back pain status, agility metrics before and after the therapy (BT and AT), and long- (1 year) and short-term (3 months) back pain history. For each class, we established typical profiles (described in Figure 2A legend). The lifestyle sequence plot (Figure 2A) indicates that all 12 participants display different, but sometimes common habits and histories contrasted by healthiness. As expected, the cohort is lacking extremely unhealthy participants (Figure 2A), potentially due to compensating mechanisms (e.g., participant 4 has a poor diet but is sportive) and the restricted inclusion criteria (e.g., only young participants were admitted). Although collected (Supplementary Material 1: Supplementary Table S9), sociodemographic information was not used to extract categories due to the lack of variability in the data (e.g., one smoker, one person with children) or incomplete information (e.g., work type). Back pain history trajectories differ among participants over time, from mild to fluctuating, chronic, and even worsening pain (Figure 2B), with almost half of the participants starting the therapy with frequent acute to chronic back pain (Figure 2A). We reported a pairwise correlation between the categories to investigate the dependency of lifestyle components (Figure 2C). We noticed stereotypical behaviors, e.g., unhealthy nutritional choices are associated with a higher sedentary lifestyle. Negative correlations of back pain variables with exercise and nutritional choices indicate that a healthy lifestyle might prevent back pain (37, 38). Long-term back pain history (>1 year) seems a good candidate predictor of back pain at 3 months, as well as at the entry of the therapy [before therapy (BT)] for most of the participants, reflected by strong positive correlations (Figure 2C).

### 3.3 Sport therapy leads to a spectrum of objective and self-rated improvements

Primary outcome variables from the 12 participants who completed the study included improvement in (1) agility metrics (flexion, extension, and rotation), (2) self-rated back pain, and (3) self-rated well-being. Because participants have different physical characteristics (e.g., size), the objective (i.e., not self-rated) quantitative variables were adjusted for background baseline levels to evaluate changes in the agility metrics (Supplementary Material 1: Supplementary Methods). We used the Gaussian mixture modeling framework (39) to investigate the presence of sub-groups of participants according to changes in agility metric upon therapy (Supplementary Material 1: Supplementary Tables S10, S11). All the participants responded positively to the therapy but to different extents: with moderate, intermediate, or max performance (Figure 3A), defining the so-called performance response class (classes will subsequently be indicated with a capital letter). The participants completed between 16 and 24 sessions in a period of 4–6.4 months, which led to a heterogeneous therapy response. On average, participants attending more sessions per week tend to display higher improvement in agility measurements after the therapy (Figure 3B). The changes in self-rated back pain status, termed self-assessment class, indicate different degrees of improvement in back pain frequency upon therapy (Figure 3C). More specifically, four participants reported back pain improvement, out of which one participant switched from acute/frequent back pain to no/moderate back pain category, while two participants reported back pain worsening after the therapy (AT) (Figure 3C, individual alluvia). Surprisingly, we did not find a significant association between performance and back pain self-assessment classes; individuals categorized as max performers identified their back pain journey as worsening or stagnating, while intermediate responders reported improvement or no back pain (Figure 2D). This may be explained by an excess of exercise of the max performers. While an increased number of weekly sessions seems to be biased toward higher improvement in agility metrics (Figure 3B), three out of four participants with less than 1 weekly session on average and therefore longer participation in therapy reported back pain improvement (Figure 3E).

Despite one-third of participants who entered the study with substantial back pain reported worsening or stagnating back pain after therapy, we found significant differences in self-rated well-being and physical capacity scores with a median gain of 1 point for each score upon therapy. Gains distributions are represented by a sunburst plot (Figure 3F). Although we lacked participants to perform stratified analyses, we noticed bigger gains for participants with no/minor back pain and participants whose back pain improved for both scores and three and five participants whose physical capacity and well-being scores did not change (Figure 3F).

To evaluate the factors that may influence performance and self-assessment response classes, we performed a multiple factor analysis (MFA) (Supplementary Material 2: Supplementary Figures S4A,B). The 2D factor map of participants with substantial back pain at the start reveals that the dimensions (Dim) are driven by extreme participants in terms of performance, while the back pain self-assessment naturally splits participants according to worsening and improving scenarios. This can predominantly be explained by the strong contribution of the sociodemographic environment and back pain history on Dim1 and lifestyle on Dim2 (Supplementary Material 2: Supplementary Figure S4C). An increasing positive coordinate along Dim1 matches increased back pain, more often associated with work, whereas the second dimension reflects a gradient of a less healthy lifestyle, i.e., toward undiversified diet and sedentary habits with increasing positive coordinate (Supplementary Material 2: Supplementary Figure S4C, S5). Since this study was carried out during the SARS-CoV-2 pandemic, it has likely influenced the duration of the therapy (5-month span on average) and potentially the molecular response of the participants. Dim1 indeed reflects the clustering of participants who reported COVID-19 infection on Dim1; however, this does not seem confounded with performance or self-assessment categories (Supplementary Material 1: Supplementary Figures S4A,B).

### 3.4 Epigenetic markers elucidate participants' therapy status

We obtained a reliable set of sport therapy epigenetic markers from buccal cells and PBMCs, i.e., 6 and 11 markers, respectively. Unsupervised clustering of epigenetic profiles restricted to sport therapy markers confirms that samples obtained before (BT) or after (AT) the therapy accurately reflect the therapy status (Figure 4A, rows). Additionally, unpaired BT samples of participants who did not complete the study and did not contribute to the markers' identification cluster with their respective sport therapy status (Figure 4A, rows, stars). We observed a predominant decrease in histone methylation after the therapy (Figure 4A, columns). We hypothesized that both sample types (buccal swabs and PBMCs) bring unique, but complementary information on the therapy effect on molecular profiles since buccal cell and PBMC therapy markers do not overlap. We thus combined the participants' epigenetic response conveyed by both buccal cells and PBMCs with the similarity network fusion method (SNF, Supplementary Material 2: Supplementary Figure S8) (40). The fused similarity matrix suggests distinct clusters of participants (Figure 4B). We performed an MFA to find the drivers of shared epigenetic response among subsets of participants. In the individual 2D factor map (Figure 4C), the first dimension—displaying the strongest correlation with the cluster illustrative variable—separates cluster 3 (orange) from the other two. One explanation is the strong contribution of self-assessment and performance classes (Figure 4D), two out of three participants are the only ones identified as worsening back pain and max performers (Figures 4C,D). An increasing positive coordinate along the first dimension of the factor map additionally matches a less healthy lifestyle, i.e., toward undiversified diet and sedentary habits. The second dimension reflects a gradient of increased back pain with increasing positive coordinates (Figures 4C,D, Supplementary Material 2: Supplementary Figure S9).

### 3.5 Sport therapy benefits are reflected in plasma proteome changes

Plasma proteome analysis offers a detailed portrayal of a person's general health status and is especially effective in monitoring patients throughout therapeutic interventions (41). Similarly, as for defining histone posttranslational modification (PTM) markers (Supplementary Material 1: Methods), we established a set of 41 sport therapy plasma markers that accurately reflect the therapy status using unsupervised hierarchical clustering (Figure 5A, columns). Distinct profiles of protein markers divided the samples according to their before (BT) or after (AT) therapy status. Additionally, the four individuals who did not complete the therapy clustered with the paired BT samples (Figure 5A, rows). Overall, we observed a subset of immunoglobulins more abundant after the therapy, which indicates the influence of the therapy on the immune response [Figure 5A, positive log2-fold change (LFC)]. Following epigenetic adaptations, we aimed to investigate how downstream molecular information (plasma molecular layer) reflects objective (agility performance) and subjective (back pain self-assessment) evaluation of the participants' well-being journey. We assumed that changes in protein markers may reflect physiological changes that match the categories. Screening the average LFC of protein markers revealed distinct trends between different categories: a linear trend (gray trajectories) or a plateauing trend (black trajectories) across the three ordinal categories of each response class (Figures 5B,C). Participants with no/minor back pain (unimpaired) during the study were excluded from the trend detection since we do not expect them to display large proteomic perturbations upon therapy. In total, 20 and 25 protein markers displayed trends over performance and self-assessment response categories, respectively (Figures 5B,C). Because the retained trends were evaluated from average LFC values, we represented and annotated the individual LFCs per trend and response category (Figures 5D,E). Overall, the LFC distributions reflect a substantial amount of variability in effect size among participants even within the same response category. Moderate and intermediate performers displayed a strong and consistent increase of immune response proteins (negative linear trend: HV108, HV102, ANXA1) and proteins involved in oxygen transport (42–46) (plateau 1: increased MYG levels and decreased HBD levels), compared to max performers. TPM1 protein, involved in striated muscle contraction, decreased upon therapy (47); this indicates a potential benefit of the therapy as an increase in TPM1 levels has been identified as a systemic pro-aging factor in mouse retina and young mouse models of Alzheimer's disease (48). With overall positive LFCs, we observed a joint linear decrease over the self-assessment categories of the anti-inflammatory mediator ANXA1 and the small GTPases ARF1 and RB27B (Figure 5E, clusters) involved in trafficking between an elaborate network of subcellular compartments, cell growth, and differentiation (42, 49, 50). Furthermore, participants reporting stagnating or worsening back pain displayed lower positive increase (HV270, HV146, HV102) or higher negative decrease (KV224, KVD40) of immunoglobulins involved in immune response and complement system activation (44, 51). Additionally, participants with worsening back pain displayed on average bigger decrease of ENOA, reported to stimulate immunoglobulin production, glycolysis, and hypoxia tolerance, and lower increase of PTGDS, a prostaglandin involved in contraction or relaxation of smooth muscle and maintenance of the central nervous system and male reproductive system (Figures 5D,E) (52–56).

### 3.6 Data integration reveals that H3.1 K27ac and H3 K56me1 changes display predominantly opposing correlation with plasma biomarkers

Histone modifications can act as activating or repressive marks involved in altering chromatin structure and consequently gene transcription and protein expression. This in turn modulates a plethora of biological processes (57). We aimed to describe the co-occurrence of these dynamic adaptations on different biological levels as a response to sport therapy. We first checked if the differences between participants' omic profiles are preserved using the epigenetic markers or the plasma markers before and after therapy (Supplementary Material 2: Supplementary Figure S13). Our high cophenetic correlation value (0.87) indicates that, although PTMs and proteins have different dynamics, inter-participant distances are real and preserved using either omic type. We focused on high-degree PTMs, originating from both buccal cells and PBMCs, that display changes strongly correlated (threshold of 0.7) with changes in plasma protein therapy markers and visualized the corresponding correlation network (Figure 6A). Among the eight high-degree PTMs, except for H3 K14ac and H3.1 K36ac, a positive fold change in histone acetylation was observed for H3.1 K27ac and a predominantly negative fold change in methylation (H3.1 K36me1, H3.3 K27me2, H3 K56 me1).

An increase in histone acetylation and a decrease in methylation are associated with an overall increase of many protein markers (19/27 positive LFCs; Figure 6A, dots on gray circle). We, therefore, assumed that the distribution of their changes is not arbitrary and reflects therapy response (performance and self-assessment categories). We thus performed a principal component analysis (PCA) from the individual percentage of change of these eight PTMs. The location of the PTMs within the 2D-PC plane hints at three, arguably weak, trends described as insets in Figure 6B: (1) plateau-like with the highest percentage of change, (2) pseudo-linear, or (3) inverted U-shape. Observed trends are comparable for performance and self-assessment categories (Figure 6B, Supplementary Material 2: Supplementary Figure S14). To get biological insights into the PTM–plasma protein interactions, we supplemented the correlation network of Figure 6A with Gene Ontology biological processes (GO BP) from the UniProt database (58). Thus, one PTM previously connected to one or more protein group(s) is now connected to its/their corresponding GO annotation. Because of different sample origins, we have done this separately for the six PBMC markers (Figure 6C) and the two buccal cell markers (Figure 6D). The innate immune response is among the most common signaling terms, as expected when using plasma as a biological sample (59). Positive and negative LFCs (green and orange bar length) suggest concerted, but complex action of H3.1 K27ac (positive LFC) and H3.3 K27me2 (negative LFC) in PBMCs (Figure 6C). Interestingly, although PBMC and buccal cell markers do not overlap, they converge into similar GO BP profiles. Overall, the complex change of the epigenetic and plasma protein markers influenced by the therapy resulted in modifications of immune response (innate immune system, defense response to bacterium), signal transduction and gene regulation, and changes in skeletal and muscle proteome (Figures 6C,D, Supplementary Material 2: Supplementary Figure S15).

### 3.7 Inter- and intra-variability of molecular profile depends on lifestyle and is associated with a spectrum of therapy effect size

Although we screened for sport therapy markers, the therapy may globally affect molecular profiles in a way that depends on participants' backgrounds and habits. This is expected as lifestyle components (here BMI, dietary habits, and exercise) encompass many complex traits that vary in the general population. We performed an exploratory analysis to understand to what extent the therapy affects histone PTM and plasma proteome changes associated with either BMI, dietary habits, or exercise—those markers are subsequently termed health awareness indicators. Overall, the 2D-variate maps reflect lifestyle categories before therapy (Figure 7A, Supplementary Material 2: Supplementary Figures S16, S17), indicating that molecular profiles share enough common patterns (inter-category variability) while still displaying distinct features (intra-category variability).

Euclidian distance between BT and predicted AT points served as a proxy of the distributed therapy effect on health awareness indicators per participant (Figure 7A, represented by arrows). For each combination of lifestyle components (BMI, nutrition, exercise) and sample types (buccal cells, PBMCs, plasma), all participants diverge from their initial BT state, and their corresponding arrows may sometimes converge in the same direction (Figure 7A). This suggests that participants' profiles become more similar upon therapy constraints. However, this is not a systematic pattern: some participants converge more than others to a healthier state upon therapy (Supplementary Material 2: Supplementary Figure S16), rather than becoming more similar to the average. To understand this heterogeneity, we performed robust linear regression of the therapy effect, with the initial (BT) distance to the healthy optimum (Supplementary Material 2: Supplementary Figure S18). We found overall significant positive associations for buccal cells independently of the lifestyle component—the more distant from the healthy optimum at the start, the bigger the therapy effect. This is in line with (1) a bigger therapy effect for participants switching toward a healthier lifestyle category upon therapy (Figure 7A, ID 8 and 6; Figure 7B, dashed line) and (2) a trend of smaller therapy effect for participants belonging to healthy categories at the start (Supplementary Material 2: Supplementary Figure S18).

Rating participants by their respective Euclidian distance (Figure 7B) summarizes the variability of the changes in health awareness indicators within the cohort, although self-report-based lifestyle categories did not change significantly upon therapy (Supplementary Material 2: Supplementary Figures S1, S2). Participants' rankings were relatively agreeing across sample types for the exercise class (Figure 7B, Kendall's W >0.65, with marginal significance observed) but fluctuated for dietary habits and BMI (Supplementary Material 2: Supplementary Figure S19, Kendall's W from 0.14 to 0.71) whose ranks are less concordant across sample types. Since the aim of the sport therapy was to introduce targeted exercise, it was therefore not expected to observe coherent changes in dietary habits and BMI. Overall, this indicates that the therapy effect on health awareness indicators diverges across sample types. Lifestyle impacts molecular profiles from different origins differently, which likely depends on constrained functional relationships (overview in Figures 6A–D), and/or various degrees of plasticity (60). Such changes can be effectively evaluated from easily available samples such as buccal swabs, and it might not be necessary to resort to direct and invasive sampling methods such as tissue biopsy.

Nevertheless, the Euclidian distance is not oriented, and it does not indicate if the effect size reflects a change toward a healthier category upon therapy. To complement this, we measured the similarity of a given participant's profile to the average healthiest group before and after therapy, using AT normalized values as a reference. For each lifestyle component and sample type (Supplementary Material 2: Supplementary Figure S20), we recorded the VIP-based health awareness indicators (Figure 7C, radar chart *x*-axis) whose normalized values fall within the Jackknife healthy range (Figure 7C, black line vs. green ribbon) before and after therapy. Because PTM/protein measurements are variable, we additionally computed a weighted averaged similarity score that considers the distance of each marker to the healthy range. Independently of lifestyle component and sample type, more than half of the participants display higher similarity to healthy profile upon therapy (Figure 7D, bar chart height), with significantly higher similarity scores to healthy categories for some sample types (Figure 7D, one-sided paired *t*-tests). For exercise, BT and AT distance to healthy negatively correlate with the Euclidian distance (Supplementary Material 2: Supplementary Figure S21, significant Wald tests) independently of the sample type, confirming again that the therapy effect goes in the direction of healthier status.

Although participants converge on average toward a healthier state, there is a substantial amount of heterogeneity within the cohort, which may partly be due to differences in individual backgrounds (Figure 7E, gray arrow). The confounding effect of the background introduces difficulties in reconstructing the causal path between therapy exposure and therapy outcome (Figure 7E, conceptual diagram). We reported distinct therapy effects on health awareness indicators across biological samples (Figure 7B, Supplementary Material 2: Supplementary Figure S19), suggesting that the hierarchical nature of the molecular data (Figure 7E, light blue arrows) and its nested contribution to the lifestyle categories (Figure 7A, and Supplementary Material 2: Supplementary Figure S16) might dilute the therapy effect. The therapy effect (Figure 7E, orange arrow) is likely mediated by many (intermediate) molecular phenotypes (Figure 7E, light blue arrows). Using univariate analysis over lifestyle components, we noted that the more distant from the healthy optimum at the start, the bigger the molecular therapy effect (Supplementary Material 2: Supplementary Figure S18), suggesting that lifestyle is also a mediator of the therapy effect (Figure 7E, light blue arrow). Surprisingly, despite non-significant BMI changes, we found a tendency toward healthier values of BMI health awareness indicators (Figure 7D), which might reflect the potential of molecular markers to illustrate more subtle health changes between the BMI categories.

## 4 Discussion

Epigenetic modifications are highly dynamic and responsive to various lifestyle influences, such as nutrition or exercise. They are already used as diagnostic indicators and predictors of therapy outcomes (61). Therefore, we aimed to find out whether epigenetic response can reflect complex, even subjective lifestyle traits and therapy outcomes in an objective and quantifiable manner. To fully grasp the epigenetic potential to illustrate a person's well-being journey as the complex interplay of their (1) background, (2) standardized back pain therapy, and (3) intractable lifestyle influences, we used several levels of information. We collected a palette of standard therapy basal information (e.g., height, weight, age) and medical history, followed by a set of lifestyle questions, agility measurements, and plasma proteome changes as a downstream molecular response to the epigenetic adaptations.

Back pain is a worldwide health concern that occurs in every age, gender, or income group. It is a leading cause of disability and a reason for work absence, with a very heterogenous pathological cause (5, 62). In this context, dissecting the contribution of the social and environmental exposome on composite epigenetic biomarkers can provide mechanistic insights into back pain pathogenesis and therapy approaches. In this pilot study on young male participants, lifestyle categories and back pain history were defined from non-standardized questionnaires, which may be subject to recall bias or imprecision. To mitigate this bias, we built informative categories from different answers and confirmed that participants have stereotypical behaviors where, for example, bad eating habits are associated with a more sedentary lifestyle. To fully understand the information contained in participants' epigenetic profiles, we also used objective measurements of therapy effect and health status (agility metrics and plasma proteome). This revealed that objective agility gain (performance) is surprisingly inversely connected to how one perceives his own back pain improvement (self-assessment). This suggests that there is a threshold in agility gain above which it may negatively influence back pain therapy outcomes. It also raises caution regarding the ability of biomarkers to show a global picture of the biological processes involved in back pain therapy, since significant fold change can also imply adverse therapy effects. Further studies are necessary to understand whether adverse effects are neutralized by beneficial effects during exercise. To conduct a comprehensive analysis, we utilized both unsupervised (SNF-based spectral clustering, hierarchical clustering comparison) and supervised (N-integration discriminant analysis with DIABLO) integrative approaches to reveal correlated changes across different sources of information and complement the understanding of histone epigenetic adaptations in the context of back pain therapy, which has not been done before.

The impact of exercise regime on epigenetic modifications in humans was reported for local changes in the skeletal muscle using tissue biopsy and often using targeted antibody-based approaches (22, 23). We aimed to understand whether histones obtained by non-invasive techniques such as buccal swabbing or minimally invasive peripheral blood collection can accurately consolidate the objective and subjective benefits of exercise on human health. We employed mass spectrometry to quantify methylation and acetylation patterns of histone H3 and H4 in buccal cells and peripheral blood mononuclear cells based on a previously established workflow, a method that gives an overview of a global PTM mosaic over a targeted approach (25). This yielded a set of epigenetic markers that accurately indicates whether a sample was collected before or after the sport therapy. We observed a predominant reduction in methylation of histone H3 and H4 and an increase of H3.1 K27ac. This goes in hand with published knowledge that such modifications support transcription activation and protein production (63–66). An increase in H3.1 K27ac, a prominent marker of enhancer activity, displayed a predominantly positive correlation with increased expression of plasma proteins while a decrease in H3 K56me1 negatively correlated with the same proteins (23). McGee et al. reported a global increase in H3 K36ac and no change in H3 K9/14ac, the latter associated with transcription initiation, followed by a single round of exercise on the skeletal muscle (24, 67). This concurs with our data; however, by using mass spectrometry, we can distinguish that the overall increase of H3 K36ac is driven by H3.3 and not H3.1 K36ac, as well as the general increase of H3 K9ac and decrease of H3 K14ac could lead to no obvious change using antibody-based approaches due to limits of selectivity.

Human plasma proteome analysis offers a unique dynamic range, efficiently captured by mass spectrometry, that reflects subtle, but systemic changes in the organism. Plasma proteins are key players in immune and inflammatory response, hormonal regulation, and transport of lipids, drugs, and minerals (59). However, functional buffering of plasma proteins that ensures elementary robustness for homeostasis might shadow subtle long-term effects experienced during lifestyle or therapeutic interventions. Therefore, we established a set of plasma markers whose levels accurately separate participants by their therapy status (before or after the therapy). We then aimed to find common biological information from these underlying proteomic changes upon therapy with epigenetic reprogramming. We obtained 6 PTMs from PBMCs and 2 from buccal cells with a strong correlation to 27 proteomic markers, which we annotated with their GO biological process to visualize a functional PTM—protein network. H3.1K27ac, H3.1K36me1, H3.1K36me3, H3.3K27me2, H3K14ac, and H3K79me2 PBMC markers predominantly displayed correlation with response of the immune system (innate immunity, inflammatory response, defense response to bacteria), cell division, and organization (cell migration, proliferation, apoptosis, cell shape, cell adhesion) as well as potentially pathways directly connected to physical activity (cardiac muscle contraction, bone remodeling, ATP-dependent activity). H3.1K36ac and H3K56me1 markers obtained in the buccal cells converge into strikingly similar patterns of biological processes, i.e., immune response, inflammatory response, cell shape, and chromatin organization. This is likely constrained by functional information obtained from plasma proteome, but it also indicates that both sample types can be used as a source of epigenetic information, confirmed by the high preservation of inter-participant distances across omic types. This suggests that despite our limited sample size, the biological information content outperforms noise—noise in the sense of daily or even hourly fluctuations of omic profiles or technical perturbation of the signal measurements. This could be mitigated by the fact that therapy outcomes are measured on the same individuals and more generally by the constraint of the therapy attendance toward an overall improvement.

Sport therapy benefits vary within the cohort, apart from the tendency to implement healthier nutritional and exercise habits, participants exhibited physical (agility gain, back pain degree) and molecular (distance toward healthy markers) adaptations. We are aware that the moderate sample size is a limitation of the study (also due to the high dropout rate potentially due to the SARS-CoV-2 pandemic), which we have addressed by using narrow inclusion criteria (25–35 age group, men). Nevertheless, it restricts the portability of our findings to the general population (e.g., older individuals, women, non-European ancestry).

Therapy effects on health awareness indicators are disparate across biological samples. This might result from the hierarchical nature of the molecular data and their functional relationship (68) suggested by the reconstructed functional network. Many intermediate phenotypes lie in the path between diet and/or exercise intervention and health outcomes (69, 70). To better understand the causal chain, a deeper characterization of the hierarchical effect of the biomarkers is needed in the context of back pain. However, this would require overcoming methodological difficulties of such so-called mediation analysis in establishing causality, since lifestyle components are convoluted and (un)measured confounding can occur (e.g., smoking status, BMI, age, education, and alcohol intake), an issue is previously discussed (71). To bypass these challenges, we took advantage that each participant is his own control and built statistical checkpoints (intersection of classic and machine learning procedures, unsupervised clustering, data integration) to obtain reliable lists of top markers. Because therapy response is a complex trait, we additionally quantified the therapy effect on health awareness indicators, by incorporating the cumulative effect of each molecular contributor. We aim to tackle these obstacles in the future, in line with a more personalized monitoring using longitudinal profiling to obtain a more robust set of epigenetic markers validated in an independent cohort.

## 5 Conclusion

This experimental cohort study provides initial evidence that (1) composite epigenetic biomarkers obtained from minimally invasive, readily available, and sample sources (buccal swabs) can accurately reflect a person's therapy status—before and after standardized sport therapy, despite complex history and lifestyle influences. (2) Back pain and agility improvement matches a reduction in H3 and H4 methylation on several markers, independently of the sample type (buccal cells and PBMCs), and an increase in H3.1 K27ac, patterns that correlate with increased immune response in plasma. (3) Integrating (objective) molecular changes and (subjective) self-rated evaluation of therapy, we found agreement between epigenetic profiles and lifestyle categories, highlighting the potential of the epigenetic markers to illustrate participants' therapeutic journey. More efforts are needed to complement the epigenetic landscape of back pain and validate their robustness and potential to predict such health phenotypes.

## Data Availability

The mass spectrometry proteomics data have been deposited to the ProteomeXchange Consortium via the PRIDE [72] partner repository with the dataset identifier PXD050113. Intermediate results files (processed questionnaire; participants classes; agility measurements; plasma protein abundances; buccal cell and PBMC PTMs abundances and relative abundances; test outcomes) are available in the https://doi.org/10.5281/zenodo.10685544 Zenodo repository.
