# Supplementary material for: Back pain exercise therapy remodels human epigenetic profiles in buccal and human peripheral blood mononuclear cells: an exploratory study in young male participants
Source: Front Sports Act Living. 2024 Oct 16;6:1393067. doi: 10.3389/fspor.2024.1393067 (PMC11521823; doi:10.3389/fspor.2024.1393067)
Supplement: Supplementary file 1 [file Table1.docx]

Supplementary Material 1

**Backpain exercise therapy remodels human histone epigenetic profiles in buccal and human peripheral blood mononuclear cells**

**Claire Burny^†^, Mia Potočnjak^†^, Annika Hestermann, Sophie Gartemann, Michael Hollmann, Frank Schifferdecker-Hoch, Nina Markanovic, Simone Di Sanzo, Michael Günsel, Victor Solis-Mezzarino^†^, Moritz Voelker-Albert^†^***

† Equal contribution and first authorship

† Equal contribution and last authorship

*** Correspondence:** Moritz Voelker-Albert: moritz@moleqlar.de

**Supplementary Table 1.** List of inclusion and exclusion criteria followed for therapy recruitment.

| **Inclusion criteria** | |
| --- | --- |
| Age | 25-35 years old |
| Sex | Men |
| Physical condition | No professional sport |
| **Exclusion criteria**  Relative contraindication means that the treatment could be acceptable if the benefits outweigh the risks, while absolute contraindication describes condition that could cause a life-threatening result if the participant undergoes the treatment. | |
| Spinal disorders | Relative contraindications: herniated disc with root irritation symptoms; Operated disc hernia up to 3 months postoperatively. |
|  | Absolute contraindications: herniated disc with acute radicular symptoms/root compression symptoms; Decompensated spondylolisthesis (Meyerding grade 3 or 4); Thoracolumbar scoliosis (Cobb angle >40°). |
| Bone and joint diseases | Relative contraindications: osteoporosis (without fracture) |
|  | Absolute contraindications: Osteoporosis with manifestation (with fractures); Inflammatory systemic diseases (rheumatism, Bechterew's disease) at acute stage; Fresh fractures (4 months) |
| Internal diseases | Relative contraindications: Diabetes mellitus (requiring insulin); coronary heart disease with reduced exercise capacity; Relative heart failure |
|  | Absolute contraindications: Severe vascular diseases (e.g., aortic aneurysm, pulmonary embolism, phlebothrombosis, Arterial occlusive disease); Severe heart and circulatory diseases (e.g., unstable congenital heart defects and heart failure, cardiomyopathy). |
| Other diseases | Relative contraindications: Current tumor disease; Mental illness |
|  | Absolute contraindications: All acute diseases requiring surgery; Abdominal or gynecological surgery within the last 3 months (or as determined by the surgeon); incisional and abdominal wall hernias; Neurological diseases with progressive symptoms; retinal detachment and glaucoma. |

**Supplementary Table 2.** Description of the group of questions characterizing the Individual background.

| **Questions** | **Variable type [range] or (re-encoded values)** | **Imputed value** | **Binning** | **Is measured BT^(a)^ and AT^(a)^? If so, report the test outcome.** |
| --- | --- | --- | --- | --- |
| Body Mass Index (from weight/size^2^, weight in kg and size in m) | quantitative | average weight between BT and AT | normal ]18.5, 24.9]  overweight ]24.9, 29.9]  obese >29.9 | paired t-test from unbinned values: t(11)=0.99; p=0.34 |
| smoking | binary ^(b)^ | - ^(c)^ | - | 1 smoker only, n.c.^(d)^ |
| age | bounded quantitative [25, 35] | age at blood test | - | - |
| COVID infection ^(e)^ | binary | no | - | - |

1. BT means before therapy, and AT means after therapy
2. From now on, binary variables are encoded as follows: 0 (no) and 1 (yes).
3. The “-“ symbol indicates either no imputation, no binning, or no test performed as not pertinent.
4. n.c.: no change and no test has been performed, e.g. in the case of the same answers before and after therapy.
5. No information as regards the time of infection has been collected.

**Supplementary Table 3.** Description of the group of questions characterizing the Socio-demographic environment.

| **Questions** | **Variable type [range] or (re-encoded values)** | **Imputed value** | **Binning** | **Is measured BT and AT? If so, report the test outcome.** |
| --- | --- | --- | --- | --- |
| Relationship | categorical  single (0)  in couple (1)  married (1) | - | single or in couple | McNemar test: t(1)=0; p=1 |
| Having children | binary | - | - | 1 participant only, n.c. |
| Educational attainment | categorical  completed education (0)  high school (1)  college degree (2)  university (3) | - | - | n.c. |
| Work status | categorical  employee (0)  manager (1)  leader (1) | - | employee (0) or leadership position (1) | McNemar test: t(1)=0; p=1 |
| Life event impact ^(a)^ | Likert-like score [-1, 0, 1, 2] | 0 | - | Friedman test:  χ^2^(1)=1.8, p=0.18 |
| Social integration | binary | - | - | McNemar test: t(1)=0; p=1 |

1. This variable is accompanied by textual information describing the events/changes in life but since different persons can be impacted differently by similar events, we only retained the self-grading information.

**Supplementary Table 4.** Description of the group of questions characterizing the Dietary habits class.

| **Questions** | **Variable type [range] or (re-encoded values)** | **Imputed value** | **Binning** | **Is measured BT and AT? If so, report the test outcome.** | **Included in PAM ^(a)^**  **y/n ^(b)^** |
| --- | --- | --- | --- | --- | --- |
| Weekly fresh cooking days | integer [0, 7] | - | - | Exact Wilcoxon signed rank test: V=15.5; p=0.80 | y |
| Weekly fruits vegetables days | integer [0, 7] | - | - | V=32; p=0.31 | y |
| Weekly meat days | integer [0, 7] | - | - | V=10.5; p=0.67 | y |
| Weekly sweets/snacks days | integer [0, 7] | - | - | **V=42; p=0.02** | y |
| Weekly intake 2L fluids days | integer [0, 7] | - | - | V=14.5; p=1 | n |
| Weekly sugary drinks days | integer [0, 7] | - | - | V=6; p=0.09 | n |
| Weekly alcohol days | integer [0, 7] | - | - | V=3; p=0.38 | n |
| Vegetarian/vegan | binary | - | - | McNemar test: t(1)=0; p=1 | n (nested within “Weekly meat days”) |
| Nutritional score | score ^(c)^ | - | - | Exact Wilcoxon signed rank test: V=13; p=0.57 | n (considers additional information, drink intakes) |

1. PAM refers to the Partitioning Around Medoid algorithm (Kaufman et al., 1987)
2. “y” for yes and “n” for no
3. See Table S5

**Supplementary Table 5.** Weighting scheme of our in-house nutritional score.

| **Questions** | **Days-per-week bin (grade) ^(a)^** | | |
| --- | --- | --- | --- |
| Weekly fresh cooking days | [0, 2] (0) | [3, 4] (1) | [5, 7] (2) |
| Weekly fruits vegetables days | [0, 2] (0) | [3, 4] (1) | [5, 7] (2) |
| Weekly meat days | [0, 2] (1) | [3, 4] (2) | [5, 7] (0) |
| Weekly sweets/snacks days | [0, 2] (2) | [3, 4] (1) | [5, 7] (0) |
| Weekly intake 2L fluids days | [0, 6] (0) | [7] (1) |  |
| Weekly sugary drinks days | [0, 2] (2) | [3, 4] (1) | [5, 7] (0) |
| Weekly alcohol days | [0, 4] (1) | [5, 7] (0) |  |

1. The nutritional scores both before and after therapy for a given individual were then obtained as the sum of the graded answers detailed in the table above. Thresholds have been used from (Commission, 2022) and (Odegaard et al., 2010).

**Supplementary Table 6.** Description of the group of questions characterizing the Exercise class.

| **Questions** | **Variable type [range] or (re-encoded values)** | **Imputed value** | **Binning** | **Is measured BT and AT? If so, report the test outcome.** | **Included in PAM y/n** |
| --- | --- | --- | --- | --- | --- |
| Doing regular sport | binary | - | - | McNemar test: t(1)=0.5; p=0.48 | n (nested within the next question) |
| Number of sports | integer [0, 3] | - | Sum of practiced sports from the following list: endurance sport, weight training, team sport | Exact Wilcoxon signed rank test: V=2.5; p=0.63 | y |
| Increasing activity in daily life | binary | - | - | McNemar test: t(1)=0.5; p=0.48 | y |
| Doing WHO-recommended activity (weekly moderate exercise >150mn or weekly intense exercise >75mn, (Organization & others, 2020)) | binary | - | - | McNemar test: t(1)=0; p=1 | y |
| Daily hours sitting | bounded discrete quantitative [0, 24] | - | - | Exact Wilcoxon signed rank test: V=39; p=0.31 | n (biserial correlation of -0.98 with Sedentary behavior) |
| Sedentary behavior | ordinal  predominantly sedentary (0)  occasional (1)  constantly moving (2) | - | - | Friedman test:  χ^2^(1)=1, p=0.32 | y |

**Supplementary Table 7.** Description of the group of questions characterizing the Backpain status.

| **Questions** | **Variable type [range] or (re-encoded values)** | **Imputed value** | **Binning** | **Is measured BT and AT? If so, report the test outcome.** | **Included in PAM y/n** |
| --- | --- | --- | --- | --- | --- |
| Having current backpain | binary | Shall be yes if the next three answers differ from 0 | - | McNemar test: t(1)=0.25; p=0.62 | n (nested within backpain frequency and intensity) |
| Number of  weeks with  backpain | integer | 0 | - | Exact Wilcoxon signed rank test: V=10; p=0.57 | y |
| Backpain frequency | ordinal  symptom-free (0)  unregularly (1)  regularly (2)  constant (3) | 0 |  | Friedman test:  χ^2^(1)=1.29, p=0.26 | y |
| Backpain intensity | ordinal [0, 10] from less to more intense | 0 |  | **Friedman test:**  χ^2^**(1)=2, p=0.16** | y |
| Taking medication | binary | - |  | n.c. | n |
| Backpain associated with work | binary | no |  | - | n |

**Supplementary Table 8.** Description of the group of questions characterizing the Backpain history classes.

| **Questions** | **Variable type [range] or (re-encoded values)** | **Imputed value** | **Binning** | **Comments** | **Included in PAM y/n** |
| --- | --- | --- | --- | --- | --- |
| **Overall history** | | | | | |
| Amount of past backpain years | ordinal  0 year (0)  ]0, 1] year (1)  ]1, 5] years (2)  >5 years (3) | 0 | Binned by years range | - | y |
| **Last 3 months** | | | | | |
| Number of backpain days | ordinal  0 day (0)  ]0, 7] days (1)  ]7, 31] days (2)  >31 days (3) | 0 | Binned monthly | - | y |
| Percentage of backpain days with restricted activity | ordinal  0% (0)  ]0, 50[% (1)  >= 50% (2) | 0 | Obtained from the % of reported days with restricted activity, then binned | - | y (polychoric correlation up to 0.98 in absolute value, but included at 12 months) |
| Number of medical visits | integer | 0 | - | Only one person had one visit | n (not enough variability) |
| Number of sickleave days | binary  0 days (0)  >0 day(s) (1) | 0 | Binned | n.c. none | n |
| Number of hospital cure days | integer | 0 | - | n.c. none | n |
| Number of stay-in-bed days (due to backpain) | binary  0 days (0)  >0 day(s) (1) | 0 | Binned | n.c. none | n |
| Backpain medication frequency | ordinal  None (0)  1/2x yearly (1)  1/2x monthly (2)  1/2x weekly (3)  Daily (4) | 0 | - | - | y |
| Number of therapy sessions | ordinal  0 session (0)  ]0, 10] sessions (1)  >10 sessions (2) | 0 | Sum of sessions among physiotherapy, massage, pack, and electrodes sessions, then binned | - | y |
| **Last 12 months** | | | | | |
| Number of backpain days | ordinal  0 day (0)  ]0, 31] days (1)  ]31, 91] days (2)  >91 days (3) | 0 | Binned monthly | - | y |
| Percentage of backpain days with restricted activity | ordinal  0% (0)  ]0, 50[% (1)  >= 50% (2) | 0 | Obtained from the % of reported days with restricted activity, then binned | - | y |
| Number of medical visits | integer | 0 | - | - | n (polychoric correlation up to .83) |
| Number of sickleave days | Binary  0 days (0)  >0 day(s) (1) | 0 | Binned | Only one person had 3 days sick leave | n (not enough variability) |
| Number of hospital cure days | integer | 0 | - | n.c. none | n |
| Number of stay-in-bed days (due to backpain) | binary  0 days (0)  >0 day(s) (1) | 0 | Binned | - | n (not included over last 3 months) |
| Backpain medication frequency | ordinal  None (0)  1/2x yearly (1)  1/2x monthly (2)  1/2x weekly (3)  Daily (4) | 0 | - | - | y |
| Number of therapy sessions | ordinal  0 session (0)  ]0, 10] sessions (1)  >10 sessions (2) | 0 | Sum of sessions among physiotherapy, massage, pack, and electrodes sessions, then binned | - | y |

**Supplementary Table 9.** Summary table of backpain and lifestyle classes, as well as lifestyle parameters.

| **Class** | **Cluster (n=number of participants at BT)** | | | | | | |
| --- | --- | --- | --- | --- | --- | --- | --- |
| **Dietary habits ^(b)^** | **Towards poorly diversified (lack of antioxidants, vitamins, minerals) (n=4)** | | | **Towards Western-like (meat- & snacks-rich) (n=3)** | | **Towards balanced/flexitarian (n=5)** | |
|  | **mean [+/- sd] ^(a)^ (medoid)** | | | **mean [+/- sd] ^(a)^ (medoid)** | | **mean [+/- sd] ^(a)^ (medoid)** | |
| **Weekly fresh cooking days ^(c)^**  **Weekly fruits vegetables days**  **Weekly meat days**  **Weekly sweets/snacks days**  Weekly intake 2L fluids days  Weekly sugary drink days  Weekly alcohol days  Vegetarian or vegan (no/yes)  Nutritional score | **2.2 [+/- 2.1] (2)**  **2.5 [+/- 1.0] (3)**  **1.8 [+/- 1.0] (1)**  **3.0 [+/- 1.4] (2)**  5.8 [+/- 1.5] (4)  0.8 [+/- 1.0] (2)  0.5 [+/- 1.6] (1)  4/0 (0)  7.2 [+/- 1.3] (7) | | | **4.3 [+/- 0.6] (4)**  **4.3 [+/- 0.6] (4)**  **5.0 [+/- 0.0] (5)**  **5.3 [+/- 2.1] (6)**  6.3 [+/- 1.2] (7)  2.0 [+/- 1.0] (3)  1.7 [+/- 1.2] (3)  3/0 (0)  6.3 [+/- 1.2] (5) | | **5.4 [+/- 1.3] (4)**  **6.2 [+/- 0.8] (6)**  **2.2 [+/- 1.8] (2)**  **2.6 [+/- 0.9] (2)**  5.2 [+/- 1.1] (5)  1.0 [+/- 1.0] (2)  1.2 [+/- 1.3] (0)  4/1 (0)  9.2 [+/- 0.4] (9) | |
| **Exercise ^(b)^** | **Towards sedentary (n=3)** | | | **Towards active (n=2)** | | **Towards sportive (n=7)** | |
| **Number of sports**  **Increasing daily activity (no/yes)**  **Doing WHO-recommended activity (no/yes)**  Daily hours sitting  **Sedentary behavior**  mostly sedentary (0), occasional (1), constantly moving (2) | **0.0 [+/- 0.0] (0)**  **0/3 (1)**  **3/0 (0) iiiiiiiiiiiiiiiiiiiiiiiiiiiiiiiiiiiiiiiiiii**  8.5 [+/- 5.4] (14)  **0.3 [+/- 0.6] (0)** | | | **0.5 [+/- 0.7] (1)**  **0/2 (1)**  **0/2 (1) iiiiiiiiiiiiiiiiiiiiiiiiiiiiiiiiiiiiiiiiiiiiiiiiiiiiii**  5.0 [+/- 1.4] (3)  **2.0 [+/- 0.0] (2)** | | **1.1 [+/- 0.7] (1)**  **2/5 (1)**  **0/7 (1) iiiiiiiiiiiiiiiiiiiiiiiiiiiiiiiiiii**  11.9 [+/- 2.1] (11)  **0.1 [+/- 0.4] (0)** | |
| **Backpain history (1 year) ^(b)^** | **strong**  **>5 years**  **(n=2)** | | **intermediate**  **>1-5 year(s)**  **(n=3)** | | **episodic**  **no yearlong**  **(n=4)** | | **no/minor**  **<6 months**  **(n=3)** |
| **Amount of past backpain years**  **Number of backpain days**  **Percentage of backpain days with restricted activity**  Number of medical visits  Number of sickleave days  Number of stay-in-bed days (due to backpain)  **Backpain medication frequency**  None (0), 1/2x yearly (1), 1/2x monthly (2), 1/2x weekly (3), daily (4)  **Number of therapy sessions** | **10.5 [+/- 4.9] (7)**  **225.0 [+/- 63.6] (180)**  **0.1 [+/- 0.1] (0.17) iiiiiiiiiiiiiiiiiiiiiiiiiiiiiiiiiiiiiiiiiii**  2.5 [+/- 2.1] (1)  1.5 [+/- 2.1] (0)  2.0 [+/- 2.8] (4) **iiiiiiiiiiiiiiiiiiiiiiiiiiiiiiii**  **1.5 [+/- 0.7] (2)**  **13.5 [+/- 17.7] (1)** | | **4.3 [+/- 0.6] (4)**  **50.0 [+/- 17.3] (30)**  **0.1 [+/- 0.0] (0.17) iiiiiiiiiiiiiiiiiiiiiiiiiiiiiiii**  0.7 [+/- 0.6] (0)  0 (0)  0.7 [+/- 1.2] (2) **iiiiiiiiiiiiiiiiiiiiiiiiiiiiiiii**  **0.3 [+/- 0.6] (0)**  **1.0 [+/- 1.7] (3)** | | **0 (0)**  **18.2 [+/- 13.9] (3)**  **0.3 [+/- 0.2] (0.5) iiiiiiiiiiiiiiiiiiiiiiiiii**  0.5 [+/- 0.6] (0)  0 (0)  0.2 [+/- 0.5] (0) **iiiiiiiiiiiiiiiiiiiiiiiiii**  **0 (0)**  **9.0 [+/- 14.2] (1)** | | **0.2 [+/- 0.3] (0)**  **26.7 [+/- 46.2] (0)**  **0 (0) iiiiiiiiiiiiiiiiiiiiiiiiiiiiiiii**  0 (0)  0 (0)  0 (0) **iiiiiiiiiiiiiiiiiiiiiiiiii**  **0 (0)**  **0 (0)** |
| **Backpain history (3 months) ^(b)^** | **strong**  **(n=1)** | **intermediate**  **(n=1)** | | **episodic**  **(n=3)** | | **no/minor**  **(n=7)** | |
| **Number of backpain days**  **Percentage of backpain days with restricted activity**  Number of medical visits  **Backpain medication frequency**  **Number of therapy sessions** | **60**  **0.5 iiiiiiiiiiiiiiiiiiii**  0  **2**  **0** | **20**  **0.25 iiiiiiiiiiiiiiiiiiii**  0  **0**  **0** | | **14.0 [+/- 18.2] (5)**  **0 (0) iiiiiiiiiiiiiiiiiiiiiiiiiiiiiiiiiiiiiiiiiiiiiiiiiii**  0 (0)  **0 (0)**  **3.3 [+/- 1.5] (3)** | | **1.3 [+/- 2.4] (0)**  **0 (0) iiiiiiiiiiiiiiiiiiiiiiiiiiiiiiiii**  0.1 [+/- 0.4] (0)  **0.1 [+/- 0.4] (0)**  **0 (0)** | |
| **Current backpain ^(b)^** | **chronic**  **(n=2)** | | **acute/frequent**  **(n=3)** | | **acute/infrequent**  **(n=2)** | | **no/minor**  **(n=5)** |
| Reported backpain (no/yes)  **Weeks with backpain**  **Frequency**  no symptom (0), unregular (1), regular (2), constant (3)  **Intensity**  ordinal [0, 10] from less to more intense  **Taking medication for backpain (no/yes)**  Is backpain associated with work? (no/yes) | 0/2 (1)  **5.5 [+/-0.7] (6)**  **2.0 [+/- 0.0] (2)**  **5.0 [+/- 0.0] (5)**  **1/1 (0)**  1/1 (no) | | 0/3 (1)  **0.7 [+/- 0.6] (1)**  **2.3 [+/- 0.6] (2)**  **4.3 [+/- 1.5] (4)**  **2/1 (1)**  0/3 (no) | | 0/2 (1)  **1 [+/- 0.0] (1)**  **1.0 [+/- 0.0] (1)**  **4.5 [+/- 2.1] (4)**  **1/1 (0)**  1/1 (no) | | 3/2 (0)  **0 (0)**  **0.2 [+/- 0.4] (0)**  **0.2 [+/- 0.4] (0)**  **4/1 (0)**  3/2 (no) |
| **Punctual event & socio-demographic environment** | 6/6  -1 (1), 0 (7), 1 (2), 2 (2)  completed education (2), high school (1), college degree (2), university (7)  9/3  1/11  8/4  11/1  5/7 **iiiiiiiiiiiiiiiiiiiiiiiiiiiiiiiiiiiiiiiiiiiiiiiiiiiiiiiiiiiiiiiiiiiiiiiiiiiiiiiiiiiiiiiiiiiiiiiiiiiiiiiiiiiiiiiiiiiiiiiiiiiiiiiiiiiiiiiiiiiiiiiiiiiiiiiiiiiiiii**  1/11 | | | | | | |
| Covid (no/yes)  Life impact  Educational attainement  Leadership position (no/yes)  Social integration (no/yes)  In couple (no/yes)  Has children (no/yes)  Is backpain associated with work? (no/yes)  Smoking (no/yes) |  |  |  |  |  |  |  |

1. All numbers shown are either mean values with standard deviations in brackets and medoids in parentheses or represent the number of answers per category.
2. Classification was obtained from the participants´ answers to questions in bold (see Supplementary Material 1, Methods). Nested variables or variables with the same answers for all participants are not reported.
3. Questions in bold are the ones that contribute to the clustering.

**Supplementary Table 10.** Gaussian Mixture Model evaluation of the Performance class.

| **G (number of components)** | **Covariance structure (from best-BIC)** | **Bayesian Information Criterion (BIC)** | **Integrated Completed Likelihood** (Biernacki et al., 2000) |
| --- | --- | --- | --- |
| 1 | EEE  (ellipsoidal, equal volume, shape and orientation) | -497.63 | -497.63 |
| 2 | EVI  (diagonal, equal volume, varying shape) | -480.19 | -480.19 |
| 3 | EII  (spherical, equal volume) | -486.82 | -487.71 |
| 4 | EEI  (diagonal, equal volume and shape) | -472.34 | -472.34 |

**Supplementary Table 11.** Estimation of Gaussian Mixture Model parameters and their 95% confidence interval.

| **Cluster (n=number of participants)** | **Mixing proportions** | **Flexion (% of change)** | **Extension (% of change)** | **Rotation right (% of change)** | **Rotation left (% of change)** |
| --- | --- | --- | --- | --- | --- |
| Moderate (n=7) | 0.58 [0.33, 0.82] | 10.87 [3.45, 18.28] | 43.11  [18.87, 67.36] | 32.47  [24.44, 40.51] | 26.93  [13.59, 40.28] |
| Intermediate (n=3) | 0.25 [0.00, 0.49] | 45.35 [23.28, 67.42] | 56.66 [21.02, 92.30] | 78.34 [50.18, 106.49] | 63.42 [46.13, 80.71] |
| Max (n=1) | 1/12 | 33.87 | 27.04 | 116.04 | 268.88 |
| Max (n=1) | 1/12 | 131.16 | 39.93 | 103.82 | 121.94 |

**Supplementary Methods**

Data processing and analyses have been performed with the R software (versions 4.22.32 and 4.3.2; R Core Team, 2022). Some plots have been generated with the ggplot2 R package (version 3.4.41; Wickham, 2016) - helper functions being indicated in the sub-sections.

**Questionnaire data management**

We retained the data of 12 out of 19 participants that followed the therapy entirely, *i.e.,* whose agility metrics have been measured before and after the therapy (encoded as BT and AT respectively) and with at least two sets of usable paired (for BT and AT) datasets among the buccal cells, peripheral blood mononuclear cells (PBMC), and plasma samples (which processing is described in the “**Proteomics data processing”** section).

Over all questions, different types of variables were collected: i) “directly targeted” by the therapy or response variables (agility metrics and quality of life scores), ii) lifestyle indicators monitored during the study which may or may not change upon therapy, and iii) past/history indicators that do not depend on the therapy (e.g., backpain history, detailed below).

All participants followed at least 20 out of 24 sessions (7 completed the full number of sessions) from over 16 to 25.6 weeks.

We grouped questions relative to the **individual background** (Table S2) and the **social environment** (Table S3), and then built lifestyle profiling.

**Lifestyle indicators**

Classes of variables were defined to identify homogeneous clusters of participants within the cohort with shared/similar patterns of answers. The subsequent categories reflect latent - in sense not directly observable - profiles of participants´ backpain history, and lifestyle, which might display (in)direct associations with the response to therapy.

For each class of variables, we first evaluated which questions contribute to the clustering in order i) to obtain categories that make sense, ii) to avoid multi-collinearity, iii) to remove as much as possible composite, and nested questions (which filtering is described in the supplementary tables). Second, we opted for cluster analysis instead of a probabilistic approach such as latent class modeling due to our moderate sample size; therefore, we do not have any probability that a given participant belongs to each cluster. We computed pairwise dissimilarity using the Gower distance (Gower, 1971) adapted for data of mixed types, using the cluster::*daisy* R function (version 2.1.4; Maechler et al., 2022). We then applied the Partitioning Around Medoids algorithm (Kaufman et al., 1987) implemented in the cluster::*pam* (*nstart=100*) R function. The number of clusters - evaluated from 2 to 4, considering the cohort size and the objective of obtaining clusters that are interpretable - was supported by the silhouette-width coefficient (Rousseeuw, 1987). Third, after obtaining the clusters, we reported the medoids as clusters´ representatives, and summary statistics of answers´ distribution per cluster to eventually interpret and to label the clusters with a health gradient, from less to more beneficial for the health.

To assess the dynamics of the classes, *i.e.,* if a given participant changes its category upon therapy, we used the ggalluvial::*geom_flow* R function (version 0.12.5; Brunson & Read, 2023) to represent each participant´s trajectory.

- The **Dietary habits** class (Tables S4, S5). Four variables describing Dietary habits have been included. Pairwise correlations - computed with DescTools::*KendallTauB* R function (version 0.99.547; Andri et mult. al., 2022) – reached a maximum (in absolute value) of 0.58. Three clusters – with a silhouette -width value of 0.24, 0.39 and 0.28 for 2, 3 and 4 clusters - have been retained with the following labeling: “Towards balanced/flexitarian”, “Towards Western-like (meat- & snacks-rich diet)”, and “Towards undiversified (potential lack of antioxidants, vitamins, and minerals)”, from more to less healthy respectively (Supplementary Material 2: Fig. S1).
- The **Exercise** class (Table S6). Note that we did not analyze the outcome of the question regarding the practice of sports during the pandemic. Four variables describing Exercise have been included. Pairwise correlation - computed with psych::*mixedCor* R function (version 2.42.19; Revelle, 2022) - reached a maximum (in absolute value) of 0.59. We got one correlation of 1 between WHO and regular sport answers, although decided to retain both answers since 2 and 1 person(s) in BT and AT respectively reported following WHO recommendation without practicing regular sports. Three clusters – with a silhouette -width value of 0.59, 0.59 and 0.58 for 2, 3 and 4 clusters - have been retained with the following labeling: “Towards sportive”, “Towards active”, and “Towards sedentary”, from more to less healthy respectively (Supplementary Material 2: Fig. S2).

**Backpain characterization**

We created classes of variables as follows:

- The **Backpain status** class (Table S7). Three variables describing the current Backpain status have been included. Pairwise correlation - computed with psych::*mixedCor* R function - reached a maximum (in absolute value) of 0.82. Four clusters – with a silhouette -width value of 0.60, 0.67 and 0.64 for 2, 3 and 4 clusters - have been retained with the following labeling: “no/minor”, “acute/less frequent”, “acute/more frequent”, “chronic”, ranked by backpain severity (Supplementary Material 2: Fig. S3A). Because this class was defined from self-rated scores – no therapist-biased -, we used it to evaluate the outcome of therapy under an additional “Self-assessment” class whose categories were labeled as follows: “Unimpaired” (for participants who remained in the “no/minor” category), “Improving”, “Stagnating”, and “Worsening” scenarios (Fig. 3C-E).
- The **Backpain history** class (Table S8). Variables describing Backpain history over the last 12 and 3 months have been included. Pairwise correlation - computed with psych::*mixedCor* R function – reached a maximum (in absolute value) of 0.77. Four clusters – with a silhouette -width value of 0.49 (0.62), 0.36 (0.59) and 0.46 (0.59) for 2, 3 and 4 clusters over the last year (3 months) - have been retained with the following labeling: “no/minor & <6 months”, “episodic & <1 year", "intermediate & >1 year", and "strong & >5 years", for the last 12 months and “no/minor”, “episodic", "intermediate", and "strong", for the last 3 months (Supplementary Material 2: Fig. S3 B and C). Note that although the silhouette coefficient suggested the usage of 2 clusters, we retained the second-best silhouette value to better characterize backpain history.

**Background similarity between participants**

Each participant was classified into categories that describe his nearest typical Dietary, Exercise, and Sedentary habits, as well as Backpain history and current backpain status (summary Table S9). We used the TraMineR::*seqdf* R function (version 2.2.9-6; Gabadinho et al., 2011) followed by ggseqplot::*ggseqiplot* R function (version 0.8.31; Raab, 2022) to get an overview of lifestyle heterogeneity/similarity within the cohort, by representing one participant as a stacked sequence of lifestyle components (Fig. 2A). Each participant was ranked from the distance to the healthier participant defined as the one belonging to the highest number of green/healthy categories, using increased pairwise Gower distance.

Because lifestyle components can be dependent, we computed the polychoric correlation matrix of inter-cluster membership using the psych::*polychoric* R function represented with the corrplot::*corrplot* R function (version 0.92; Wei & Simko, 2021) to measure to what extent lifestyle and backpain components tend to overlap (Fig. 2C).

**Agility metrics**

We collected individual measurements of isometric maximum strength (Nm) in trunk flexion/extension, lateral flexion, and axial rotation before and after the therapy. The therapy effectiveness was measured as the percentage of change of each agility metric – positive in case of improvement upon therapy. Because participants have different physical characteristics (e.g. size), the percentages of change were measured from baseline-adjusted values, computed from the uncorrected values minus the individual average over BT and AT plus the overall mean per agility metric. To reveal the therapy responses´ heterogeneity within the cohort, we used the Gaussian mixture modeling (GMM) framework as a data-driven partitioning of therapy responders/performers. As the input of the mclust::*Mclust* R function (version 6.0.10; Scrucca et al., 2016), we used only documented enough quantitative metrics (*i.e.*, without missing values; flexion, extension, rotation) and retained both rotation left and right percentages of change - though correlated it does not necessarily mean rotation shall be symmetric (Spearman’s rank correlation; r(36)=0.87; p=3.09×10^-4^). We eventually fitted GMMs with up to G=4 components, which parameters were estimated by maximization of the likelihood with a modified Expectation–Maximization algorithm (Dempster et al., 1977). The mixing proportions p_g_ can be viewed as a prior probability that any individual belongs to the *g*^th^ component. To retain the best model, we used likelihood-based criteria, the Bayesian information criterion (BIC), jointly with the integrated completed likelihood criterion or ICL (Biernacki et al., 2000). Each individual was assigned to the component corresponding to the highest component membership *a posteriori* probability (so-called MAP rule). We retained 4 clusters, confirmed by sequential Likelihood Ratio Tests (LRT), using the mclust::*MclustBootstrapLRT* R function (modelName=“EII“, type=“jk“; maxG=3; 1 vs 2: LRT stat=20.85, p=0.014; and 2 vs 3: LRT stat =20.21, p=0.012; 3 vs 4: LRT stat=29.15, p=0.005), that led to three **Performance** categories (Table S10, Fig. 3A).

The 95% confidence interval (CI) were reported for estimated parameters of the GMM by Jackknife using the mclust::*MclustBootstrap* R function (Table S11).

Both wellbeing and physical capacity Likert-like cores, bounded from 0 to 5, were self-rated before and after the therapy, and differences in the scoring were assessed with a Friedman test. The distribution of both scores over self-assessment categories was represented as a sunburst plot (Fig. 3F).

**LC-MS/MS data quantification and processing**

**Histone modifications.** Raw files were analyzed using the Skyline software (MacLean et al., 2010), as previously described in (Van den Ackerveken et al., 2021). The log2-transformed normalized intensities are used for subsequent processing. First, we applied quantile normalization using the preprocessCore::*normalize.quantiles* R function (version 1.6058.0; Bolstad, 2022). Second, we imputed missing values depending on not missing at random (NMAR) or Missing At Random (MAR) values (see main Methods). Third, we used an empirical Bayesian framework to adjust intensities for batch effect using the sva::*ComBat* R function (version 3.46.0; (Leek et al., 2022); par.prior = F, prior.plots = F, mean.only = F, model=model.matrix(~therapy status, data = design)). Quality control principal component analysis (PCA) plots after each processing step are represented in **Supplementary Material** 2 (Fig. S6). Eventually, we computed the relative abundance (percentage) of each modified peptide for a given precursor.

**Plasma proteome.** The procedure has been reported in the main Methods. We removed one sample that displayed low identification rates (**Supplementary Material** 2: Fig. S10), identified by MaxQuant software (Cox & Mann, 2008). From the iBAQ (intensity Based Absolute Quantification) intensity value, we then applied a similar procedure as described in the previous paragraph by first applying quantile normalization on the raw 324 protein group intensities. Second, we imputed missing values using similar thresholds as stated earlier before using the ComBat framework. At each step (prior to any processing, after normalization, after imputation and batch-effect correction), we represented the PCA in 3 dimensions of the samples (**Supplementary Material** 2: Fig. S11) and reported the percentage of variance explained by each component. We also performed Principal Component Variance Analysis (PVCA) without interaction (<https://github.com/dleelab/pvca> itself adapted from the pvca::*PVCA* R Bioconductor function (version 1.386.0; Bushel, 2022) in order to have an idea of the most prominent sources of variability in the intensities at each processing step assessing the contribution to the variance of the therapy status, the batch, as well as the individual effect and the residual variance selecting the PCs that contribute to an amount of variation larger than 80%.

**Statistical analyses of post-translational modification (PTM) and plasma biomarkers of sport therapy**

**Biomarker discovery.** For each dataset (buccal cells, PBMCs, and plasma), we evaluated candidate markers using three testing procedures: i) paired Wilcoxon test using the *wilcox.test* R function (paired=T), ii) paired t-test using the *t.test* R function (paired=T), as well as iii) a supervised model, partial-least square discriminant analysis (PLS-DA) using the mixOmics::*splsda* R function ( version 6.220.0; Rohart et al., 2017; scale=T, ncomp=1 (plasma) or 2, multilevel=participants ID). The tests were applied on the processed log2-intensities (plasma proteins) or the arcsin-square root percentage (PTMs) and corrected separately for multiple testing using both Benjamini-Hochberg and Benjamini-Yekutieli procedures – per precursor for epigenetic data. From the PLS-DA, we extracted the variable importance score (VIP) of each marker using the mixOmics::*vip* R function and reported its 95% confidence interval obtained by Jackknife. To obtain a reliable list of markers, we intersected candidate markers from the classic tests (candidate markers being significant at α=0.05) and the discriminant analysis (candidate markers whose VIP´s confidence interval lower bound is equal or above 1) leading to a total of 41 protein markers (**Supplementary Material** 2: Fig. S12) and 6 (+2 unmodified) and 11 (+1 precursor) for swab and PBMC epigenetic markers (**Supplementary Material** 2: Fig. S7) respectively. We eventually reported the average log2-fold change (plasma proteins) or the average percentage of change (PTMs) after the therapy relative to before the therapy over the paired individuals as a final estimate of each marker´s effect size.

**Data interpretation**

**Performance and Backpain self-assessment classes.** To better understand the clustering of the participants into different response categories, we performed **Multiple Factor Analysis (MFA) using the COVID status and both classes´ categories (“moderate”, “intermediate”, and “max”, and** “Unimpaired”, “Improving”, “Stagnating”, and “Worsening”, for each class respectively) **as illustrative variables and the following sets of variables as active qualitative variables: 1) socio-demographic environment (association of backpain with work, work position, relationship status and study level, 4 variables), 2) lifestyle (Exercise and Dietary habits classes, 2 variables) and 3) Backpain history (last 12 months, last 3 months and at entry, 3 variables). We used the FactoMineR::*MFA* R function** (version 2.97; Lê et al., 2008) **and reported intermediate plots useful for the axes´ interpretation as well as individual factor map (Supplementary Material file 2: Figs. S4-5) using factoextra::*fviz_mfa_ind* R function** (version 1.0.7; Kassambara & Mundt, 2020)**.**

**Histone PTM analysis. Since swab and PBMC sport therapy markers do not overlap but do not cluster by dataset (Fig. 4A), we checked if they could reflect complementary information on (latent) groups of individuals, which would either match backpain background, response to therapy or lifestyle components. From the individual percentage of change of each 17 PTM markers (6 from the swab and 11 from PBMC), we built two similarity networks, one per epigenetic dataset, using** the SNFtool::*affinityMatrix* R function (version 2.3.1; Wang et al., 2021; K=2 nearest neighbors) from the pairwise Euclidian distance matrix**, then integrated using the SNFtool::*SNF* R function. Clusters from the combined matrices were obtained from the** SNFtool::***spectralClustering* R function and represented using the** SNFtool::***displayClustersWithHeatmap* R function (Fig. 4B, Supplementary Material 2: Fig. S8). To give sense to these clusters, we performed an MFA using the three spectral clusters as illustrative variables and the following sets of variables as active qualitative variables: 1) lifestyle (Exercise and Dietary habits classes, 2 variables), 2) Backpain history (last 12 months, last 3 months and at entry, 3 variables) and 3) therapy evaluation (Performance and Self-assessment classes) (Fig. 4C and D). We used the FactoMineR::*MFA* R function and reported intermediate plots useful for the axes´ interpretation as well as individual factor map (Supplementary Material file 2: Fig. S9) using factoextra::*fviz_mfa_ind* R function.**

**Plasma proteome analysis. We represented the intensity values of the 41 plasma proteome biomarkers (rows) using the ComplexHeatmap::*Heatmap* R function** (version 2.182.01; Gu et al., 2016) **across paired and unpaired samples (columns) to check if the unsupervised clustering of the samples resulted in their labeling, *i.e.,* before or after therapy sampling time. Though the leading protein was represented as row names (Fig. 5A), we queried all proteins within the protein groups using the UniProt.ws Bioconductor package** (version 2.4236.05; Carlson & Maintainer, 2022)**. Two clusters displaying average opposite fold change (negative and positive) were then labeled using annotation word clouds from Uniprot keywords with the simplifyEnrichment::***a****nno_word_cloud* R function** (version 1.126.01; Gu & Huebschmann, 2021) **allowing a maximum of 10 words after having checked for unique terms, singularized the annotations and removed non-pertinent connectors and adverbs.**

**Additionally, we zoomed on subsets of protein groups markers whose intensities displayed either a linear trend or plateau over the three Performance categories and three Backpain self-assessment categories pertinent for the therapy evaluation (**“Improving”, “Stagnating”, and “Worsening”**). Per class of response, markers´ intensities were first clustered based on the trend of their log2-fold change (LFC) across the three categories using the tscR::*slopeDist* and tscR::** ***getClusters* R functions** (version 1.108.0; Pérez-Sanz & Riquelme-Pérez, 2022)**, which respectively computed the pairwise slope distance matrix between markers and performed clustering over 13 clusters – representing all possible trend shapes over 3 categories. Second, to label and to retain the clusters of interest – displaying either a linear trend or plateau over the two most similar categories (Fig. 5B and C)-, we used the segmented::*segmented* R function** (version 2.0.31.6-2; Muggeo et al& others, 2008) **to assess the presence of a breakpoint with the following arguments: psi=2, fixed.psi=2, npsi=1, seg.control(it.max=0). We applied the Davies test to test for linear trend using the segmented::*davies.test* R function at relaxed** α**=0.1 after having applied the Benjamini-Hochberg procedure. Per marker, a plateau was then defined from i) a significant Davies p-value indicating different slopes before and after the breakpoint, ii) a ratio of max/min slope >1.5 and iii) a minimum slope of 0.7. We proceeded to a similar annotation as described earlier and centered intensities using either the plateau average (plateau trends) or the average intensity (linear trends), normalized then between 0 and 1 (Fig. 5D and E).**

**Data integration of PTM and plasma biomarkers**. Since histone modifications can act as repressive or activating marks, we aimed to identify correlated signatures of therapy response across different sources of biological samples by using DIABLO (Data Integration Analysis for Biomarker discovery using Latent variable approaches for Omics studies, (Singh et al, 201935)). We applied DIABLO on arcsin-squared-root transformed epigenetic relative abundance (two data blocks) and proteomic log2-intensities (one data block) of the previously identified therapy markers of paired participants before and after therapy. Thus we scanned for correlated changes upon therapy across datasets using the mixOmics::*block.splsda* R function (with a partially fully connected design matrix between blocks, off-diagonal element set to 0.75 and two components). We then retained the strongest correlations (threshold of 0.7) between markers´ changes originating from different sources, *i.e.,* between PTMs and proteins but neither within proteins nor within PBMC PTMs for example, using the mixOmics::*circosPlot* R function (Fig. 6A). Correlations were transformed as a graph using the igraph::*graph_from_data_frame* R function (version 2.0.1.11.3.5; Csardi & Nepusz, 2006) and represented with the ggraph R package (version 2.1.0; Pedersen, 2022), encoded as red (positive) and blue (negative) vertices.

Because the 7 PTMs may display correlation with proteins involved in the organism´s functions, we additionally investigated the distribution of their percentage of change upon therapy over the Performance and Self-assessment categories. To that aim, we performed a Principal Component Analysis (PCA) of the individual percentage of change of these 7 PTM, where each PTM had a position in the 2D-PC plane (Fig. 6B). The location within the plane represented different trends over both Performance and Self-assessment categories (Fig. 6B, Supplementary Material 2: Fig. S14).

To obtain a higher level of information about PTM-plasma protein interactions, we annotated the 26 protein groups connected at least once with the 5 PBMC- and 2 swab-originating marker PTMs with GO BP terms using the Uniprot database, similarly as described in the **”Plasma proteome analysis”** sub-section. GO BP terms with occurrence was >2 were retained and annotated by their parental terms (Supplementary Material 2: Fig. S15) using consecutively the rrvgo::*calculateSimMatrix* (version 1.108.0; Sayols, 2020; ont="BP", method="Rel") and rrvgo::*reduceSimMatrix* R functions (medium similarity of 0.7) (Fig. 6C and D).

**Effects of therapy on health-awareness indicators across biological samples.** Per sample type (buccal cells, PBMC, plasma) and for three lifestyle components (Exercise, Dietary habits classes, and BMI – obese/overweight (BMI>29.9) or normal (BMI in ]24.9, 29.9])), we searched for discriminative markers of categories from the full epigenetic/proteomic profiles using the PLS-DA framework, implemented in the mixOmics::*splsda* R function (scale=T, ncomp=2 arguments). Because we obtained measurements before and after therapy, we performed two PLS-DAs, *i.e.,* repeated for BT or AT datasets. This led to a total of 3 (sample type) × 3 (lifestyle) × 2 (BT/AT) PLS-DA models. For a given sample type and lifestyle component combination, we extracted each PTM/protein VIP for BT and AT PLS-DAs using the mixOmics::*vip* R function and retained as health-awareness indicators the PTMs/proteins that satisfied a VIP threshold of 1 in both BT and AT PLS-DAs. Subsequently we computed two complementary metrics of therapy effect on a given lifestyle component and sample type.

(**1**) We first predicted the AT data variates of each participant from the fitted BT PLS-DA in 2D using the mixOmics::*predict* R function (dist=”mahalanobis.dist”) and then computed the Euclidian distance between BT and AT points, d_BT-AT_ (Fig. 7A, Supplementary Material 2: Fig. S16 and S17). This distance served as a proxy of the effect of the therapy and takes into account all weighted PTMs/proteins. We additionally examined the relationship between d_BT-AT_ and the Euclidian distance of each BT point to the center of gravity of the healthier group of participants in the 2D-variate map (Supplementary Material 2: Fig. S18). We fitted a linear model using the MASS::*rlm* R function ( version 7.3.-58.2; Venables & Ripley, 2002) by robust regression using an M estimator, and reported the corresponding p-value from robust F-test obtained from the sfsmisc::f.robftest R function (version 1.1.17-13; Maechler, 2022) (Supplementary Material 2: Fig. S18).

Note that we relied on the clustering obtained from the questionnaire data to define healthy groups of individuals before and after therapy and which are sportive (Exercise class), flexitarian/balanced diet (Dietary habits), and normal BMI categories. Per lifestyle component, we obtained three vectors of d_BT-AT_ distances (one per sample type) which pairwise concordance is quantified by Kendall’s W implemented in the irr::*kendall* R function (version 0.84.1; Gamer et al., 2010) (Fig. 7B, Supplementary Material 2: Fig. S19). Nevertheless, this distance is not oriented, *i.e.,* it does not indicate if the effect size reflects a change towards healthy or unhealthy state upon therapy.

(**2**) We thus measured the similarity of a given participant profile to the healthiest group of participants before and after therapy. For each lifestyle component and per sample type, we first recorded the number of VIP-based health-awareness indicators whose normalized values fell within the healthy range obtained by Jackknife, r_h_, and indicated if it improved before and after therapy (Fig. 7C, Supplementary Material 2: Fig. S20). Because this did not take into account measurement uncertainties, we additionally computed a weighted averaged similarity score that takes into account the distance of each health-awareness indicator to the healthy bounds. To that aim, we assigned a weight of 1 to health-awareness indicators´ values within the healthy range; 0 if the nearest distance d_min_ to either healthy bound (upper or lower) is bigger that 1/3 of the interval span r_h_, and a weight of 1-d_min_/(r_h_/3) otherwise (Supplementary Material 2: Fig. S21). We eventually performed paired t-test, *i.e.,* BT and AT, on these healthy similarity metrics and reported the Benjamini-Hochberg adjusted p-values (Fig. 7D).

**References**

Andri et mult. al., S. (2022). *{DescTools}: Tools for Descriptive Statistics*.

Biernacki, C., Celeux, G., & Govaert, G. (2000). Assessing a mixture model for clustering with the integrated completed likelihood. *IEEE Transactions on Pattern Analysis and Machine Intelligence*, *22*(7), 719–725.

Bolstad, B. (2022). *preprocessCore: A collection of pre-processing functions*.

Brunson, J. C., & Read, Q. D. (2023). *ggalluvial: Alluvial Plots in “ggplot2.”*

Bushel, P. (2022). *pvca: Principal Variance Component Analysis (PVCA)*.

Carlson, M., & Maintainer, B. P. (2022). *UniProt.ws: R Interface to UniProt Web Services*.

Commission, E. (2022). *Health promotion and disease prevention knowledge gateway*.

Cox, J., & Mann, M. (2008). MaxQuant enables high peptide identification rates, individualized ppb-range mass accuracies and proteome-wide protein quantification. *Nature Biotechnology*, *26*(12), 1367–1372.

Csardi, G., & Nepusz, T. (2006). The igraph software package for complex network research. *InterJournal*, *Complex Systems*, 1695.

Dempster, A. P., Laird, N. M., & Rubin, D. B. (1977). Maximum likelihood from incomplete data via the EM algorithm. *Journal of the Royal Statistical Society: Series B (Methodological)*, *39*(1), 1–22.

Gabadinho, A., Ritschard, G., Müller, N. S., & Studer, M. (2011). Analyzing and Visualizing State Sequences in R with TraMineR. *Journal of Statistical Software*, *40*(4 SE-Articles), 1–37. https://doi.org/10.18637/jss.v040.i04

Gamer, M., Lemon, J., & Singh, I. (2010). *irr: Various Coefficients of Interrater Reliability and Agreement*.

Gower, J. C. (1971). A general coefficient of similarity and some of its properties. *Biometrics*, 857–871.

Gu, Z., Eils, R., & Schlesner, M. (2016). Complex heatmaps reveal patterns and correlations in multidimensional genomic  data. *Bioinformatics (Oxford, England)*, *32*(18), 2847–2849. https://doi.org/10.1093/bioinformatics/btw313

Gu, Z., & Huebschmann, D. (2021). simplifyEnrichment: an R/Bioconductor package for Clustering and Visualizing Functional Enrichment Results. *Genomics, Proteomics & Bioinformatics*.

Kassambara, A., & Mundt, F. (2020). *Extract and Visualize the Results of Multivariate Data Analyses [R package factoextra version 1.0.7]*.

Kaufman, L., Rousseeuw, P. J., of Mathematics, F., & (Delft), I. (1987). *Clustering by Means of Medoids*. Faculty of Mathematics and Informatics.

Lê, S., Josse, J., & Husson, F. (2008). {FactoMineR}: A Package for Multivariate Analysis. *Journal of Statistical Software*, *25*(1), 1–18. https://doi.org/10.18637/jss.v025.i01

Leek, J. T., Johnson, W. E., Parker, H. S., Fertig, E. J., Jaffe, A. E., Zhang, Y., Storey, J. D., & Torres, L. C. (2022). *sva: Surrogate Variable Analysis*.

MacLean, B., Tomazela, D. M., Shulman, N., Chambers, M., Finney, G. L., Frewen, B., Kern, R., Tabb, D. L., Liebler, D. C., & MacCoss, M. J. (2010). Skyline: an open source document editor for creating and analyzing targeted  proteomics experiments. *Bioinformatics (Oxford, England)*, *26*(7), 966–968. https://doi.org/10.1093/bioinformatics/btq054

Maechler, M. (2022). *sfsmisc: Utilities from “Seminar fuer Statistik” ETH Zurich*.

Maechler, M., Rousseeuw, P., Struyf, A., Hubert, M., & Hornik, K. (2022). *cluster: Cluster Analysis Basics and Extensions*.

Muggeo, V. M. R., & others. (2008). Segmented: an R package to fit regression models with broken-line relationships. *R News*, *8*(1), 20–25.

Odegaard, A. O., Koh, W.-P., Arakawa, K., Yu, M. C., & Pereira, M. A. (2010). Soft drink and juice consumption and risk of physician-diagnosed incident type 2  diabetes: the Singapore Chinese Health Study. *American Journal of Epidemiology*, *171*(6), 701–708. https://doi.org/10.1093/aje/kwp452

Organization, W. H., & others. (2020). *WHO guidelines on physical activity and sedentary behaviour: web annex: evidence profiles*.

Pedersen, T. L. (2022). *ggraph: An Implementation of Grammar of Graphics for Graphs and Networks*.

Pérez-Sanz, F., & Riquelme-Pérez, M. (2022). *tscR: A time series clustering package combining slope and Frechet distances*.

R Core Team. (2022). *R: A Language and Environment for Statistical Computing*.

Raab, M. (2022). *ggseqplot: Render Sequence Plots using “ggplot2.”*

Revelle, W. (2022). *psych: Procedures for Psychological, Psychometric, and Personality Research*.

Rohart, F., Gautier, B., Singh, A., & Lê Cao, K.-A. (2017). mixOmics: An R package for ‘omics feature selection and multiple data integration. *PLOS Computational Biology*, *13*(11), e1005752.

Rousseeuw, P. J. (1987). Silhouettes: A graphical aid to the interpretation and validation of cluster analysis. *Journal of Computational and Applied Mathematics*, *20*, 53–65. https://doi.org/https://doi.org/10.1016/0377-0427(87)90125-7

Sayols, S. (2020). *rrvgo: a Bioconductor package to reduce and visualize Gene Ontology terms*.

Scrucca, L., Fop, M., Murphy, T. B., & Raftery, A. E. (2016). {mclust} 5: clustering, classification and density estimation using {G}aussian finite mixture models. *The {R} Journal*, *8*(1), 289–317.

Singh, A., Shannon, C. P., Gautier, B., Rohart, F., Vacher, M., Tebbutt, S. J., & Lê Cao, K.-A. (2019). DIABLO: an integrative approach for identifying key molecular drivers from  multi-omics assays. *Bioinformatics (Oxford, England)*, *35*(17), 3055–3062. https://doi.org/10.1093/bioinformatics/bty1054

Van den Ackerveken, P., Lobbens, A., Turatsinze, J.-V., Solis-Mezarino, V., Völker-Albert, M., Imhof, A., & Herzog, M. (2021). A novel proteomics approach to epigenetic profiling of circulating nucleosomes. *Scientific Reports*, *11*(1), 7256. https://doi.org/10.1038/s41598-021-86630-3

Venables, W. N., & Ripley, B. D. (2002). *Modern Applied Statistics with S* (Fourth). Springer.

Wang, B., Mezlini, A., Demir, F., Fiume, M., Tu, Z., Brudno, M., Haibe-Kains, B., & Goldenberg, A. (2021). *SNFtool: Similarity Network Fusion*.

Wei, T., & Simko, V. (2021). *R package “corrplot”: Visualization of a Correlation Matrix*.

Wickham, H. (2016). *ggplot2: Elegant Graphics for Data Analysis*. Springer-Verlag New York.

**
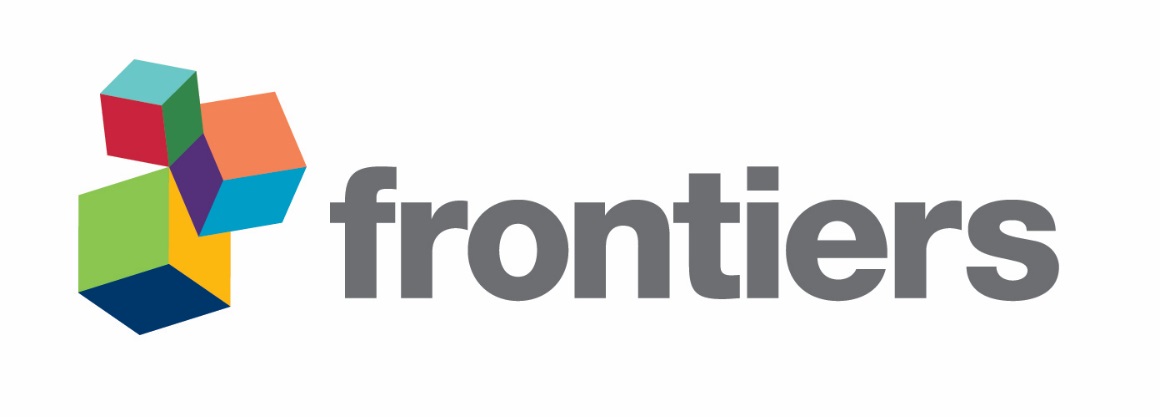
**
