# Supplementary material for: Back pain exercise therapy remodels human epigenetic profiles in buccal and human peripheral blood mononuclear cells: an exploratory study in young male participants
Source: Front Sports Act Living. 2024 Oct 16;6:1393067. doi: 10.3389/fspor.2024.1393067 (PMC11521823; doi:10.3389/fspor.2024.1393067)
Supplement: Supplementary file 2 [file Table2.docx]

Supplementary Material 2

**Backpain exercise therapy remodels human histone epigenetic profiles in buccal and human peripheral blood mononuclear cells**

**Claire Burny^†^, Mia Potočnjak^†^, Annika Hestermann, Sophie Gartemann, Michael Hollmann, Frank Schifferdecker-Hoch, Nina Markanovic, Simone Di Sanzo, Michael Günsel, Victor Solis-Mezzarino^†^, Moritz Voelker-Albert^†^***

† Equal contribution and first authorship

† Equal contribution and last authorship

*** Correspondence:** Moritz Voelker-Albert: moritz@moleqlar.de


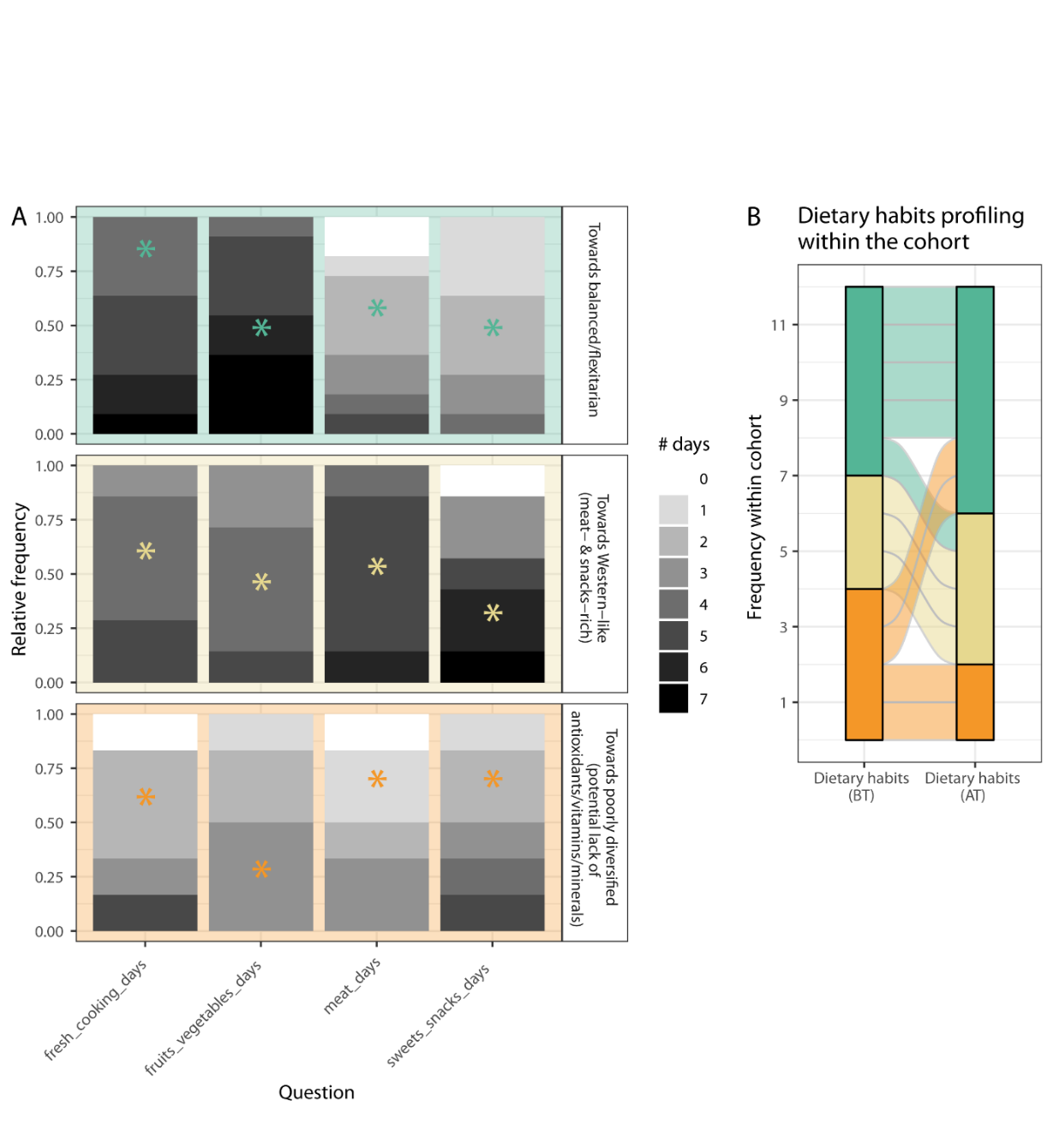


**Figure S1.** Representation of the Dietary habits class before and after therapy. (**A**) Stacked bar charts illustrating the answers´ (x-axis) distribution (y-axis) of all participants during the study. Per category, answer values are encoded from low to high (gray shade). Asterisks indicate the most representative answer of the cluster (medoids) and their color indicates the category label assigned to each cluster. (**B**) The alluvial plot displays changes in Dietary habits BT and AT (x-axis) per participant (alluvium, Friedman χ^2^ (1)=0.33, p=0.56). Categories are color-coded and described in the facets´ name in (**A**). All alluvial plots were generated using the ggalluvial R package (1).


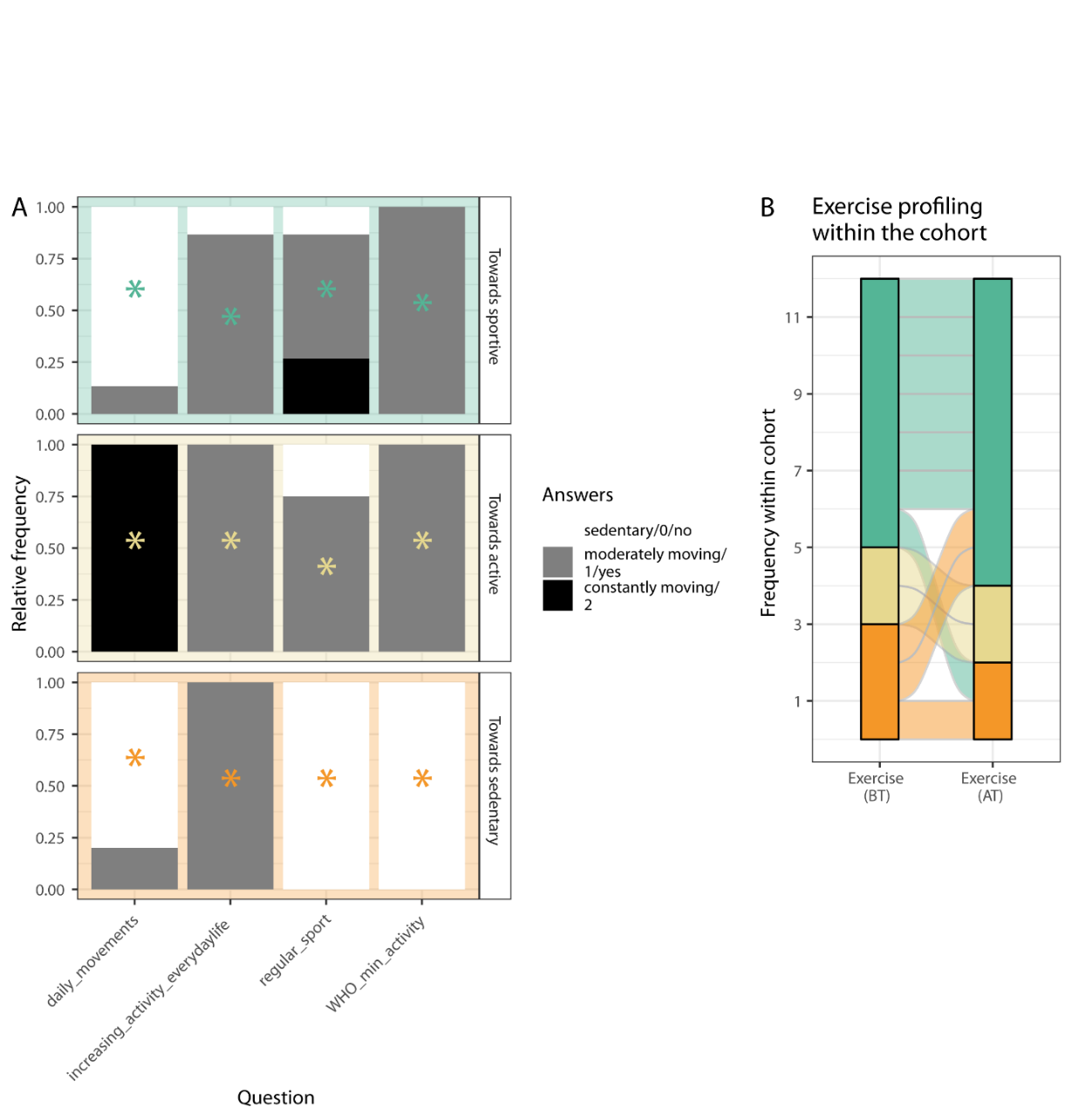


**Figure S2.** Representation of the Exercise class before and after therapy. (**A**) and (**B**) are similar as Fig. S1. The alluvial plot displays changes in Exercise habits BT and AT per participant (alluvium, Friedman χ^2^ (1)=0.33, p=0.56).


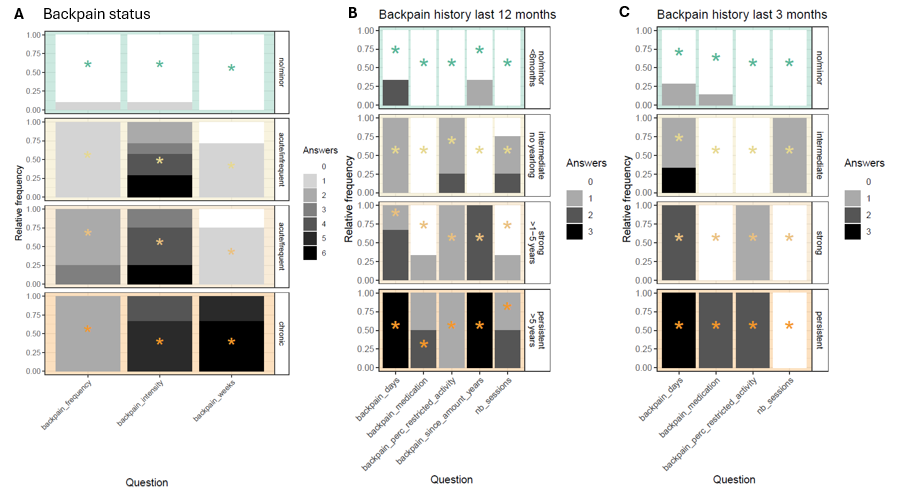


**Figure S3.** Stacked bar charts illustrating the answers´ (x-axis) distribution (y-axis) of all participants during the study similar as Fig. S1A. (**A**) Representation of the Backpain status answers. (**B**) and (**C**) Representation of the backpain history classes at 12 and 3 months respectively.


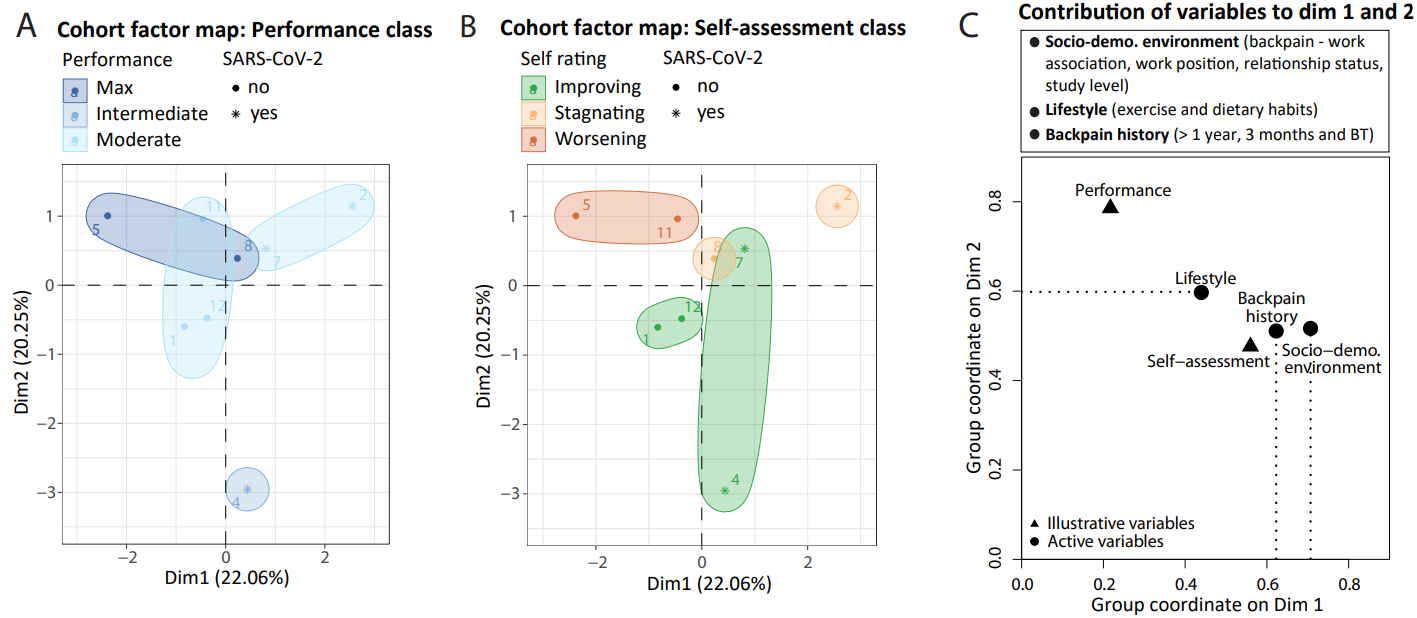


**Figure S4.** (**A, B**) Participants factor maps from Multiple Factor Analysis (MFA) performed from: 1) socio-demographic environment, 2) lifestyle and 3) backpain history groups of variables using Performance (**A**) and Self-assessment class (**B**) as illustrative variables (color-coded). COVID-19 infection during the therapy period is indicated as an asterisk. (**C**) The coordinates of the variables´ groups illustrate correlation with the first two MFA dimensions, which top (*i.e.*, above uniform) active (round-shaped) contributors are represented by dotted lines per dimension.


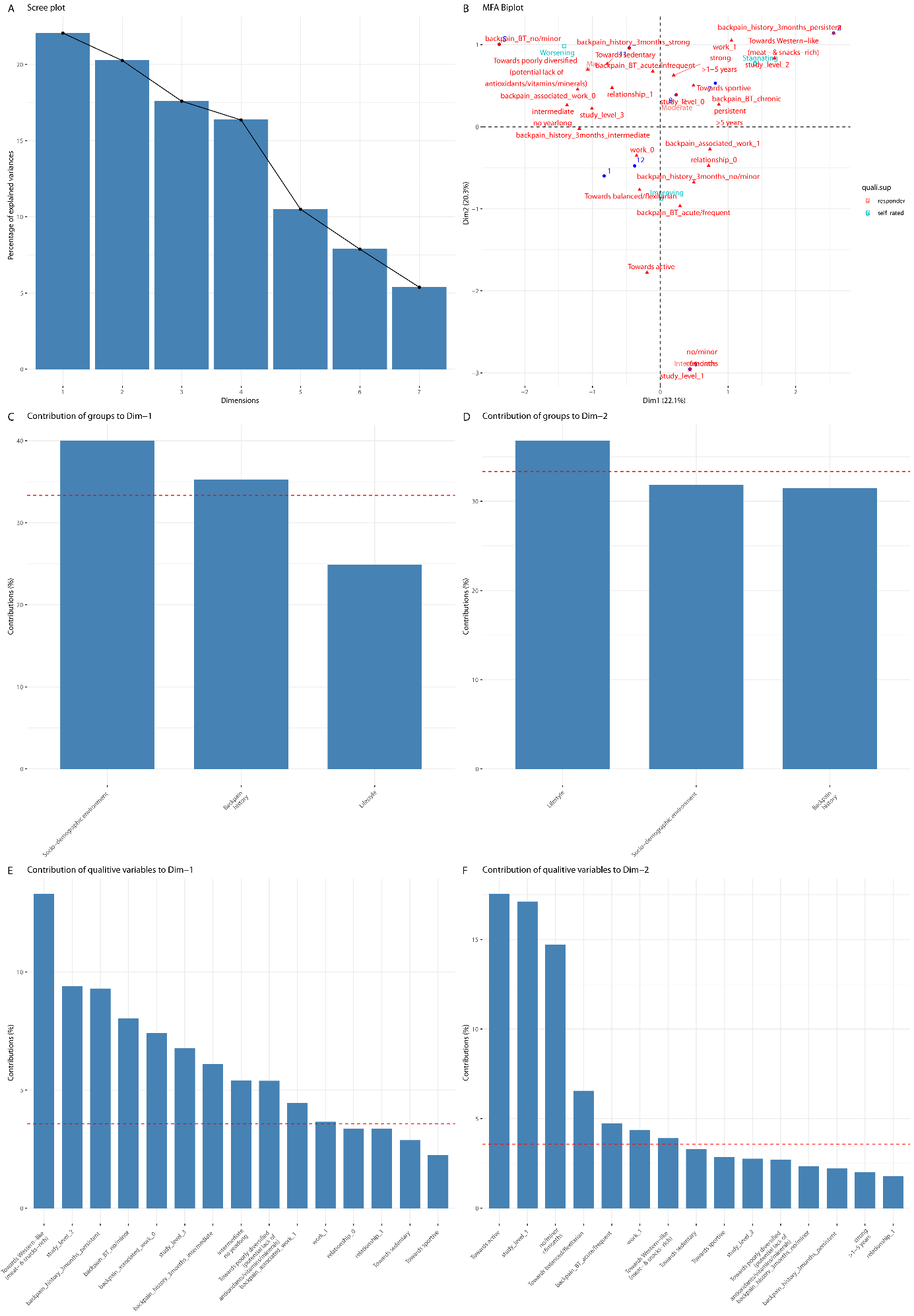


**Figure S5.** Quality control plots of the Multiple Factor Analysis illustrating Self-assessment and Performance classes. (**A**) Scree plot of the percentage of variance explained (y-axis) by each dimension (x-axis). (**B**) Biplot representing the position of each participant (blue dot), and each variable contributing to the 1) socio-demographic environment, 2) lifestyle, and 3) backpain history groups of variables (red triangle). Illustrative qualitative variables are represented by a square in turquoise (backpain Self-assessment categories) or pink (Performance categories). (**C**) and (**D**) barplots represent the relative contributions in percentages of each group of variables to dimensions 1 (**C**) and 2 (**D**) (y-axis). (**E**) and (**F**) barplots represent the relative contribution in percentages of each variable to dimensions 1 (**E**) and 2 (**F**). The red horizontal dotted lines indicate an expected uniform contribution. All plots have been done from the factoextra R package (2).


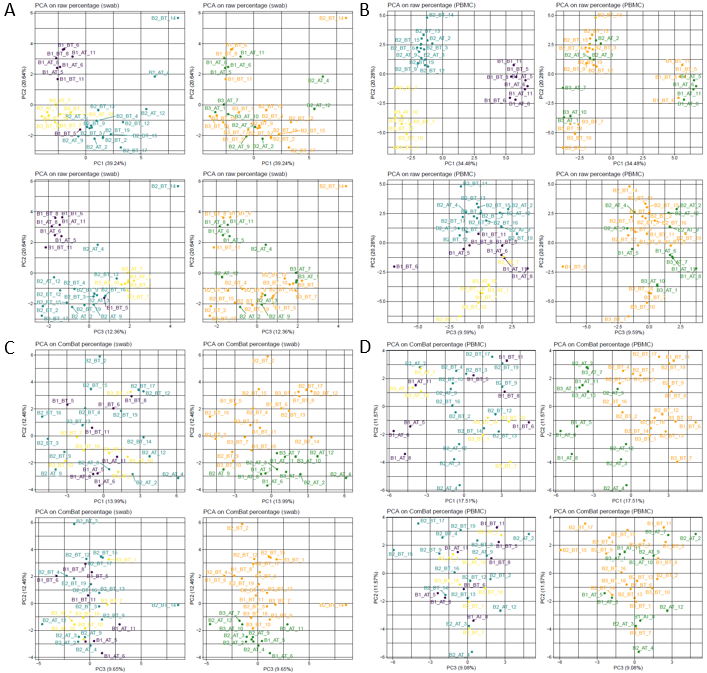


**Figure S6.** Quality control PCA plots of samples from buccal cells (**A**, **C**) and PBMC (**B**, **D**) before (**A**, **B**) and after (**C**, **D**) post-processing. Per panel, the first/second column encodes samples (*i.e.* dots) by /therapy status (before or after therapy), whereas the first row represents the first two dimensions and the second row, the second and third dimensions with the percentage of variance explained by component reported in the axis’s labels. After normalization, imputation and batch-effect correction, the samples are grouped less by batch, rather by therapy status, validating our post-processing procedures.


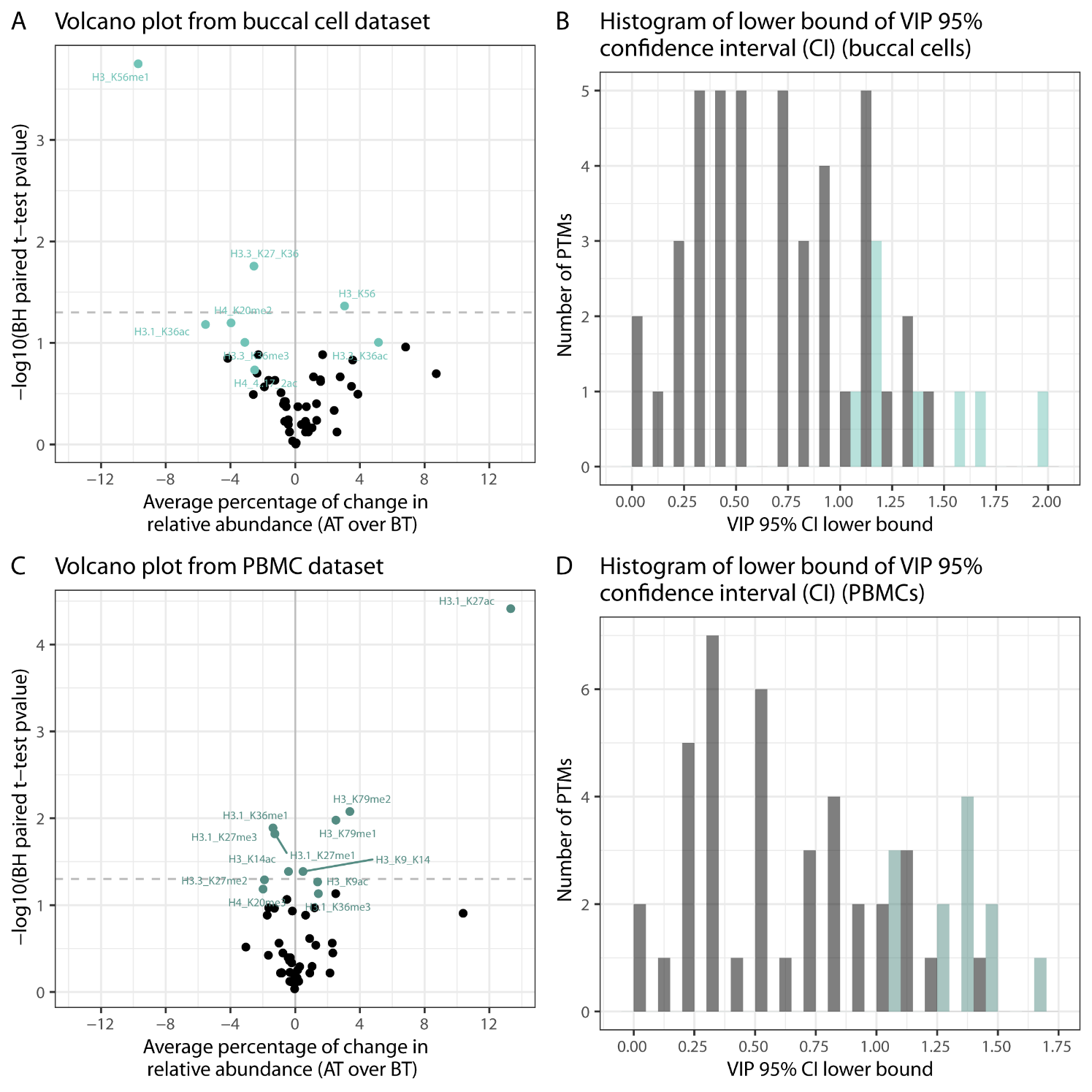


**Figure S7.** Volcano plots (**A**, **C**), and VIP distributions (**B**, **D**) for the buccal cell (top) and PBMC (bottom) datasets. In panels (**A**) and (**C**), one dot represents one histone PTM positioned according to its -log10 Benjamini-Hochberg adjusted p-value from the paired t-test (y-axis) and its average percentage of change in relative abundance over paired participants (x-axis). The horizontal dotted line in corresponds to α=0.05. The histograms (**B**) and (**D**) represent the distribution of the lower bound of the VIP 95% CI from the PLS-DA. The green colors encodes the final list of markers from the buccal cells (light turquoise) and PBMCs (dark turquoise).


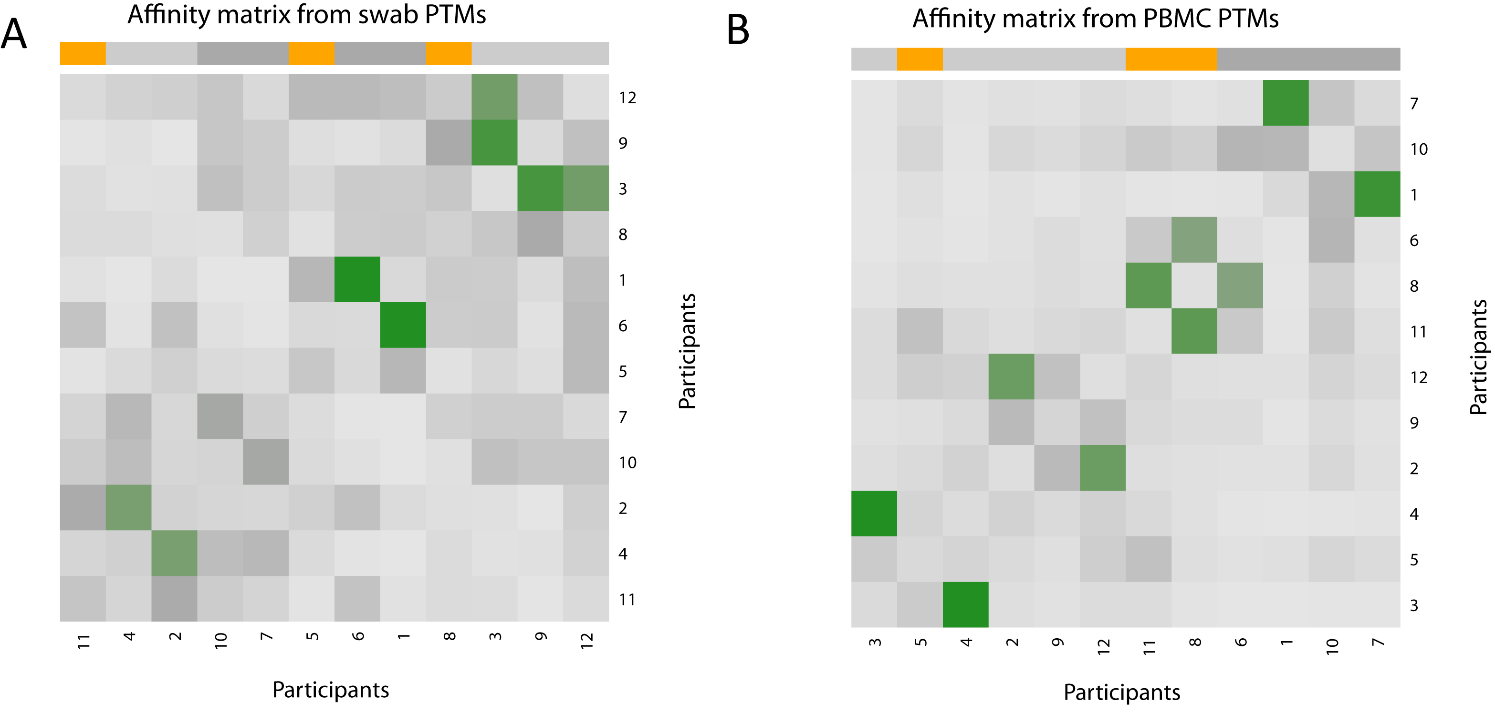


**Figure S8.** Pairwise participants similarity matrices from buccal cell and PBMC markers´ effect size before applying the Similarity Network Fusion algorithm (Fig. 4B). Colored cell indicates increased similarity, from gray to green. Spectral clusters membership is indicated in gray or orange shade.


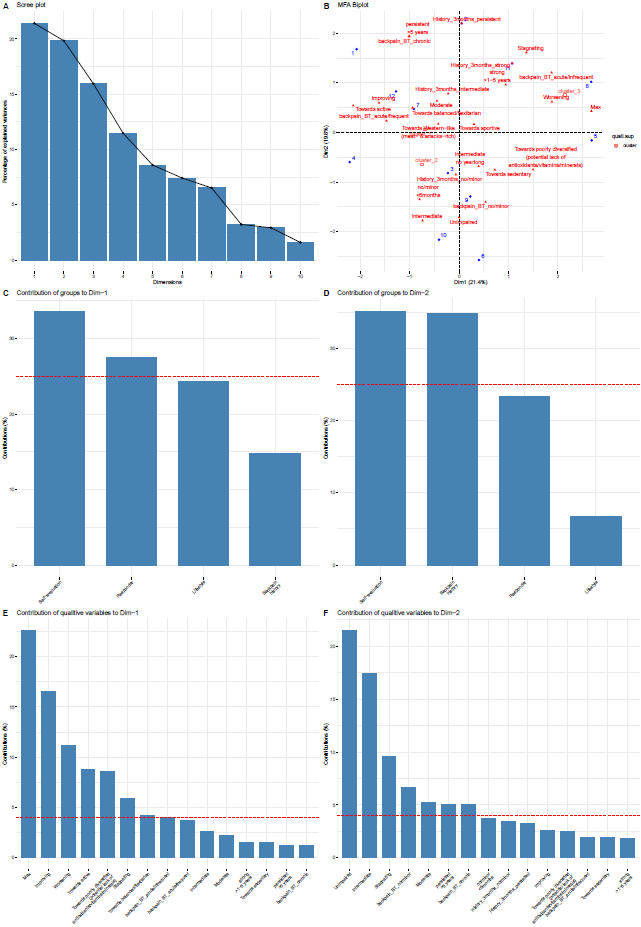


**Figure S9.** Quality control plots of the Multiple Factor Analysis illustrating spectral clusters (Fig. 4B). See Fig. S5 for a description of the panels.


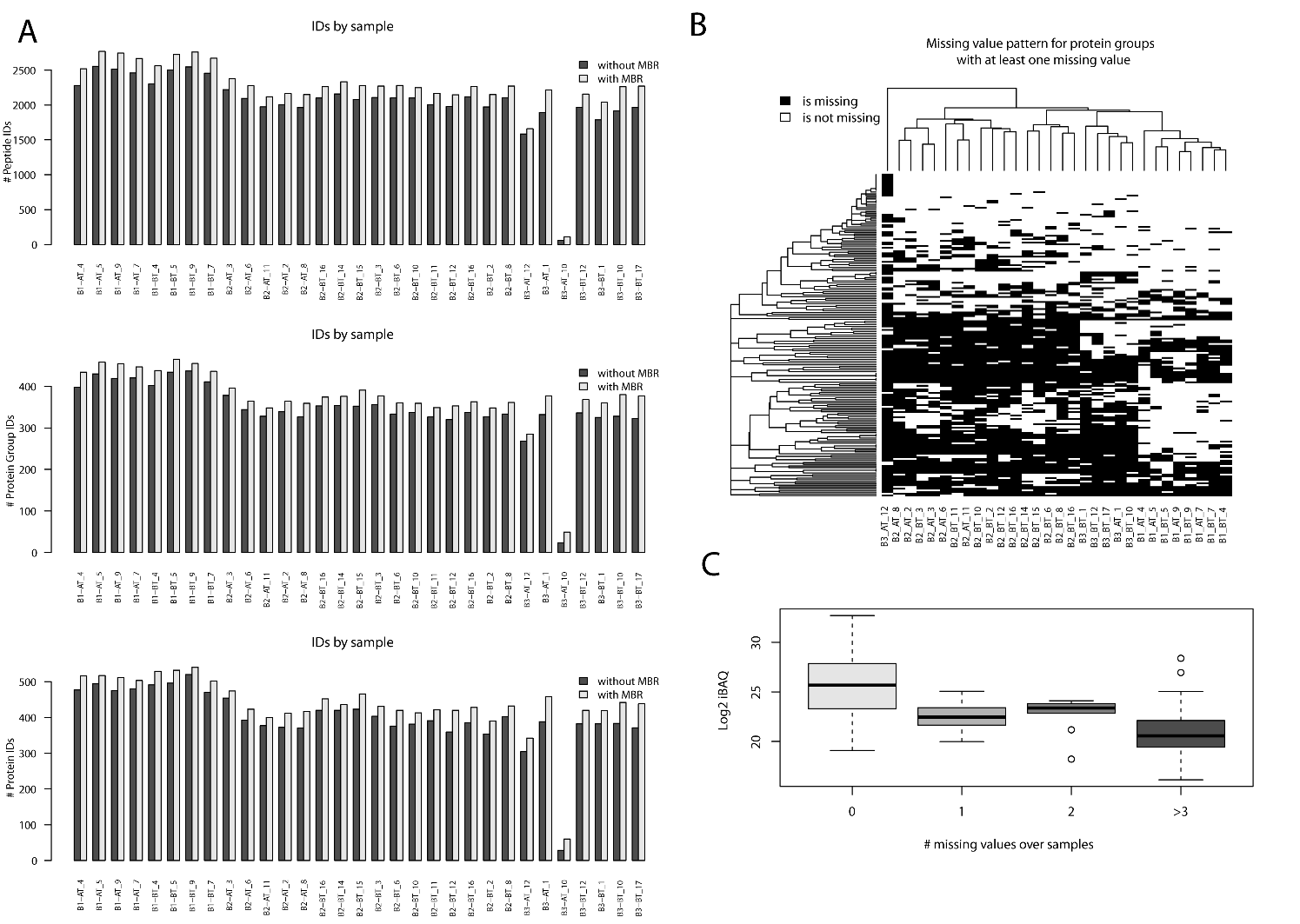


**Figure S10.** Quality control plots of MaxQuant raw output from plasma samples. (**A**) Barplots of the identification rates (y-axis) at the peptide, protein group and protein level from top to bottom, with and without match-between-run (MBR, light or dark gray bars). The therapy status (BT or AT) and the batch are encoded in the label of the x-axis. (**B**) Heatmap representing the unsupervised hierarchical clustering of samples´ (columns) missing value pattern across protein groups (rows) which display at least one missing value (black cell) using the binary distance. This heatmap reflects the presence of both types of missingness – Missing At Random and Not Missing At Random. (**C**) Boxplot illustrating the relationship between the average log2-intensities (y-axis) and the number of missing values over samples (x-axis).


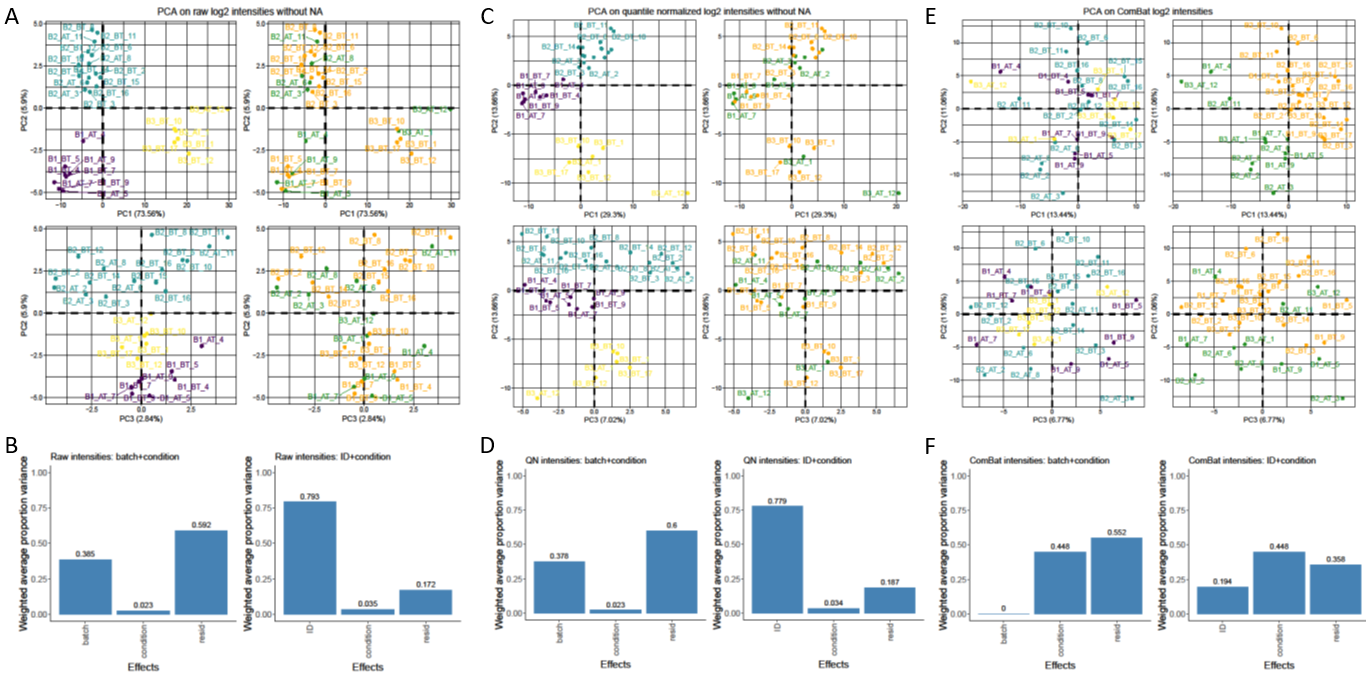


**Figure S11.** Quality control PCA plots from raw (**A, B**), quantile-normalized (**C, D**), and after post-processed (**E, F**) plasma samples. The legend of the 2D PC planes (**A, C, E**) is the same as Fig. S7. After normalization and batch-effect correction, the samples are grouped less by batch, rather by therapy status, validating our post-processing procedures, which is reflected by the univariate Principal Variance Component Analysis (PVCA) outcomes with an increased proportion of variance explained by the therapy status (condition) from (**B**), to (**D**), and (**F**), compared to the batch variable (barplots´ height, x-axis labels). PVCA analysis has been drawn from <https://github.com/dleelab/pvca>, itself adapted from the pvca::*PVCA* R Bioconductor function (3).

**
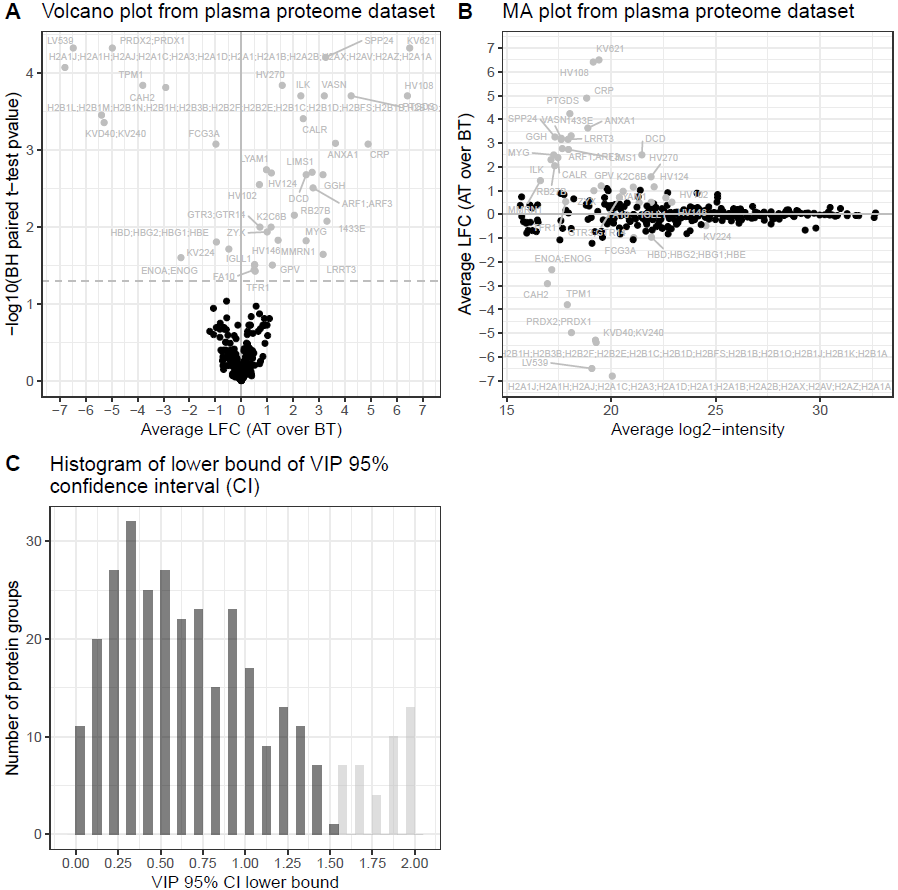
**

**Figure S12**. Volcano plot (**A**), MA plot (**B**), and VIP distribution (**C**) for the plasma proteome dataset. In panels (**A**) and (**B**), one dot represents one protein group either positioned according to its -log10 Benjamini-Hochberg adjusted p-value from the paired t-test (y-axis) and its average log2-fold change over paired participants (x-axis) in (**A**), or its average log2-fold change over paired participants (y-axis) versus its average abundance between BT and AT status (x-axis) in (**B**). The horizontal dotted line in (**A**) corresponds to α=0.05. The histogram (**C**) represents the distribution of the lower bound of the VIP 95% CI from the PLS-DA. The gray color encodes the final list of markers.


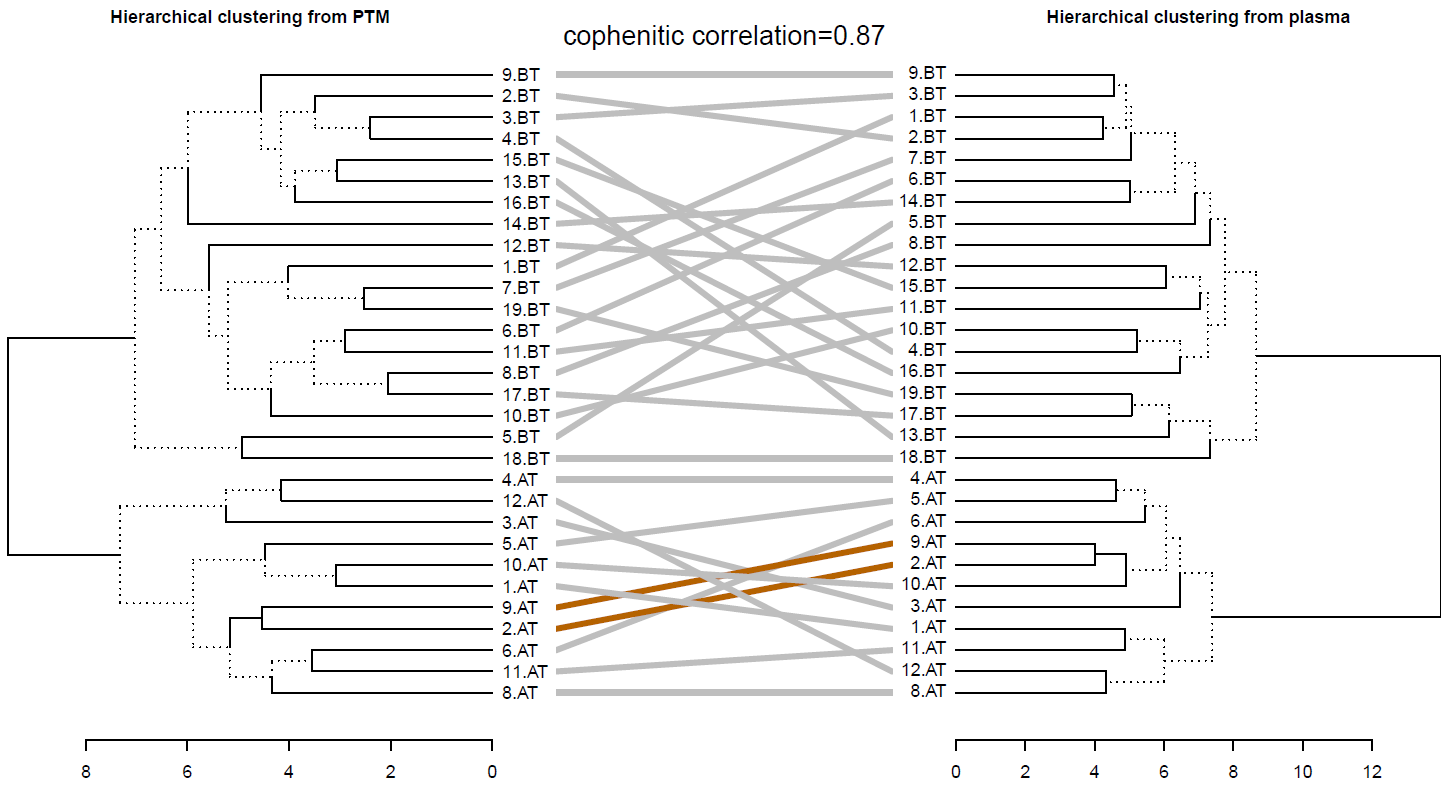


**Figure S13**. Tanglegram of hierarchical clustering from the PTM (left) and the proteome (right) for most informative markers, derived from the clustering of Fig. 4A and Fig. 5A respectively. Distinct edges are highlighted in brown. The cophenitic correlation is reported in the title.


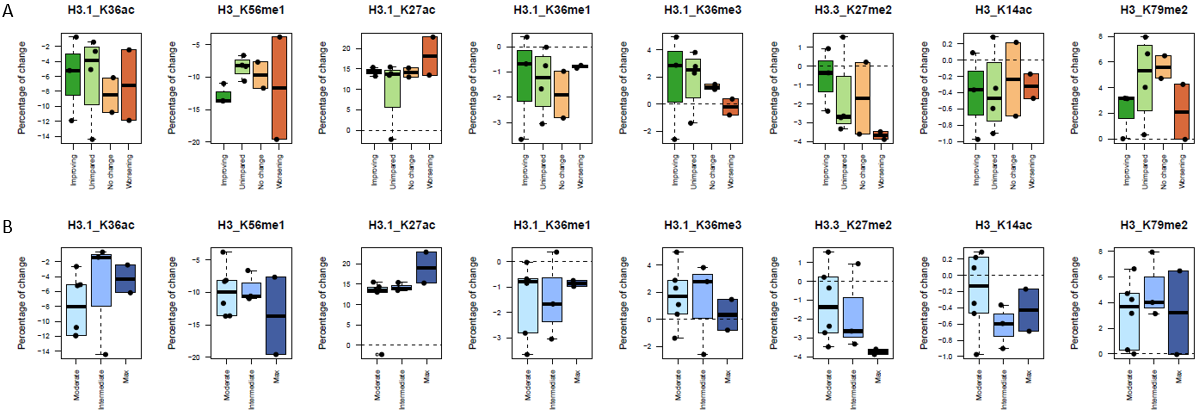


**Figure S14.** Trends of top DIABLO-connected PTM markers´ individual effect size over response classes. Boxplots of the percentage of change of relative abundance (y-axis) of the 8 PTMs (column) displaying at least one strong correlation with a protein marker´s change from DIABLO analysis over Self-assessment (**A**) and Performance (**B**) categories (x-axis). One dot represents one participant.


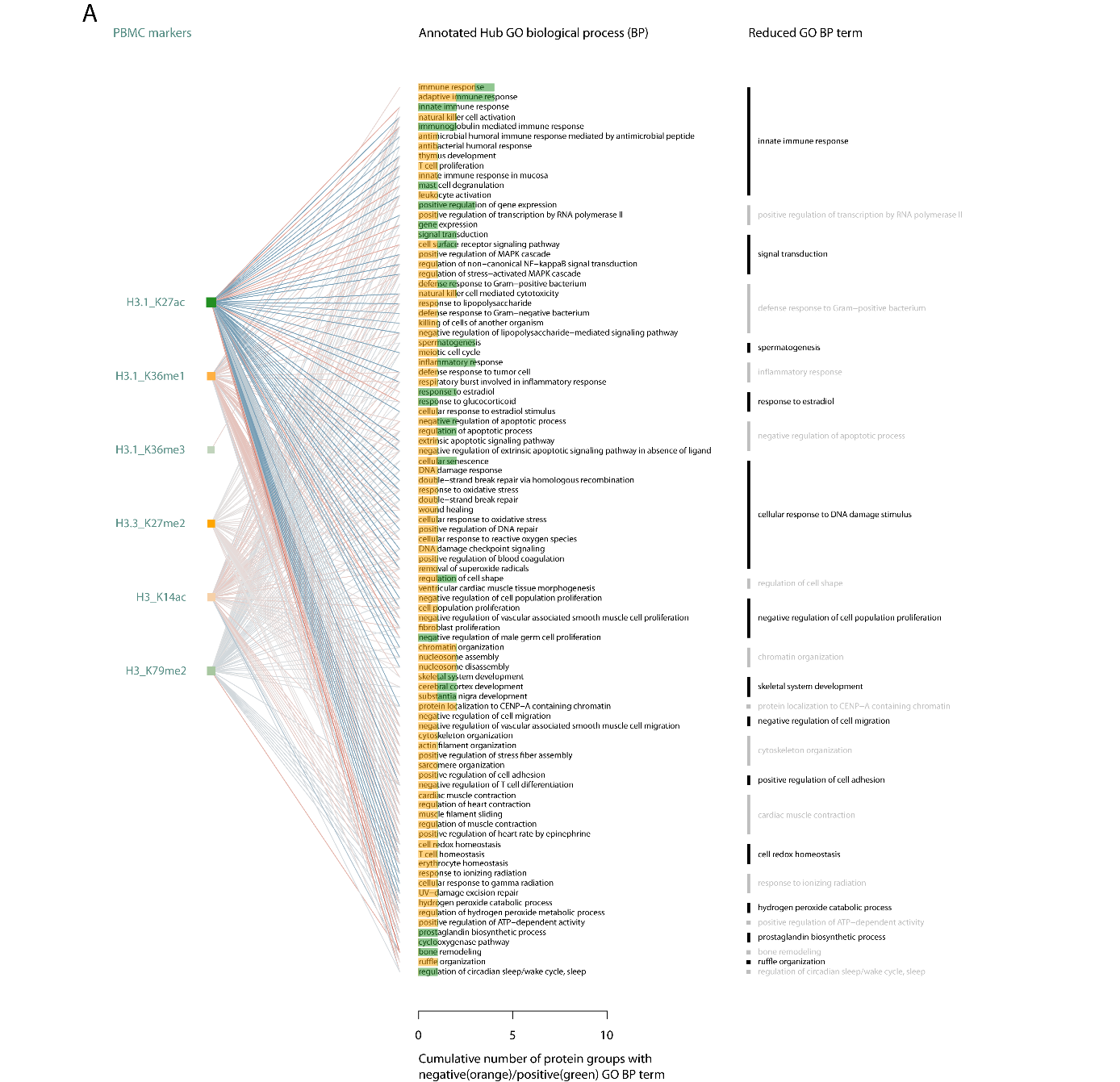


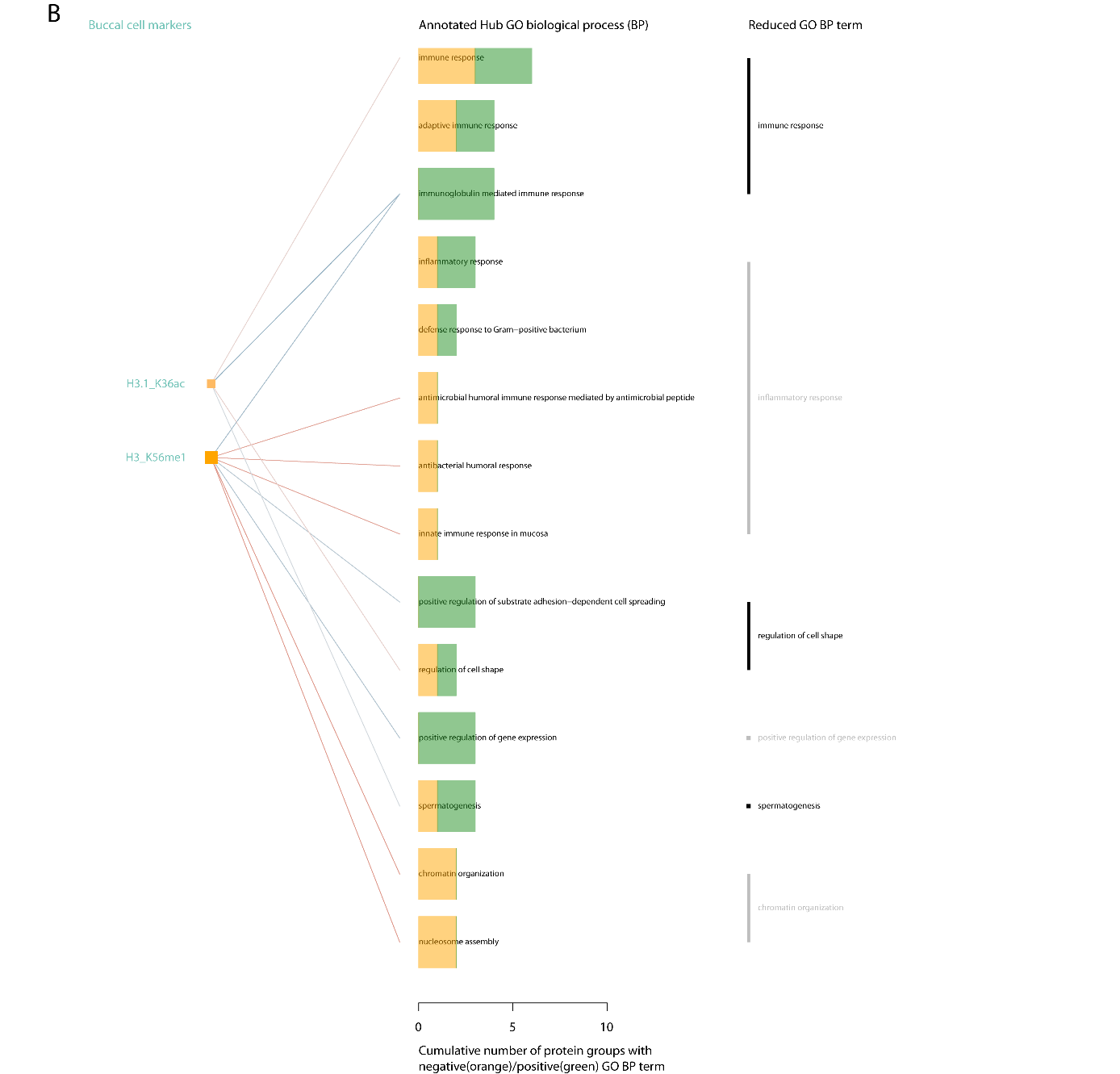


**Figure S15.** Detailed GO biological process (BP) annotation of the 27 protein markers highlighted by the functional DIABLO analysis separately for PBMC (**C**) and buccal cell (**D**) markers. GO terms whose annotated GO BP terms match 3 or more connexions were considered (y-axis) and ranked by their corresponding number of annotated protein groups (x-axis).


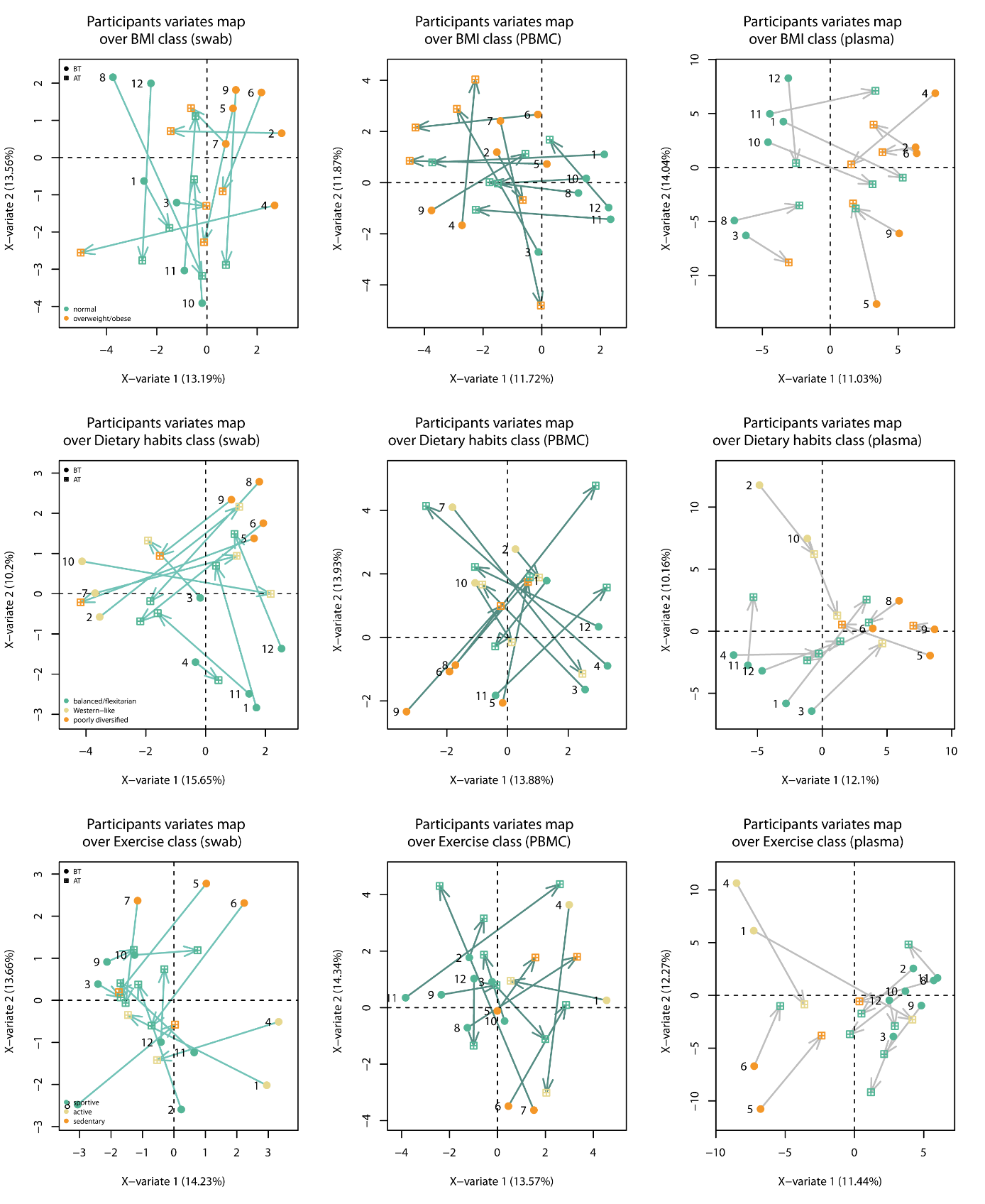


**Figure S16.** 2D-variate map of the PLS-DA performed from all molecular profiles (columns) using BMI and lifestyle categories (rows) as an outcome. Categories are color-coded and detailed in the legend. A participant consists of two points: initial BT position (round-shaped), and AT position (square-shaped) after the projection of his AT profile in the variate map, connected by an arrow.


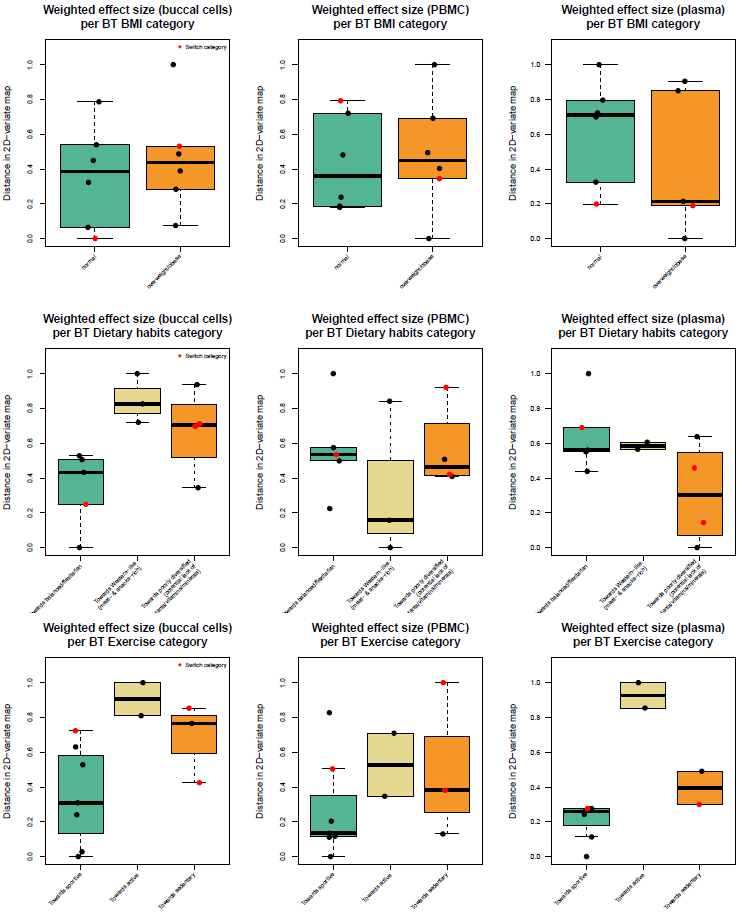


**Figure S17.** Boxplots representing the therapy effect size (y-axis) across sample types (columns) and BT BMI and lifestyle categories (rows, x-axis). One dot represents one participant, colored in red when the participant switched categories upon therapy.


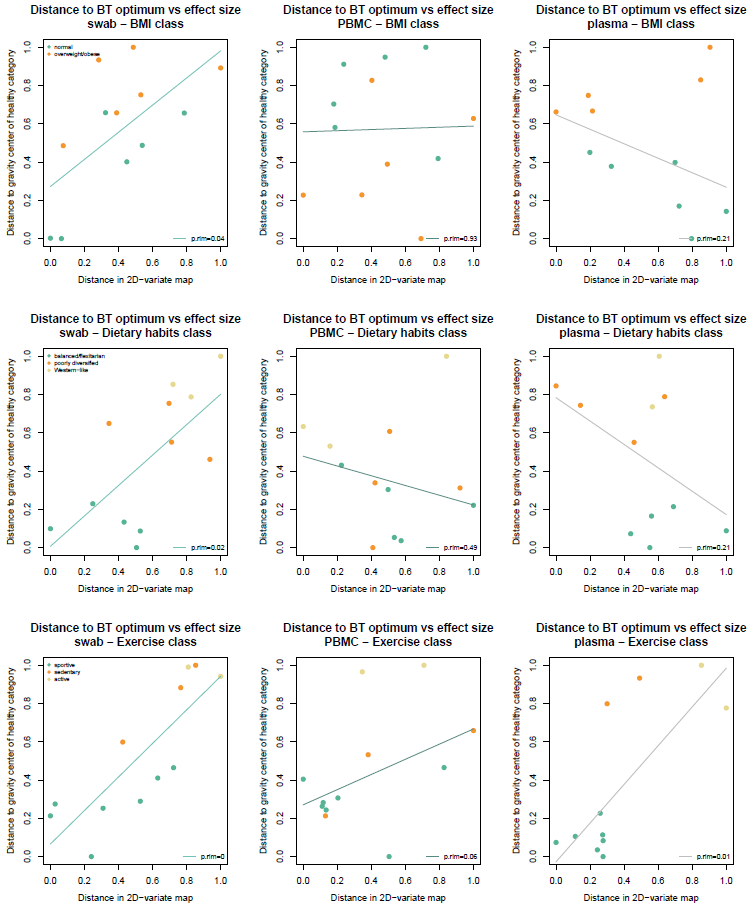


**Figure S18.** Scatterplots of the therapy effect size (x-axis) versus the distance to the healthier category (y-axis) in the 2D-variate map across sample types (columns) and BT BMI and lifestyle categories (rows). Per panel, the line represents the outcome of a linear model by robust regression using an M estimator, where p-value has been obtained from a robust F-test using both MASS (4) and sfsmisc (5) R packages.


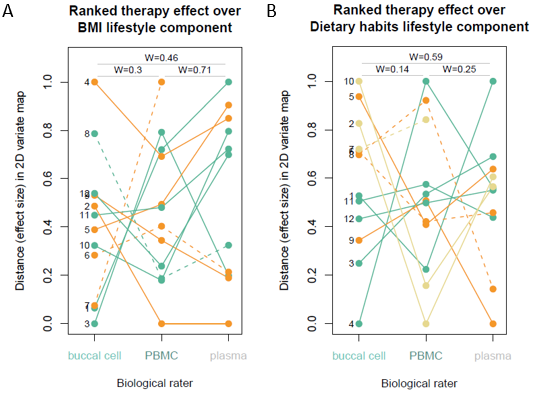


**Figure S19.** Bump charts representing the participants´ ranks (y-axis) from increased therapy effect size (*i.e.*, arrow length in Fig. S16) across sample types (x-axis) from BMI (**A**) and Dietary habits (**B**) categories. Connecting lines are colored based on the respective BT category. Dashed line indicates that the participant switched categories upon therapy. Pairwise concordance Kendall´s values are reported across biological raters.


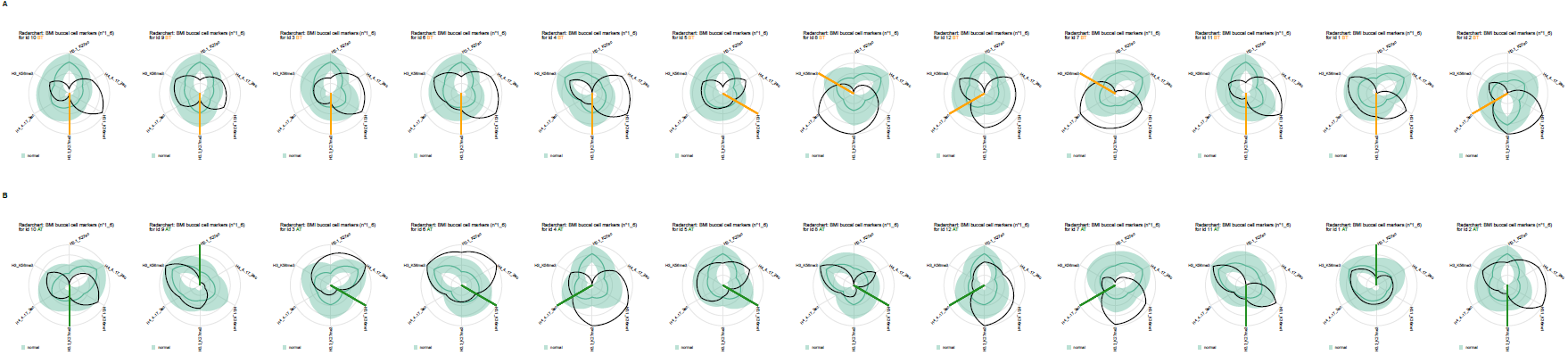


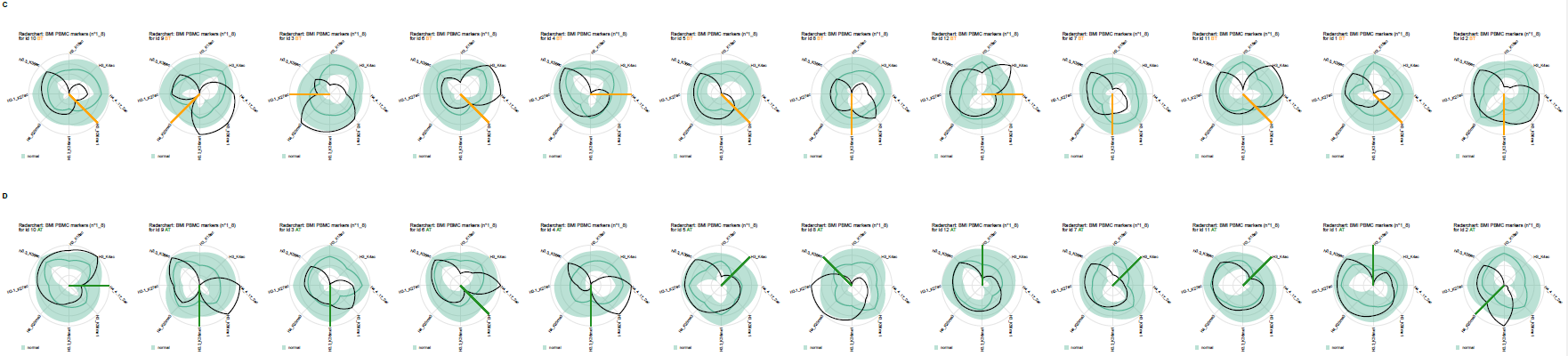


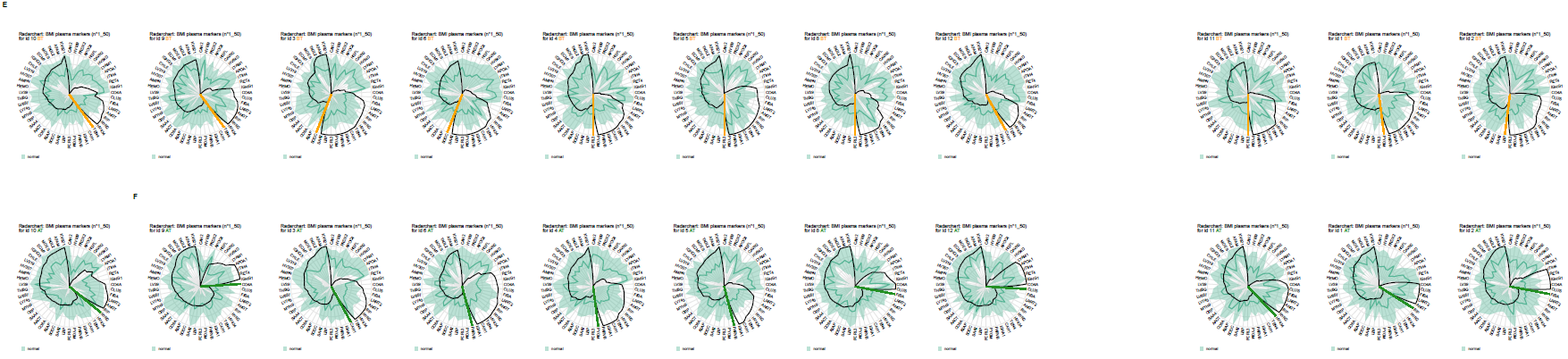


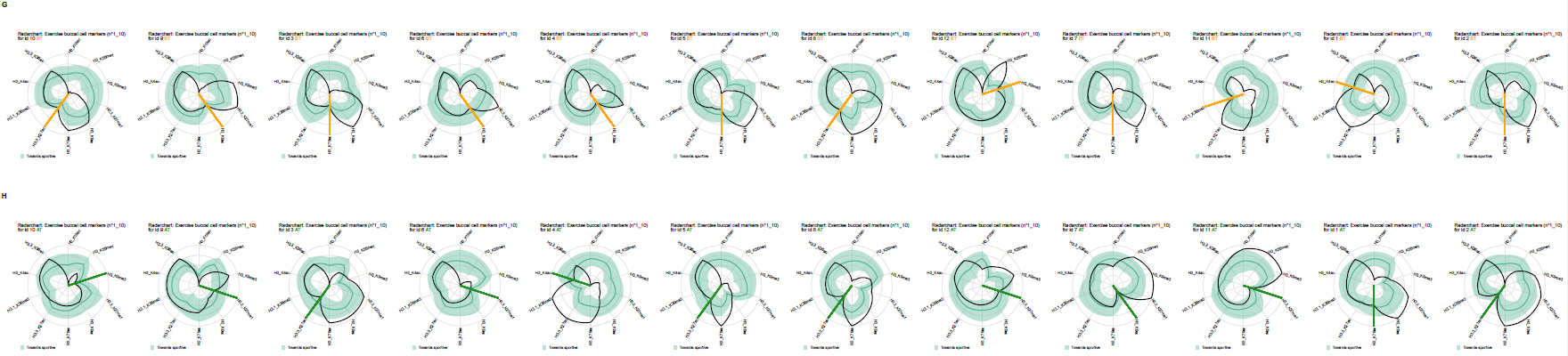


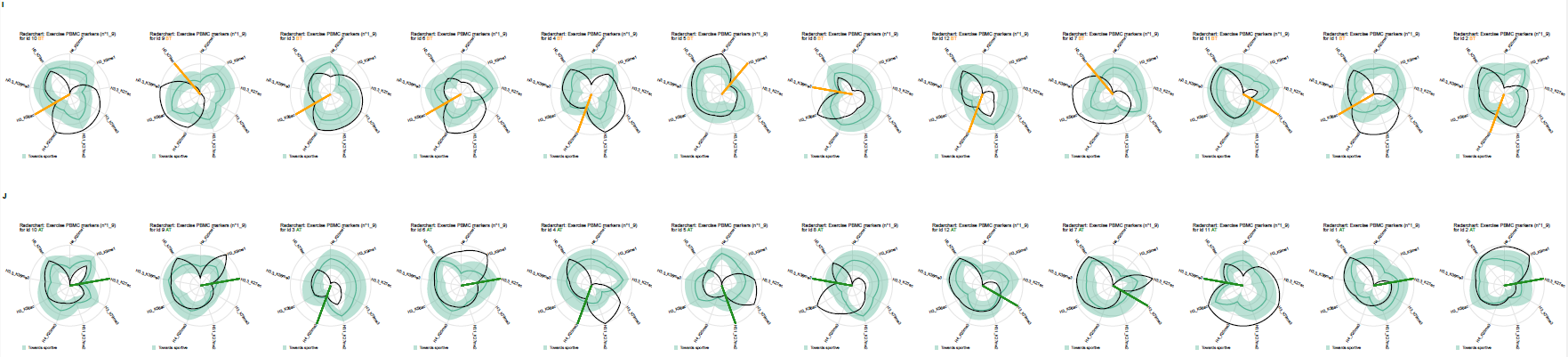


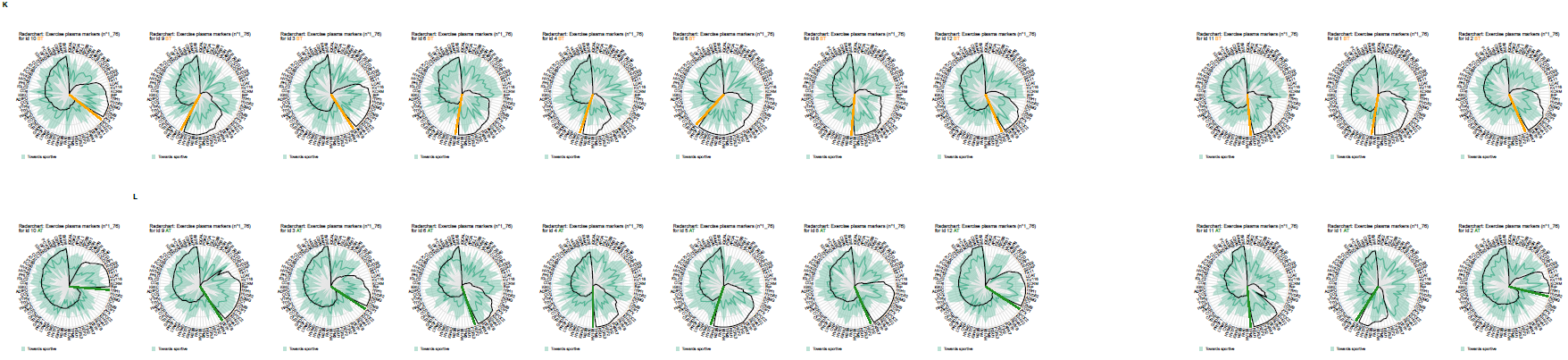


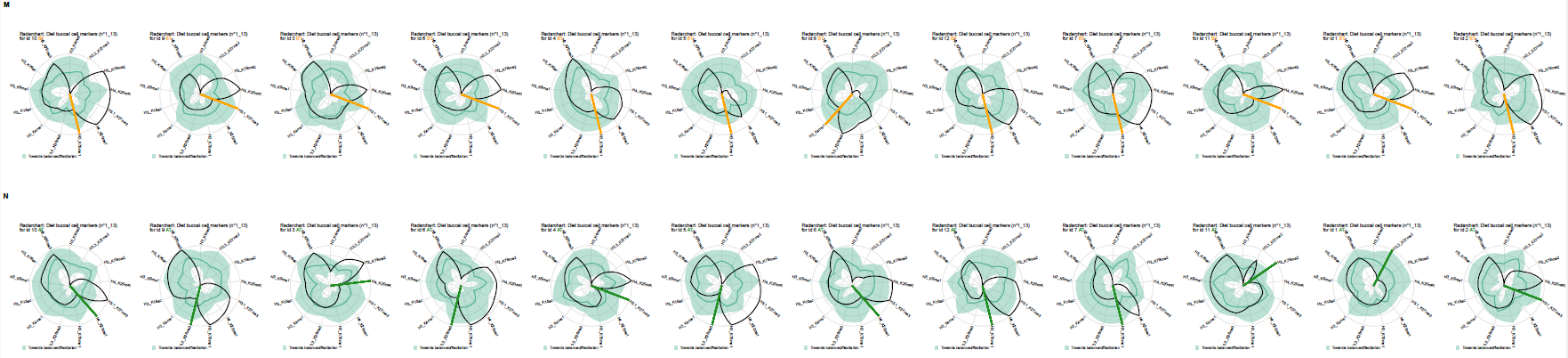


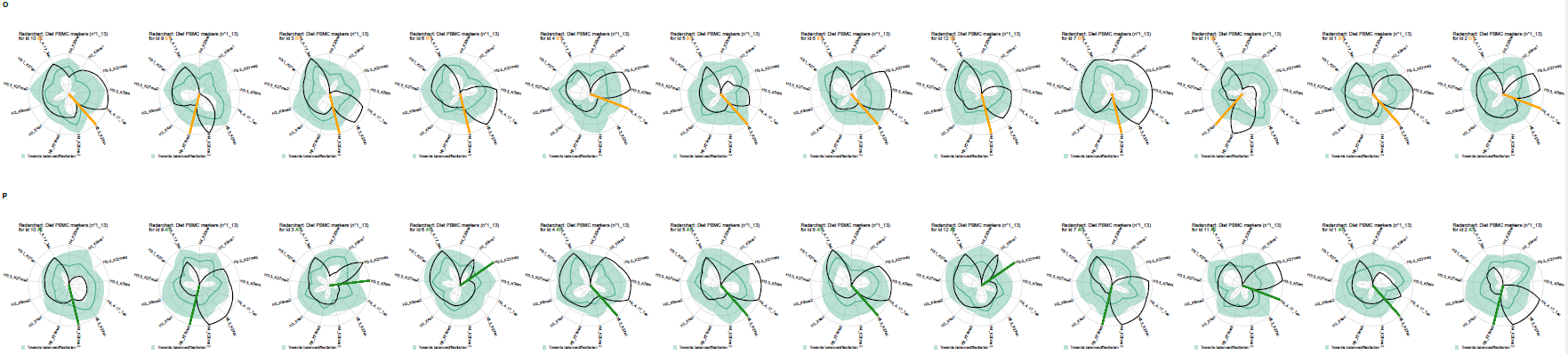


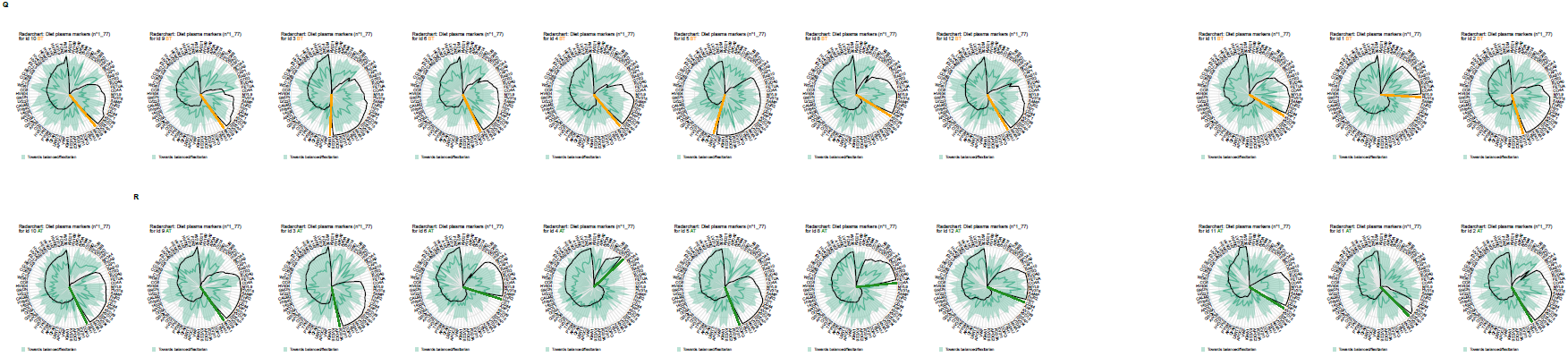


**Figure S20**. Radar charts from each participant (column) from all biological samples – buccal cells (**A-B, G-H, M-N**), PBMCs (**C-D, I-J, O-P**), and plasma (**E-F, K-L, Q-R**) - and for BMI (**A-E**), Exercise (**G-L**), and Dietary habits (**M-R**) classes´ markers (x-axis) before (**A, C, E, G, I, K, M, O, Q**) and after (**B, D, F, H, J, L, N, P, R**) therapy. For each radar chart, clockwise, we ordered health-awareness indicators from decreasing distance (normalized markers values represented as a black line, y-axis) to the healthy range (green ribbon, median as plain line). Orange/green line indicates the point when indicators display discrepancy with the healthy range, BT/AT respectively. The more within the upper right quadrant, the more similar the profile is to the healthier category of participants. For the plasma-originated radar charts, one individual was not represented because of a lack of paired data.


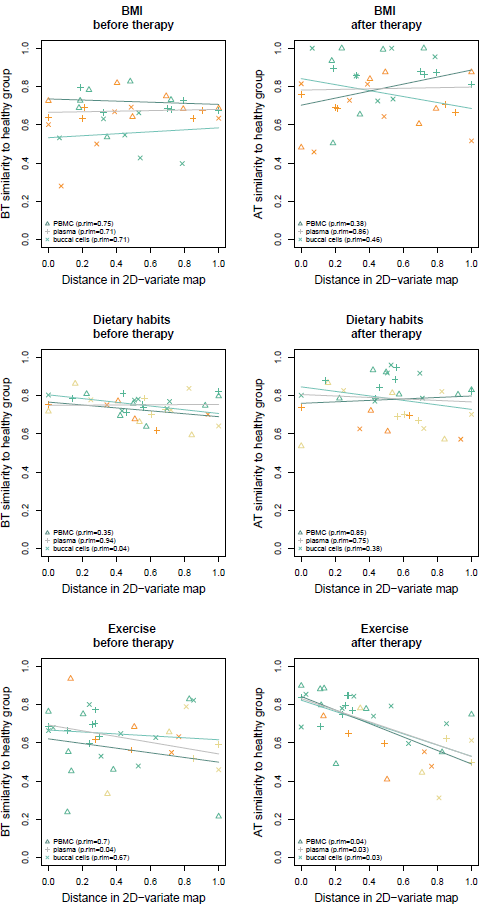


**Figure S21.** Scatterplots of the therapy effect size (x-axis) versus the similarity to the healthier category (y-axis) BT (first column) and AT (second column) across sample types (dot symbol, line colors) and BT BMI and lifestyle categories (rows). Per panel, the line represents the outcome of a linear model by robust regression performed by sample type (Fig. S16).

**References**

Brunson, J. C., & Read, Q. D. (2023). *ggalluvial: Alluvial Plots in “ggplot2.”*

Bushel, P. (2022). *pvca: Principal Variance Component Analysis (PVCA)*.

Kassambara, A., & Mundt, F. (2020). *Extract and Visualize the Results of Multivariate Data Analyses [R package factoextra version 1.0.7]*.

Maechler, M. (2022). *sfsmisc: Utilities from “Seminar fuer Statistik” ETH Zurich*.

Venables, W. N., & Ripley, B. D. (2002). *Modern Applied Statistics with S* (Fourth). Springer.

**
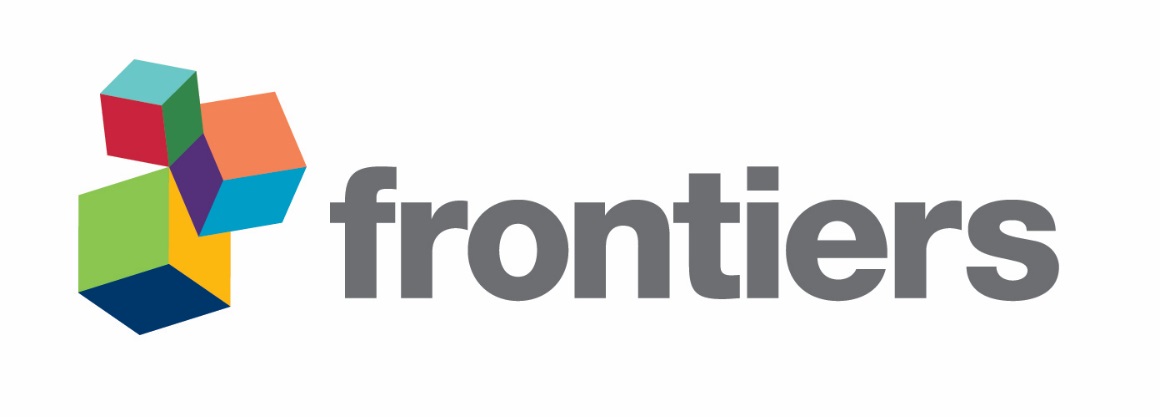
**
